# An Fe(II)-catalyzed synthesis of spiro[indoline-3,2'-pyrrolidine] derivatives

**DOI:** 10.3762/bjoc.21.183

**Published:** 2025-11-05

**Authors:** Elizaveta V Gradova, Nikita A Ozhegov, Roman O Shcherbakov, Alexander G Tkachenko, Larisa Y Nesterova, Elena Y Mendogralo, Maxim G Uchuskin

**Affiliations:** 1 Perm State University, Bukireva St. 15, 614990 Perm, Russian Federation; 2 Institute of Ecology and Genetics of Microorganisms, Perm Federal Research Center, The Ural Branch of Russian Academy of Sciences, Goleva St. 13, 614081 Perm, Russian Federation

**Keywords:** α,β-unsaturated ketone, dearomatization, indole, spirocyclization, spiro[indoline-3,2'-pyrrolidine]

## Abstract

A synthetic strategy for the preparation of spiro[indoline-3,2'-pyrrolidine] derivatives has been developed, featuring a two-step sequence consisting of the reaction of 2-arylindoles with α,β-unsaturated ketones, followed by Fe(II)-catalyzed spirocyclization of readily accessible oxime acetates. The method exhibits a broad substrate scope and good functional group tolerance. The synthesized spirocyclic compounds showed no significant antimicrobial activity.

## Introduction

**Figure 1:**
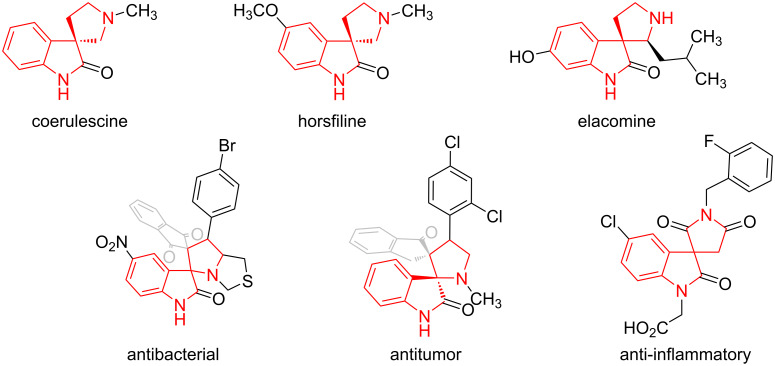
Natural and synthetic bioactive spiro[indoline-3,2'-pyrrolidine] derivatives.

Spiro[indoline-3,2'-pyrrolidine] derivatives represent an important class of organic compounds found in both natural products (e.g., coerulescine [1], horsfiline [2], and elacomine [3]) and synthetic bioactive molecules (Figure 1). This scaffold exhibits a broad range of pharmacological activities, including significant in vitro antimycobacterial properties [4], potent antitumor effects against melanoma cell lines [5], and antagonism of T_H_^2^ lymphocyte function [6]. Due to their wide-ranging biological activities, spiro[indoline-3,2'-pyrrolidines] have attracted substantial interest in medicinal chemistry, prompting the development of diverse synthetic strategies.

**Scheme 1:**
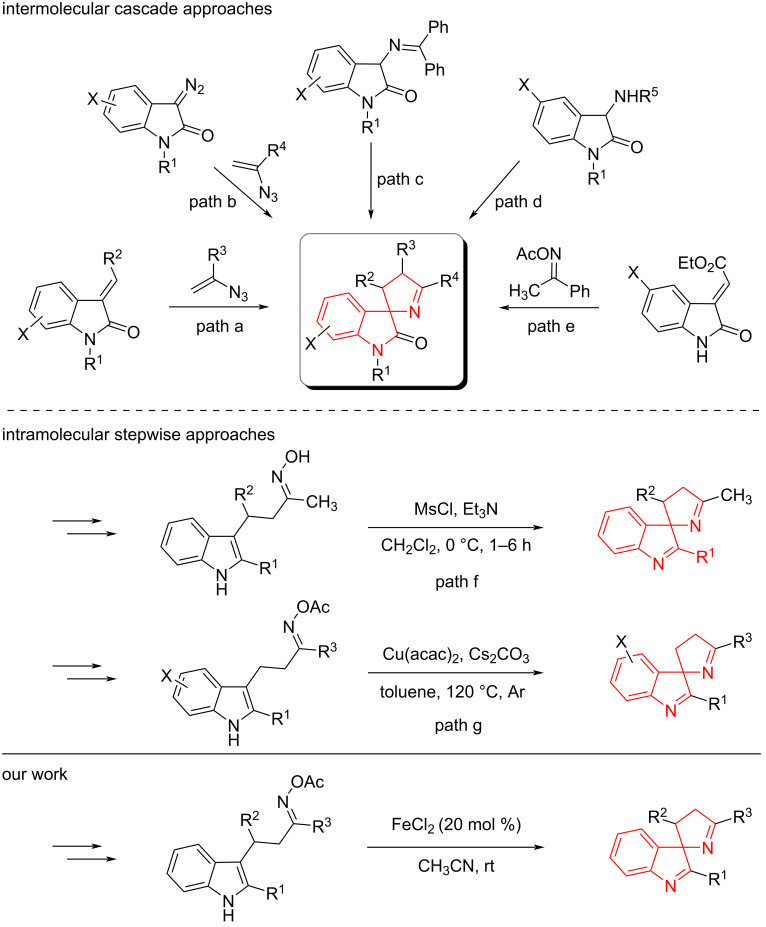
Previous approaches and our work.

Several methodologies have been reported for the synthesis of substituted spiro[indoline-3,2'-pyrrolidines] (Scheme 1), which can be broadly categorized into two main approaches. The first involves cascade reactions featuring the formation of multiple bonds, including a pivotal spirocyclization step. However, these strategies typically require pre-functionalized starting materials and often result exclusively in substituted oxindoles. For example, a highly diastereoselective method for the synthesis of dihydrospiro[indoline-3,2'-pyrrole]-2-ones has been developed (Scheme 1, path a) [7]. This transformation proceeds via a Lewis acid-mediated conjugate addition of vinyl azides to electron-deficient alkenes, followed by denitrogenative cyclization. Subsequently Zhong et al. reported a catalytic asymmetric variant, affording spirooxindoles in high yields with excellent enantioselectivity [8]. An alternative approach employing vinyl azides involves a Rh(II)-catalyzed olefination of diazo compounds, followed by annulation with vinyl azides to yield substituted spiropyrrolidines (Scheme 1, path b) [9]. Additionally, an organocatalytic, enantioselective Michael addition/cyclization sequence of 3-aminooxindole Schiff bases with terminal vinyl ketones, catalyzed by a cinchona-derived base, has been reported to afford chiral spiroindolylpyrroles in high yields (Scheme 1, path c) [10]. Further expanding the scope of enantioselective approaches, a Michael/cyclization cascade reaction between 3-aminooxindoles and 2-enoylpyridines, catalyzed by a cinchonidine-based thiourea organocatalyst, was developed. Subsequent treatment with HCl in methanol under heating furnished chiral spiroindolylpyrroles in excellent yields and enantioselectivity (Scheme 1, path d) [11]. Moreover, a copper-catalyzed reaction of oxindole-derived alkenes with acetophenone *O*-acetyl oxime has also been employed to construct the spiroindolylpyrrole scaffold (Scheme 1, path e) [12].

The second category of synthetic methods relies on more accessible, non-pre-functionalized starting materials and stepwise assembly of the spirocyclic core. This strategy enables the synthesis of functionalized 3*H*-indoles, which can be further elaborated into structurally diverse products. For instance, an intramolecular S*_N_*2-type cyclization of β-3-indolyl ketone oximes using MsCl and Et₃N affords spiro[indoline-3,2'-pyrrolidines] (Scheme 1, path f) [13]. A subsequent Cu-catalyzed spirocyclization of β-3-indolyl ketone oxime acetates was developed. This process involves homolytic N–O bond cleavage to generate an *N*-imidoyl radical intermediate that undergoes intramolecular cyclization to yield the spirocyclic product (Scheme 1, path g) [14]. Notably, iron is known to exhibit similar behavior in single-electron transfer (SET) processes [15–17]. In fact, we previously demonstrated an Fe-catalyzed dearomatization of β-2-furyl ketone oxime acetates, yielding functionalized pyrroles [18]. Herein, we report our investigation of the Fe-catalyzed spirocyclization of β-3-indolyl ketone oxime acetates.

## Results and Discussion

**Scheme 2:**
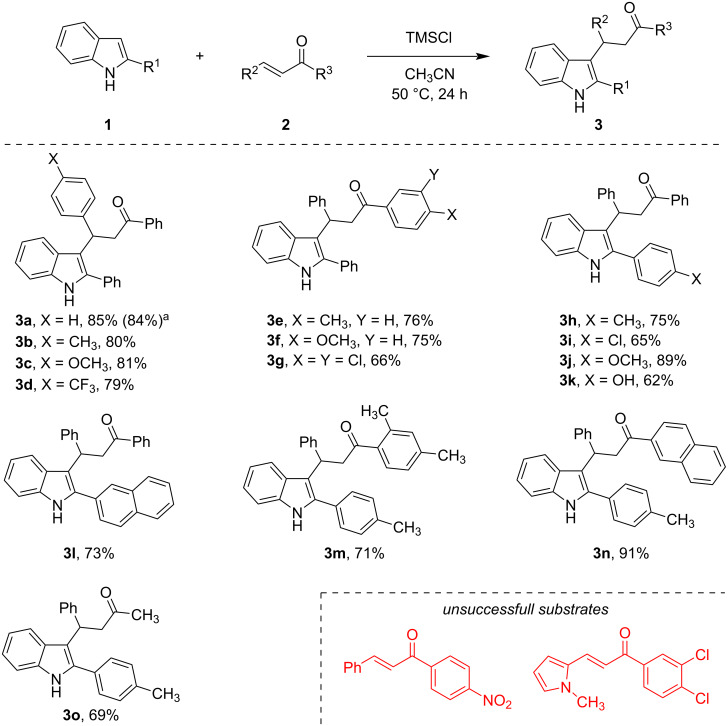
The reaction of 2-arylindoles **1** with α,β-unsaturated ketones **2**. ^a^Isolated yield of the 5 mmol scale experiment.

Our study commenced with the synthesis of key propan-1-one derivatives bearing an indolyl moiety. For this purpose, the reaction of 2-arylindoles **1** with α,β-unsaturated ketones **2** was employed, affording the corresponding indolylalkanones **3** (Scheme 2) [19–21]. A combination of trimethylsilyl chloride and acetonitrile served as a mild promoter for the desired reaction [17]. Under these conditions, the reaction was performed on a 5 mmol scale without any loss of efficiency. We next evaluated the scope of the developed protocol. The electronic and steric nature of substituents had a minimal impact on the product yields and in most cases, the desired products were obtained in good to high yields. However, two notable exceptions were identified. First, when a nitro group was introduced at the *para*-position of the benzoyl moiety the starting chalcone was recovered unchanged. Second, the use of an α,β-unsaturated ketone bearing an *N*-methylpyrrole moiety resulted in extensive decomposition and tarring, a common issue with α-unsubstituted pyrroles under acidic conditions. These results indicate that substrates featuring strongly electron-withdrawing groups or acid-sensitive motifs are not compatible with the developed protocol.

**Scheme 3:**
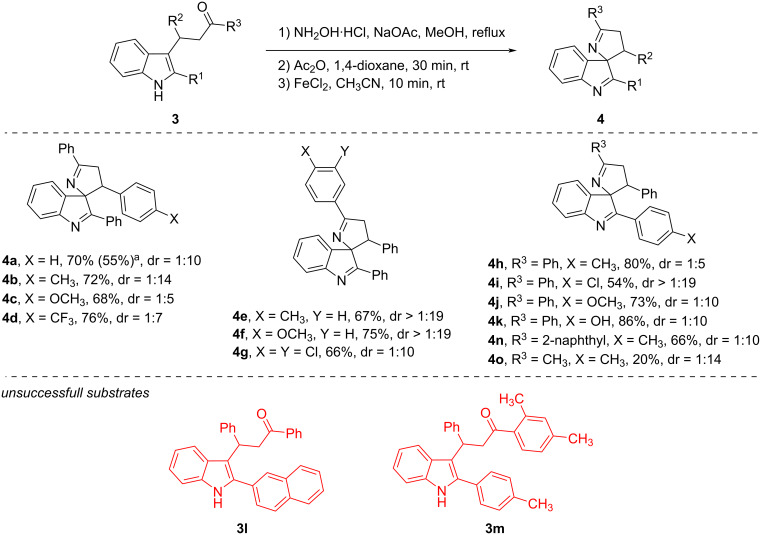
The scope of the Fe-catalyzed spirocyclization. ^a^Isolated yield of the 4.2 mmol scale experiment.

Next, we synthesized the model ketone oxime acetate from β-3-indolyl ketone **3a** using the previously described telescopic protocol [17]. This substrate was subjected to FeCl_2_-catalyzed spirocyclization in acetonitrile at room temperature, affording the desired spirocyclic product **4a** in 70% yield as a mixture of diastereomers (dr = 1:10). Scaling up the reaction to a 4 mmol scale led to a decrease in yield to 55%. Under these conditions, we explored the substrate scope of the reaction (Scheme 3). In general, substituent effects were minimal, and most spirocyclic products were obtained in good to high yields. Nevertheless, several notable exceptions were observed. First, when a substrate bearing an alkyl substituent at the keto group was employed, the yield of the desired product **4o** decreased to 20%. Second, the use of a 2-naphthylindole substrate (**3l**) afforded no desired product, presumably due to increased steric demand. Third, the introduction of an *ortho*-methyl substituent on the ketone moiety (**3m**) likewise suppressed product formation, likely due to steric hindrance interfering with cyclization at the C3 position of the indole ring.

**Scheme 4:**
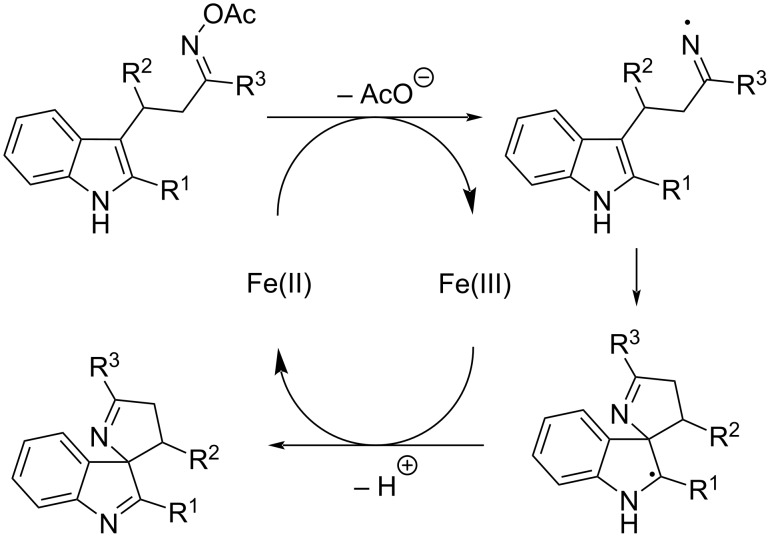
The proposed mechanism of product **4** formation.

Based on literature precedents [15–16], we propose a mechanism involving a radical pathway (Scheme 4). Initial Fe(II)-mediated reductive cleavage of the N–O bond in the ketoxime acetate generates an iminyl radical. This is followed by a 5-*exo*-*trig* cyclization to form a carbon-centered radical. Final single-electron oxidation by Fe(III) delivers the desired spirocyclic product.

All synthesized spiro[indoline-3,2'-pyrrolidine] derivatives **4** were evaluated for antimicrobial activity via serial dilution assays across a concentration range of 0.5–1000 µg/mL. The minimum inhibitory concentration (MIC) and minimum bactericidal (fungicidal) concentration (MBC/MFC) against a diverse spectrum of microorganisms was determined, including two fungal strains: *Candida albicans* ATCC 10231, *C. albicans* C1 – clinical strain, Gram-positive bacteria such as *Staphylococcus aureus* ATCC 25923, *S. aureus* ATCC 43300 (MRSA), *Mycobacterium smegmatis* ATCC 70084, and Gram-negative bacteria including *Escherichia coli* ATCC 25922, *E. coli* ATCC 8739, and *Klebsiella pneumoniae* ATCC 700603. Notably, none of the tested compounds demonstrated significant antibacterial activity against the evaluated strains (for details, see Supporting Information File 1).

## Conclusion

In summary, we have developed an efficient synthetic strategy for constructing spiro[indoline-3,2'-pyrrolidine] derivatives via a sequence involving the reaction of 2-arylindoles with α,β-unsaturated ketones, followed by Fe(II)-catalyzed spirocyclization of the corresponding easily accessible oxime acetates. The methodology exhibits broad substrate scope, with only minor limitations attributable to steric hindrance or functional group sensitivity. Antimicrobial evaluation of the synthesized spirocyclic compounds revealed no significant activity against the tested microbial strains. However, the synthetic versatility and broad substrate scope of developed protocol highlights its potential for further derivatization and the discovery of valuable properties.

## Supporting Information

File 1 General reaction procedures, compound characterization data, and copies of NMR spectra.

## Data Availability

All data that supports the findings of this study is available in the published article and/or the supporting information of this article.

## References

[1] Cossins E A, Chen L (1997). Phytochemistry.

[2] Jossang A, Jossang P, Hadi H A, Sevenet T, Bodo B (1991). J Org Chem.

[3] Pellegrini C, Weber M, Borschberg H-J (1996). Helv Chim Acta.

[4] Maheswari S U, Balamurugan K, Perumal S, Yogeeswari P, Sriram D (2010). Bioorg Med Chem Lett.

[5] Girgis A S, Panda S S, Srour A M, Farag H, Ismail N S M, Elgendy M, Abdel-Aziz A K, Katritzky A R (2015). Org Biomol Chem.

[6] Crosignani S, Page P, Missotten M, Colovray V, Cleva C, Arrighi J-F, Atherall J, Macritchie J, Martin T, Humbert Y (2008). J Med Chem.

[7] Zhu X, Chiba S (2016). Chem Commun.

[8] Zhong Z, Xiao Z, Liu X, Cao W, Feng X (2020). Chem Sci.

[9] Yi R, Qian L, Wan B (2019). Chin J Catal.

[10] Huang Z-C, Zou Y, Xiang M, Li C-Y, Li X, Tian F, Wang L-X (2021). Org Lett.

[11] Cui B, Chen Y, Shan J, Qin L, Yuan C, Wang Y, Han W, Wan N, Chen Y (2017). Org Biomol Chem.

[12] Zhao B, Liang H-W, Yang J, Yang Z, Wei Y (2017). ACS Catal.

[13] Tanaka K, Mori Y, Narasaka K (2004). Chem Lett.

[14] Wang P-F, Chen C, Chen H, Han L-S, Liu L, Sun H, Wen X, Xu Q-L (2017). Adv Synth Catal.

[15] Zhu Z, Tang X, Li J, Li X, Wu W, Deng G, Jiang H (2017). Org Lett.

[16] Wu C, Liu T-X, Zhang P, Zhu X, Zhang G (2020). Org Lett.

[17] Liang W, Jiang K, Du F, Yang J, Shuai L, Ouyang Q, Chen Y-C, Wei Y (2020). Angew Chem, Int Ed.

[18] Makarov A S, Fadeev A A, Uchuskin M G (2021). Org Chem Front.

[19] Fadeev A A, Uchuskin M G, Trushkov I V, Makarov A S (2017). Chem Heterocycl Compd.

[20] Liu J, Zhang Y, Yue Y, Wang Z, Shao H, Zhuo K, Lv Q, Zhang Z (2019). J Org Chem.

[21] Patel T, Gaikwad R, Jain K, Ganesh R, Bobde Y, Ghosh B, Das K, Gayen S (2019). ChemistrySelect.

