# Supplementary material for: An Fe(II)-catalyzed synthesis of spiro[indoline-3,2'-pyrrolidine] derivatives
Source: Beilstein J Org Chem. 2025 Nov 5;21:2383–8. doi: 10.3762/bjoc.21.183 (PMC12599403; doi:10.3762/bjoc.21.183)

BEILSTEIN JOURNAL OF ORGANIC CHEMISTRY

Supporting Information

for

An Fe(II)-catalyzed synthesis of spiro[indoline-3,2'-pyrrolidine] derivatives

Elizaveta V. Gradova, Nikita A. Ozhegov, Roman O. Shcherbakov,
Alexander G. Tkachenko, Larisa Y. Nesterova, Elena Y. Mendogralo
and Maxim G. Uchuskin

Beilstein J. Org. Chem. **2025,** *21,* 2383–2388. doi:10.3762/bjoc.21.183

General reaction procedures, compound characterization data, and copies of NMR spectra

License and Terms: This is a supporting information file under the terms of the Creative Commons Attribution License (https://creativecommons.org/licenses/by/4.0). Please note that the reuse, redistribution and reproduction in particular requires that the author(s) and source are credited and that individual graphics may be subject to special legal provisions.
The license is subject to the *Beilstein Journal of Organic Chemistry* terms and conditions: (https://www.beilstein-journals.org/bjoc/terms)

Table of contents

1. General information S2

2. Experimental procedures S3

3. Antimicrobial activity S10

4. References S12

5. Copies of NMR spectra S13

1. General information

^{1}H and $^{13}C\{^{1}H\}$ NMR spectra were recorded with a Bruker Avance III HD 400 (400 MHz for ^{1}H, 100 MHz for ^{13}C NMR, 376 MHz for ^{19}F NMR) spectrometer at 40 °C. The chemical shifts (δ) were measured in ppm with respect to the solvent ($CDCl_3$, ^{1}H: δ = 7.26 ppm, ^{13}C: δ = 77.16 ppm; DMSO-d_6, ^{1}H: δ = 2.50 ppm, ^{13}C: δ = 39.52 ppm). The coupling constants (*J*) are given in hertz (Hz). The splitting patterns of apparent multiplets associated with the averaged coupling constants were designated as s (singlet), d (doublet), t (triplet), m (multiplet), dd (doublet of doublets) and br (broadened). High-resolution mass measurements (HRMS) were carried out using a BrukermicrOTOF-Q^{TM} ESI-TOF (electro spray ionization/time of flight) mass spectrometer. GC–MS analysis was performed on an «Agilent 7890B» interfaced to an Agilent 5977A mass selective detector. The melting points were determined with a Stuart SMP 30. Column chromatography was performed on Macherey Nagel silica gel (40–63 μm). All the reactions were carried out using freshly distilled and dry solvents from solvent stills. The starting 3-(2-aryl-1*H*-indol-3-yl)propan-1-ones were obtained in a manner similar to the described procedure. [1]

2. Experimental procedures

General procedure for the synthesis of 3-(2-aryl-1*H*-indol-3-yl)propan-1-ones 3

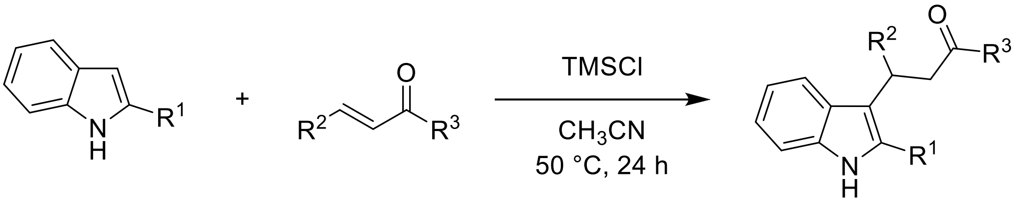

1 **2** **3**

To a solution of corresponding indole **1** (2 mmol) and α,β-unsaturated ketone **2** (2 mmol) in CH_3CN (10 mL) was added TMSCl (1 mmol). The resulting solution was stirred at 50 °C in the aluminum block for ca. 24 h (TLC control). Upon completion, the reaction mixture was concentrated in vacuo. The product was purified by column chromatography (silica gel, petroleum ether/ethyl acetate 50:1 (v/v)) for compounds **3a**,**b**, **3d**,**e**, **3g–i**, **3l–n** or petroleum ether/ethyl acetate 20:1 (v/v) for compounds **3c**, **3f**, **3j**,**k**, and **3o**).

1,3-Diphenyl-3-(2-phenyl-1*H*-indol-3-yl)propan-1-one (3a).[2] Yield 682 mg (85%), yellow oil. ^{1}H NMR ($CDCl_3$, 400 MHz) δ = 8.06 (br s, 1H), 7.85–7.83 (m, 2H), 7.66–7.64 (m, 1H), 7.53–7.47 (m, 3H), 7.44–7.33 (m, 8H), 7.29–7.26 (m, 2H), 7.22–7.17 (m, 2H), 7.13–7.09 (m, 1H), 5.36 (t, *J* = 7.0 Hz, 1H), 4.00–3.92 (m, 2H) ppm; ^{13}C{^{1}H} NMR ($CDCl_3$, 100 MHz): δ = 198.8, 144.7, 137.2, 136.4, 135.7, 133.2, 132.9, 128.91 (2C), 128.86 (2C), 128.5 (4C), 128.2 (3C), 128.0, 127.8 (2C), 126.1, 122.2, 120.7, 119.9, 114.9, 111.3, 44.6, 37.1 ppm.

1-Phenyl-3-(2-phenyl-1*H*-indol-3-yl)-3-(*p*-tolyl)propan-1-one (3b).[2] Yield 664 mg (80%), yellow oil. ^{1}H NMR ($CDCl_3$, 400 MHz) δ = 7.89 (br s, 1H), 7.70–7.68 (m, 2H), 7.53–7.51 (m, 1H), 7.40–7.19 (m, 9H), 7.16–7.14 (m, 2H), 7.08–7.05 (m, 1H), 6.99–6.94 (m, 3H), 5.17 (t, *J* = 7.0 Hz, 1H), 3.84–3.76 (m, 2H), 2.18 (s, 3H) ppm; ^{13}C{^{1}H} NMR ($CDCl_3$, 100 MHz): = δ 198.9, 141.7, 137.3, 136.5, 135.6, 135.5, 133.3 132.8, 129.2 (2C), 128.93 (2C), 128.87 (2C), 128.5 (2C), 128.2 (3C), 128.1, 127.6 (2C), 122.2, 120.8, 119.9, 115.1, 111.2, 44.8, 36.8, 21.1 ppm.

3-(4-Methoxyphenyl)-1-phenyl-3-(2-phenyl-1*H*-indol-3-yl)propan-1-one (3c).[2] Yield 698 mg (81%), yellow oil. ^{1}H NMR ($CDCl_3$, 400 MHz): δ = 7.95 (br s, 1H), 7.74–7.72 (m, 2H), 7.56–7.54 (m, 1H), 7.43–7.20 (m, 11H), 7.13–7.09 (m, 1H), 7.04–7.00 (m, 1H), 6.73–6.71 (m, 2H), 5.19 (t, *J* = 7.0 Hz, 1H), 3.87 (dd, *J* = 16.8, 7.0 Hz, 1H), 3.80 (dd, *J* = 16.8, 7.0 Hz, 1H), 3.69 (s, 3H) ppm; ^{13}C{^{1}H} NMR ($CDCl_3$, 100 MHz) δ = 199.0, 158.0, 137.3, 136.9, 136.5, 135.6, 133.3, 132.9, 128.91 (2C), 128.88 (2C), 128.7 (2C), 128.5 (2C), 128.2 (3C), 128.0, 122.2, 120.8, 119.9, 115.2, 114.0 (2C), 111.2, 55.4, 44.9, 36.4 ppm.

1-Phenyl-3-(2-phenyl-1*H*-indol-3-yl)-3-[4-(trifluoromethyl)phenyl]propan-1-one (3d).[2] Yield 741 mg (79%), yellow oil. ^{1}H NMR ($CDCl_3$, 400 MHz): δ = 8.07 (br s, 1H), 7.84–7.82 (m, 2H), 7.58–7.56 (m, 1H), 7.52–7.35 (m, 13H), 7.23–7.19 (m, 1H), 7.13–7.09 (m, 1H), 5.36 (t, *J* = 7.0 Hz, 1H), 3.99 (dd, *J* = 17.2, 7.0 Hz, 1H), 3.9180 (dd, *J* = 17.2, 7.0 Hz, 1H) ppm; ^{13}C{^{1}H} NMR ($CDCl_3$, 100 MHz): δ = 198.0, 148.6 (br s), 136.7, 136.1, 135.6, 132.9, 132.7, 128.7 (2C), 128.6 (2C), 128.4 (2C), 128.2, 128.18 (q, J_{CF} = 33.2 Hz), 127.9 (2C), 127.8 (2C), 127.4, 125.1 (q, J_{CF} = 3.8 Hz, 2C), 124.2 (q, J_{CF} = 271.8 Hz), 122.2, 120.1, 119.9, 113.9, 111.1, 44.1, 36.7 ppm; ^{19}F NMR ($CDCl_3$, 376 MHz) δ = -67.21 (s) ppm.

3-Phenyl-3-(2-phenyl-1*H*-indol-3-yl)-1-(*p*-tolyl)propan-1-one (3e).[2] Yield 631 mg (76%), yellow oil. ^{1}H NMR ($CDCl_3$, 400 MHz): δ = 8.03 (br s, 1H), 7.73–7.71 (m, 2H), 7.62–7.60 (m, 1H), 7.52–7.49 (m, 2H), 7.44–7.34 (m, 6H), 7.25–7.22 (m, 2H), 7.18–7.12 (m, 4H), 7.09–7.05 (m, 1H), 5.32 (t, *J* = 7.0 Hz, 1H), 3.94 (dd, *J* = 16.8, 7.0 Hz, 1H), 3.86 (dd, *J* = 16.8, 7.0 Hz, 1H), 2.36 (s, 3H) ppm; ^{13}C{^{1}H} NMR ($CDCl_3$, 100 MHz): δ = 198.4, 144.8, 143.6, 136.5, 135.7, 134.8, 133.3, 129.2 (2C), 128.94 (2C), 128.89 (2C), 128.5 (2C), 128.3 (2C), 128.2, 128.1, 127.8 (2C), 126.1, 122.2, 120.8, 119.9, 115.1, 111.2, 44.5, 37.1, 21.7 ppm.

1-(4-Methoxyphenyl)-3-phenyl-3-(2-phenyl-1*H*-indol-3-yl)propan-1-one (3f).[2] Yield 647 mg (75%), yellow oil. ^{1}H NMR ($CDCl_3$, 400 MHz): δ = 7.93 (br s, 1H), 7.70–7.68 (m, 2H), 7.51–7.49 (m, 1H), 7.40–7.38 (m, 2H), 7.32–7.23 (m, 6H), 7.15–7.11 (m, 2H), 7.09–7.05 (m, 2H), 6.99–6.95 (m, 1H), 6.71–6.68 (m, 2H), 5.20 (t, *J* = 7.0 Hz, 1H), 3.83–3.69 (m, 2H), 3.71 (s, 3H) ppm; ^{13}C{^{1}H} NMR ($CDCl_3$, 100 MHz): δ = 197.4, 163.4, 144.8, 136.5, 135.7, 133.3, 130.5 (2C), 130.4, 128.93 (2C), 128.89 (2C), 128.5 (2C), 128.2, 128.1, 127.8 (2C), 126.0, 122.2, 120.8, 119.9, 115.1, 113.7 (2C), 111.2, 55.5, 44.3, 37.2 ppm.

1-(3,4-Dichlorophenyl)-3-phenyl-3-(2-phenyl-1*H*-indol-3-yl)propan-1-one (3g). Yield 619 mg (66%), yellow oil, ^{1}H NMR ($CDCl_3$, 400 MHz): δ = 7.87 (d, *J* = 2.0 Hz, 1H), 7.63 (d, *J* = 2.0 Hz, 1H), 7.44–7.41 (m, 1H), 7.39–7.36 (m, 1H), 7.30–7.19 (m, 9H), 7.16–7.13 (m, 2H) 7.08–7.03 (m, 2H), 6.97–6.93 (m, 1H), 5.10 (t, *J* = 7.2 Hz, 1H), 3.75 (dd, *J* = 16.2, 7.2 Hz, 1H), 3.64 (dd, *J* = 16.2, 7.2 Hz, 1H) ppm; ^{13}C{^{1}H} NMR ($CDCl_3$, 100 MHz): δ = 196.9, 144.2, 137.4, 136.7, 136.4, 135.9, 133.2, 133.0, 130.5, 130.2, 128.9 (2C), 128.8 (2C), 128.7 (2C), 128.4, 127.8, 127.7 (2C), 127.1, 126.4, 122.4, 120.6, 120.1, 114.0, 111.3, 44.5, 37.6 ppm. HRMS (ESI$^+$) m/z: [M+H]$^+$ Calcd for $C_{29}H_{22}Cl_2NO^+$ 470.1073; found 470.1070.

1,3-Diphenyl-3-[2-(*p*-tolyl)-1*H*-indol-3-yl]propan-1-one (3h). Yield 623 mg (75%), yellow oil. ^{1}H NMR ($CDCl_3$, 400 MHz): δ = 8.02 (br s, 1H), 7.85–7.83 (m, 2H), 7.64–7.62 (m, 1H), 7.52–7.49 (m, 1H), 7.43–7.35 (m, 7H), 7.30–7.18 (m, 6H), 7.12–7.09 (m, 1H), 5.34 (t, *J* = 7.0 Hz, 1H), 3.99 (dd, *J* = 16.8, 7.0 Hz, 1H), 3.92 (dd, *J* = 16.8, 7.0 Hz, 1H), 2.43 (s, 3H) ppm; ^{13}C{^{1}H} NMR ($CDCl_3$, 100 MHz): δ = 198.8, 144.8, 138.1, 137.3, 136.4, 135.8, 132.8, 130.3, 129.6 (2C), 128.8 (2C), 128.5 (4C), 128.2 (2C), 128.1, 127.8 (2C), 126.1, 122.0, 120.7, 119.8, 114.6, 111.2, 44.6, 37.2, 21.4 ppm. HRMS (ESI$^+$) m/z: [M+Na]$^+$ Calcd for $C_{30}H_{25}NNaO^+$ 438.1828; found 438.1825.

3-[2-(4-Chlorophenyl)-1*H*-indol-3-yl]-1,3-diphenylpropan-1-one (3i). Yield 566 mg (65%), yellow oil, ^{1}H NMR ($CDCl_3$, 400 MHz) δ = 7.97 (br s, 1H), 7.72–7.70 (m, 2H), 7.54–7.52 (m, 1H), 7.42–7.37 (m, 1H), 7.32–7.30 (m, 2H), 7.27–7.23 (m, 7H), 7.18–7.15 (m, 2H), 7.11–7.06 (m, 2H), 7.02–6.98 (m, 1H), 5.16 (t, *J* = 7.0 Hz, 1H), 3.88–3.80 (m, 2H) ppm; ^{13}C{^{1}H} NMR ($CDCl_3$, 100 MHz): δ = 198.8, 144.5, 137.0, 136.5, 134.5, 134.2, 133.0, 131.6, 130.1 (2C), 129.1 (2C), 128.60 (2C), 128.57 (2C), 128.1 (2C), 127.8, 127.7 (2C), 126.2, 122.4, 120.8, 120.1, 115.1, 111.4, 44.6, 37.1 ppm. HRMS (ESI$^+$) m/z: [M+H]$^+$ Calcd for $C_{29}H_{23}ClNO^+$ 436,1463; found 436.1464.

3-[2-(4-Methoxyphenyl)-1*H*-indol-3-yl]-1,3-diphenylpropan-1-one (3j). Yield 767 mg (89%), yellow oil, ^{1}H NMR ($CDCl_3$, 400 MHz): δ = 7.89 (br s, 1H), 7.73–7.71 (m, 2H), 7.52–7.79 (m, 1H), 7.40–7.36 (m, 1H), 7.32–7.22 (m, 7H), 7.17–7.13 (m, 2H), 7.09–7.05 (m, 2H), 7.00–6.96 (m, 1H), 6.86–6.82 (m, 2H), 5.19 (t, *J* = 7.0 Hz, 1H), 3.83 (dd, *J* = 6.8, 0.8 Hz, 2H), 3.74 (s, 3H) ppm; ^{13}C{^{1}H} NMR ($CDCl_3$, 100 MHz,) δ = 198.9, 159.7, 144.9, 137.3, 136.3, 135.7, 132.9, 130.2 (2C), 128.51 (2C), 128.49 (2C), 128.2 (2C), 128.1, 127.8 (2C), 126.1, 125.6, 121.9, 120.5, 119.8, 114.4 (2C), 114.3, 111.1, 55.5, 44.6, 37.2 ppm. HRMS (ESI$^+$) m/z: [M+H]$^+$ Calcd for $C_{30}H_{26}NO_2^+$ 432.1958; found 432.1964.

3-[2-(4-Hydroxyphenyl)-1*H*-indol-3-yl]-1,3-diphenylpropan-1-one (3k).Yield 517 mg (62%), yellow oil, ^{1}H NMR (DMSO-d_6, 400 MHz): δ = 10.98 (br s, 1H), 9.58 (br s, 1H), 7.93–7.89 (m, 2H), 7.62–7.55 (m, 2H), 7.47–7.42 (m, 2H), 7.40–7.36 (m, 2H), 7.32–7.26 (m, 3H), 7.23–7.18 (m, 2H), 7.13–7.08 (m, 1H), 7.04–7.00 (m, 1H), 6.93–6.86 (m, 3H), 5.12 (t, *J* = 7.2 Hz, 1H), 4.08 (dd, *J* = 17.2, 7.2 Hz, 1H), 3.93 (dd, *J* = 17.2, 7.2 Hz, 1H) ppm; ^{13}C{^{1}H} NMR (DMSO-d_6, 100 MHz): δ = 198.5, 157.1, 144.8, 136.6, 136.0, 135.5, 132.9, 129.9 (2C), 128.5 (2C), 128.0 (2C), 127.8 (2C), 127.2 (2C), 127.0, 125.5, 123.7, 120.5, 119.7, 118.5, 115.3 (2C), 112.5, 111.1, 43.1, 36.6 ppm. HRMS (ESI$^+$) m/z: [M+Na]$^+$ Calcd for $C_{29}H_{23}NNaO_2^+$ 440.1621; found 440.1615.

3-[2-(Naphthalen-2-yl)-1*H*-indol-3-yl]-1,3-diphenylpropan-1-one (3l). Yield 659 mg (73%), yellow oil, ^{1}H NMR (CDCl$_3$, 400 MHz): δ = 8.13 (br s, 1H), 7.92–7.90 (m, 1H), 7.87–7.84 (m, 2H), 7.79–7.75 (m, 3H), 7.66–7.61 (m, 2H), 7.52–7.48 (m, 2H), 7.42–7.36 (m, 4H), 7.28–7.24 (m, 4H), 7.21–7.15 (m, 2H), 7.12–7.07 (m, 1H), 5.40 (t, *J* = 7.0 Hz, 1H), 3.93 (d, *J* = 7.0 Hz, 2H) ppm; ^{13}C{^{1}H} NMR (CDCl$_3$, 100 MHz): δ = 198.8, 144.9, 137.2, 136.6, 135.7, 133.5, 133.1, 132.8, 130.6, 128.60, 128.57 (2C), 128.5 (2C), 128.4, 128.19, 128.15 (2C), 128.1, 127.9, 127.8 (2C), 126.6 (2C), 126.5, 126.2, 122.3, 120.8, 120.0, 115.4, 111.3, 44.7, 37.4 ppm. HRMS (ESI$^+$) m/z: [M+H]$^+$ Calcd for $C_{33}H_{26}NO^+$ 452.2009; found 452.2013.

1-(2,4-Dimethylphenyl)-3-phenyl-3-[2-(*p*-tolyl)-1*H*-indol-3-yl]propan-1-one (3m). Yield 629 mg (71%), yellow oil, ^{1}H NMR (CDCl$_3$, 400 MHz): δ = 7.90 (br s, 1H), 7.48–7.46 (m, 1H), 7.26–7.22 (m, 6H), 7.16–7.04 (m, 6H), 6.96–6.93 (m, 1H), 6.83 (br s, 1H), 6.78 (br d, *J* = 7.2 Hz, 1H), 5.12 (t, *J* = 7.6 Hz, 1H), 3.78 (dd, *J* = 16.4, 7.2 Hz, 1H), 3.64 (dd, *J* = 16.4, 7.2 Hz, 1H), 2.30 (s, 3H), 2.19 (s, 3H), 2.09 (s, 3H) ppm; ^{13}C{^{1}H} NMR (CDCl$_3$, 100 MHz): δ = 202.5, 144.8, 141.3, 138.3, 138.0, 136.3, 135.7, 135.6, 132.7, 130.2, 129.5 (2C), 128.7 (2C), 128.6, 128.5 (2C), 128.2, 127.8 (2C), 126.1, 126.0, 122.0, 120.8, 119.8, 114.5, 111.1, 47.5, 37.7, 21.4 (2C), 20.9 ppm. HRMS (ESI$^+$) m/z: [M+H]$^+$ Calcd for $C_{32}H_{30}NO^+$ 444.2322; found 444.2318.

1-(Naphthalen-2-yl)-3-phenyl-3-[2-(*p*-tolyl)-1*H*-indol-3-yl]propan-1-one (3n). Yield 846 mg (91%), yellow oil, ^{1}H NMR (CDCl$_3$, 400 MHz): δ = 8.13 (br s, 1H), 7.89 (br s, 1H), 7.80 (dd, *J* = 8.6, 1.8 Hz, 1H), 7.75–7.73 (m, 1H), 7.70–7.65 (m, 2H), 7.58–7.56 (m, 1H), 7.49–7.47 (m, 1H), 7.44–7.40 (m, 1H), 7.37–7.35 (m, 2H), 7.27–7.19 (m, 5H), 7.13–7.01 (m, 5H), 5.28 (t, *J* = 7.2 Hz, 1H), 3.99 (dd, *J* = 16.2, 7.2 Hz, 1H), 3.92 (dd, *J* = 16.2, 7.2 Hz, 1H), 2.27 (s, 3H) ppm; ^{13}C{^{1}H} NMR (CDCl$_3$, 100 MHz): δ = 199.0, 144.7, 138.1, 136.4, 135.9, 135.6, 134.6, 132.6, 130.2, 129.9, 129.7, 129.5 (2C), 128.7(2C), 128.5 (2C), 128.4, 128.2, 128.1, 127.80 (2C), 127.75, 126.6, 126.1, 124.1, 122.1, 120.7, 119.9, 114.4, 111.2, 44.6, 37.6, 21.3 ppm. HRMS (ESI$^+$) m/z: [M+H]$^+$ Calcd for $C_{34}H_{28}NO^+$ 466.2165; found 466.2160.

4-Phenyl-4-[2-(*p*-tolyl)-1*H*-indol-3-yl]butan-2-one (3o). Yield 487 mg (69%), yellow oil, ^{1}H NMR (CDCl$_3$, 400 MHz): δ = 7.97 (br s, 1H), 7.49–7.47 (m, 1H), 7.31–7.22 (m, 5H), 7.18–7.14 (m, 4H), 7.10–7.05 (m, 2H), 6.70–6.96 (m, 1H), 4.95 (t, *J* = 7.4 Hz, 1H), 3.33 (dd, *J* = 16.2, 7.4 Hz, 1H), 3.24 (dd, *J* = 16.2, 7.4 Hz, 1H), 2.32 (s, 3H), 1.85 (s, 3H); ^{13}C{^{1}H} NMR (CDCl$_3$, 100 MHz): δ = 207.7, 144.6, 138.2, 136.3, 136.0, 130.2, 129.7 (2C), 128.7 (2C), 128.5 (2C), 127.9, 127.6 (2C), 126.1, 122.1, 120.7, 119.8, 113.9, 111.2, 49.4, 37.2, 30.5, 21.4 ppm. HRMS (ESI$^+$) m/z: [M+H]$^+$ Calcd for $C_{25}H_{24}NO^+$ 354.1852; found 354.1855.

Synthesis of 3-(2-aryl-1*H*-indol-3-yl)propan-1-one 3a in 5 mmol scale

To a solution of indole **1a** (965 mg, 5 mmol) and α,β-unsaturated ketone **2a** (1040 mg, 5 mmol) in CH_3CN (25 mL) was added TMSCl (317 μl, 2.5 mmol). The resulting solution was stirred at 50 °C in the aluminum block for ca. 24 h (TLC control). Upon completion, the reaction mixture was concentrated in vacuo. Isolation by column chromatography on silica gel petroleum ether/ethyl acetate 20:1 (v/v) gave 1472 mg (84%) of 1,3-diphenyl-3-(2-phenyl-1*H*-indol-3-yl)propan-1-one (**3a**).

General procedure for the synthesis of 3',4'-dihydrospiro[indole-3,2'-pyrroles] 4

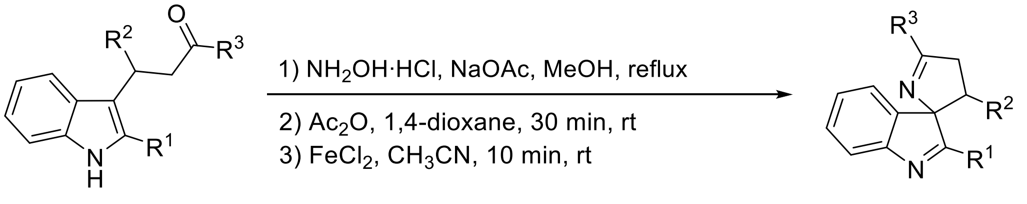

3 **4**

To a stirred solution of indole **3** (1 mmol) and NaOAc·3H_2O (1.2 mmol, 1.2 equiv) in MeOH (10 mL) was added NH_2OH·HCl (1.2 mmol, 1.2 equiv), and the reaction mixture was refluxed until full conversion of the starting material by TLC was observed (typically 5–12 h). Then, the solvent was evaporated to dryness in vacuo. The resulting oil was dissolved in 1,4-dioxane (20 mL), and Ac_2O (1.5 equiv)* was added in a single portion at ambient temperature. The reaction mixture was stirred for 30 min (TLC control). The reaction mixture was poured into water (100 mL), and the product was extracted with ethyl acetate (3 × 25 mL). The combined organic fractions were washed with water (2 × 25 mL), dried with anhydrous Na_2SO_4 and evaporated to dryness in vacuo. The residue was dissolved in CH_3CN (5 mL) and $FeCl_2$ (0.2 mmol) was added. The reaction mixture was stirred at room temperature for 10 min (TLC control). The products were purified by column chromatography (silica gel, petroleum ether/dichloromethane 3:1 (v/v) for compounds **4a–h**, **4k–m** or petroleum ether/ethyl acetate 20:1 (v/v) for compounds **4i,j**). The products were isolated as a mixture of diastereomers, and NMR spectra are given for the major isomer.

* for substrate **3k** 3 equiv of Ac_2O was used

2,3',5'-Triphenyl-3',4'-dihydrospiro[indole-3,2'-pyrrole] (4a). Yield 279 mg (70%), yellow oil. ^{1}H NMR ($CDCl_3$, 400 MHz): δ = 8.24–8.08 (m, 4H), 7.58–7.45 (m, 6H), 7.43–7.39 (m, 1H), 7.19–7.14 (m, 1H), 7.07–6.97 (m, 5H), 6.90–6.82 (m, 2H), 4.26 (dd, *J* = 11.4, 8.8 Hz, 1H), 3.86 (dd, *J* = 16.8, 11.4 Hz, 1H), 3.64 (dd, *J* = 16.8, 8.8 Hz, 1H) ppm; ^{13}C{^{1}H} NMR ($CDCl_3$, 100 MHz): δ = 179.3, 176.4, 153.8, 138.3, 135.9, 134.3, 133.8, 131.6, 131.0, 129.3, 129.0 (4C), 128.7 (2C), 128.2 (2C), 127.9 (2C), 127.3 (2C), 127.0, 125.5, 123.3, 121.0, 94.6, 52.6, 39.7 ppm. HRMS (ESI$^+$) m/z: $[M+H]^+$ Calcd for $C_{29}H_{23}N_2^+$ 399.1856; found 399.1844.

2,5'-Diphenyl-3'-(*p*-tolyl)-3',4'-dihydrospiro[indole-3,2'-pyrrole] (4b). Yield 297 mg (72%), yellow oil. ^{1}H NMR ($CDCl_3$, 400 MHz): δ = 8.16–8.07 (m, 4H), 7.57–7.47 (m, 6H), 7.43–7.41 (m, 1H), 7.20–7.16 (m, 1H), 7.06–7.00 (m, 2H), 6.83–6.81 (m, 2H), 6.75–6.73 (m, 2H), 4.22 (dd, *J* = 11.8, 8.6 Hz, 1H), 3.82 (dd, *J* = 16.8, 11.8 Hz, 1H), 3.61 (dd, *J* = 16.8, 8.6 Hz, 1H), 2.15 (s, 3H) ppm; ^{13}C{^{1}H} NMR ($CDCl_3$, 100 MHz): δ = 179.4, 176.4, 153.9, 138.4, 136.5, 134.4, 133.9, 132.8, 131.6, 130.9, 129.2, 128.94 (2C), 128.91 (2C), 128.72 (2C), 128.65 (2C), 128.2 (2C), 127.3 (2C), 125.5, 123.4, 121.1, 94.5, 52.4, 40.0, 21.0 ppm. HRMS (ESI$^+$) m/z: $[M+H]^+$ Calcd for $C_{30}H_{25}N_2^+$ 413.2012; found 413.2018.

3'-(4-Methoxyphenyl)-2,5'-diphenyl-3',4'-dihydrospiro[indole-3,2'-pyrrole] (4c). Yield 291 mg (68%), yellow oil. ^{1}H NMR ($CDCl_3$, 400 MHz): δ = 8.24–8.00 (m, 4H), 7.60–7.39 (m, 7H), 7.19–7.15 (m, 1H), 7.07–7.00 (m, 2H), 6.78–6.74 (m, 2H), 6.64–6.46 (m, 2H), 4.20 (dd, *J* = 11.6, 8.8 Hz, 1H), 3.78 (dd, *J* = 16.8, 11.6 Hz, 1H), 3.65–3.57 (m, 4H) ppm; ^{13}C{^{1}H} NMR ($CDCl_3$, 100 MHz): δ = 179.4, 176.4, 158.5, 153.9, 138.4, 134.4, 133.9, 131.6, 130.9, 129.3, 129.0 (2C), 128.9 (2C), 128.7 (2C), 128.4 (2C), 128.2 (2C), 126.6, 125.5, 123.3, 121.1, 113.4 (2C), 94.5, 55.2, 52.2, 40.1 ppm. HRMS (ESI$^+$) m/z: [M+H]$^+$ Calcd for $C_{30}H_{25}N_2O^+$ 429.1961; found 429.1958.

2,5'-Diphenyl-3'-[4-(trifluoromethyl)phenyl]-3',4'-dihydrospiro[indole-3,2'-pyrrole] (4d). Yield 354 mg (76%), yellow oil. ^{1}H NMR ($CDCl_3$, 400 MHz): δ = 8.20–8.15 (m, 4H), 7.65–7.54 (m, 6H), 7.48–7.46 (m, 1H), 7.34–7.32 (m, 2H), 7.26–7.23 (m, 1H), 7.09–7.08 (m, 2H), 7.02–7.00 (m, 2H), 4.34 (dd, *J* = 11.6, 8.6 Hz, 1H), 3.91 (dd, *J* = 16.8, 11.6 Hz, 1H), 3.73 (dd, *J* = 16.8, 8.6 Hz, 1H) ppm; ^{13}C{^{1}H} NMR ($CDCl_3$, 100 MHz): δ = 178.9, 176.1, 153.7, 140.1 (br s), 137.8, 134.1, 133.6, 131.8, 131.1, 129.6, 129.5 (q, J_{CF} = 31.0 Hz), 129.1 (2C), 129.0 (2C), 128.7 (2C), 128.3 (2C), 127.7 (2C), 126.8 (q, J_{CF} = 275.0 Hz), 125.7, 124.9 (q, J_{CF} = 3.9 Hz, 2C), 123.1, 121.3, 94.4, 52.4, 39.6 ppm; ^{19}F NMR (376 MHz, $CDCl_3$) δ = - 67.43 (s) ppm. HRMS (ESI$^+$) m/z: [M+H]$^+$ Calcd for $C_{30}H_{22}F_3N_2^+$ 467.1730; found 467.1726.

2,3'-Diphenyl-5'-(*p*-tolyl)-3',4'-dihydrospiro[indole-3,2'-pyrrole] (4e). Yield 276 mg (67%), yellow oil. ^{1}H NMR ($CDCl_3$, 400 MHz): δ = 8.17–8.14 (m, 2H), 8.01–7.99 (m, 2H), 7.51–7.46 (m, 3H), 7.42–7.40 (m, 1H), 7.36–7.34 (m, 2H), 7.18–7.14 (m, 1H), 7.06–6.99 (m, 5H), 6.87–6.85 (m, 2H), 4.25 (dd, *J* = 11.6, 8.8 Hz, 1H), 3.84 (dd, *J* = 16.8, 11.6 Hz, 1H), 3.62 (dd, *J* = 16.8, 8.8 Hz, 1H), 2.47 (s, 3H) ppm; ^{13}C{^{1}H} NMR ($CDCl_3$, 100 MHz): δ = 179.3, 176.3, 153.6, 142.1, 138.3, 135.9, 133.7, 131.5, 130.9, 129.6 (2C), 129.2, 128.9 (2C), 128.7 (2C), 128.2 (2C), 127.9 (2C), 127.3 (2C), 127.0, 125.5, 123.3, 121.0, 94.4, 52.5, 39.6, 21.7 ppm. HRMS (ESI$^+$) m/z: [M+H]$^+$ Calcd for $C_{30}H_{25}N_2^+$ 413.2012; found 413.2012.

5'-(4-Methoxyphenyl)-2,3'-diphenyl-3',4'-dihydrospiro[indole-3,2'-pyrrole] (4f). Yield 321 mg (75%), yellow oil. ^{1}H NMR ($CDCl_3$, 400 MHz): δ = 8.21–8.10 (m, 2H), 8.07–8.00 (m, 2H), 7.52–7.44 (m, 3H), 7.43–7.35 (m, 1H), 7.18–7.13 (m, 1H), 7.07–6.97 (m, 7H), 6.88–6.82 (m, 2H), 4.23 (dd, *J* = 11.6, 8.8 Hz, 1H), 3.91 (s, 3H), 3.81 (dd, *J* = 16.8, 11.6 Hz, 1H), 3.60 (dd, *J* = 16.8, 8.8 Hz, 1H) ppm; ^{13}C{^{1}H} NMR ($CDCl_3$, 100 MHz): δ = 179.5, 175.6, 162.5, 153.8, 138.6, 136.1, 133.9, 130.9, 130.0 (2C), 129.1, 128.9 (2C), 128.7 (2C), 127.9 (2C), 127.3 (2C), 127.2, 127.0, 125.5, 123.4, 121.0, 114.3 (2C), 94.5, 55.6, 52.6, 39.6 ppm. HRMS (ESI$^+$) m/z: [M+H]$^+$ Calcd for $C_{30}H_{25}N_2O^+$ 429.1961; found 429.1964.

5'-(3,4-Dichlorophenyl)-2,3'-diphenyl-3',4'-dihydrospiro[indole-3,2'-pyrrole] (4g). Yield 308 mg (66%), red oil, ^{1}H NMR ($CDCl_3$, 400 MHz): δ = 8.10 (d, *J* = 2.0 Hz, 1H), 8.02–7.98 (m, 2H), 7.81 (dd, *J* = 8.4, 2.0 Hz, 1H), 7.53–7.50 (m, 1H), 7.42–7.38 (m, 2H), 7.33–7.31 (m, 1H), 7.15–7.07 (m, 3H), 6.93–6.90 (m, 4H), 6.76–6.73 (m, 2H), 4.18 (dd, *J* = 11.6, 8.8 Hz, 1H), 3.71 (dd, *J* = 16.8, 11.6 Hz, 1H), 3.48 (dd, *J* = 16.8, 8.8 Hz, 1H) ppm; ^{13}C{^{1}H} NMR ($CDCl_3$, 100 MHz): δ = 178.9, 174.4, 153.8, 137.8, 136.0, 135.6, 134.1, 133.64, 133.56, 131.1, 131.0, 130.0, 129.5, 129.0 (2C), 128.6 (2C), 128.0 (2C), 127.3 (3C), 127.2, 125.6, 123.2, 121.2, 94.6, 52.5, 39.5 ppm. HRMS (ESI$^+$) m/z: [M+H]$^+$ Calcd for $C_{29}H_{21}Cl_2N_2^+$ 467.1076; found 467.1073.

3',5'-Diphenyl-2-(*p*-tolyl)-3',4'-dihydrospiro[indole-3,2'-pyrrole] (4h). Yield 330 mg (80%), yellow oil. ^{1}H NMR ($CDCl_3$, 400 MHz): δ = 8.03–7.89 (m, 4H), 7.45–7.40 (m, 3H), 7.19–7.14 (m, 2H), 7.07–7.00 (m, 2H), 6.94–6.85 (m, 5H), 6.77–6.72 (m, 2H), 4.15 (dd, *J* = 11.6, 8.8 Hz, 1H),

3.74 (dd, J = 16.8, 11.6 Hz, 1H), 3.52 (dd, J = 16.8, 8.8 Hz, 1H), 2.31 (s, 3H) ppm; $^{13}C\{^1H\}$ NMR ($CDCl_3$, 100 MHz): δ = 179.2, 176.2, 154.0, 141.3, 138.3, 136.0, 134.4, 131.6, 131.1, 129.7 (2C), 129.2, 128.9 (2C), 128.7 (2C), 128.2 (2C), 127.9 (2C), 127.4 (2C), 127.0, 125.3, 123.3, 120.9, 94.5, 52.8, 39.7, 21.7 ppm. HRMS (ESI$^+$) m/z: [M+H]$^+$ Calcd for $C_{30}H_{25}N_2^+$ 413.2012; found 413.2012.

2-(4-Chlorophenyl)-3',5'-diphenyl-3',4'-dihydrospiro[indole-3,2'-pyrrole] (4i). Yield 233 mg (54%), red oil, ^1H NMR ($CDCl_3$, 400 MHz): δ = 8.11–8.08 (m, 4H), 7.59–7.52 (m, 3H), 7.47–7.45 (m, 2H), 7.41–7.39 (m, 1H), 7.19–7.15 (m, 1H), 7.07–7.00 (m, 5H), 6.84–6.82 (m, 2H), 4.21 (dd, J = 11.6, 8.8 Hz, 1H), 3.86 (dd, J = 16.8, 11.6 Hz, 1H), 3.65 (dd, J = 16.8, 8.8 Hz, 1H) ppm; $^{13}C\{^1H\}$ NMR ($CDCl_3$, 100 MHz,) δ = 178.0, 176.8, 153.4, 138.1, 137.2, 135.5, 134.1, 132.1, 131.8, 130.0 (2C), 129.4, 129.3 (2C), 129.0 (2C), 128.2 (2C), 128.0 (2C), 127.3 (2C), 127.1, 125.8, 123.3, 121.1, 94.3, 52.8, 39.6 ppm. HRMS (ESI$^+$) m/z: [M+H]$^+$ Calcd for $C_{29}H_{22}ClN_2^+$ 433.1466; found 433.1462.

2-(4-Methoxyphenyl)-3',5'-diphenyl-3',4'-dihydrospiro[indole-3,2'-pyrrole] (4j). Yield 312 mg (73%), red oil, ^1H NMR ($CDCl_3$, 400 MHz): δ = 8.13–8.09 (m, 4H), 7.57–7.51 (m, 3H), 7.37–7.35 (m, 1H), 7.16–7.12 (m, 1H), 7.06–6.96 (m, 7H), 6.87–6.85 (m, 2H), 4.27 (dd, J = 11.6, 8.8 Hz, 1H), 3.89–3.82 (m, 4H), 3.64 (dd, J = 16,8, 8.8 Hz, 1H) ppm; $^{13}C\{^1H\}$ NMR ($CDCl_3$, 100 MHz): δ = 178.7, 176.2, 162.0, 154.0, 138.1, 136.0, 134.4, 131.6, 130.5 (2C), 129.2, 128.9 (2C), 128.2 (2C), 127.9 (2C), 127.3 (2C), 127.0, 126.5, 125.0, 123.2, 120.6, 114.4 (2C), 94.4, 55.5, 53.2, 39.6 ppm. HRMS (ESI$^+$) m/z: [M+H]$^+$ Calcd for $C_{30}H_{25}N_2O^+$ 429.1961; found 429.1963.

4-(3',5'-Diphenyl-3',4'-dihydrospiro[indole-3,2'-pyrrol]-2-yl)phenol (4k). Yield 356 mg (86%), red oil, ^1H NMR (DMSO-d_6, 400 MHz): δ = 10.17 (br s, 1H), 8.06–8.01 (m, 2H), 7.98–7.91 (m, 2H), 7.59–7.52 (m, 3H), 7.25–7.12 (m, 3H), 6.99–6.85 (m, 8H), 4.25 (dd, J = 11.6, 8.6 Hz, 1H), 4.07 (dd, J = 16.8, 11.6 Hz, 1H), 3.74 (dd, J =16.8, 8.6 Hz, 1H) ppm; $^{13}C\{^1H\}$ NMR (DMSO-d_6, 100 MHz,): δ = 178.5, 176.7, 160.2, 153.4, 138.3, 136.4, 134.1, 131.3, 130.2 (2C), 128.81, 128.79 (2C), 128.0 (2C), 127.7 (2C), 127.1 (2C), 126.6, 124.8, 124.1, 123.7, 119.6, 115.8 (2C), 93.4, 52.7, 38.9 ppm. HRMS (ESI$^+$) m/z: [M+H]$^+$ Calcd for $C_{29}H_{23}N_2O^+$ 415.1805; found 415.1798.

5'-(Naphthalen-2-yl)-3'-phenyl-2-(*p*-tolyl)-3',4'-dihydrospiro[indole-3,2'-pyrrole] (4n). Yield 305 mg (66%), yellow oil, ^1H NMR ($CDCl_3$, 400 MHz): δ = 8.35 (br s, 1H), 8.22–8.19 (m, 1H), 7.99–7.96 (m, 1H), 7.89–7.82 (m, 3H), 7.51–7.47 (m, 2H), 7.32–7.28 (m, 1H), 7.20–7.15 (m, 2H), 7.13–7.01 (m, 3H), 6.95–6.89 (m, 4H), 6.82–6.78 (m, 2H), 4.22 (dd, J = 11.6, 8.8 Hz, 1H), 3.87 (dd, J = 16.8, 11.6 Hz, 1H), 3.67 (dd, J = 16.8, 8.8 Hz, 1H), 2.32 (s, 3H) ppm; $^{13}C\{^1H\}$ NMR ($CDCl_3$, 100 MHz): δ = 179.3, 176.3, 154.0, 141.3, 138.3, 136.1, 135.1, 133.2, 131.8, 131.1, 129.7 (2C), 129.2, 129.1, 129.0, 128.7 (2C), 128.1, 127.9 (2C), 127.8, 127.4 (2C), 127.0, 126.9, 126.7, 125.3, 124.8, 123.3, 120.9, 94.6, 52.8, 39.7, 21.7 ppm. HRMS (ESI$^+$) m/z: [M+H]$^+$ Calcd for $C_{34}H_{27}N_2^+$ 463.2169; found 463.2173.

5'-Methyl-3'-phenyl-2-(*p*-tolyl)-3',4'-dihydrospiro[indole-3,2'-pyrrole] (4o). Yield 70 mg (20%), yellow oil, ^1H NMR (DMSO-d_6, 400 MHz): δ = 7.99–7.97 (m, 2H), 7.38–7.36 (m, 2H), 7.28–7.22 (m, 2H), 7.15–7.11 (m, 1H), 7.03–6.92 (m, 4H), 6.76–6.74 (m, 2H), 4.08 (dd, J = 11.8, 8.6 Hz, 1H), 3.6 7 (dd, J = 17.2, 11.8 Hz, 1H), 3.13 (dd, J = 17.2, 8.6 Hz, 1H), 2.41 (s, 3H), 2.32 (s, 3H); $^{13}C\{^1H\}$ NMR (DMSO-d_6, 100 MHz,): δ = 179.5, 178.4, 152.9, 140.8, 138.6, 136.3, 130.3, 129.3 (2C), 128.5, 128.0 (2C), 127.4 (2C), 126.8 (2C), 126.3, 124.9, 123.5, 119.7, 93.3, 52.5, 42.4, 21.0, 20.1 ppm. HRMS (ESI$^+$) m/z: [M+H]$^+$ Calcd for $C_{25}H_{23}N_2^+$ 351.1856; found 351.1861.

Synthesis of 3',4'-dihydrospiro[indole-3,2'-pyrrole] 4a in 4.2 mmol scale

To a stirred solution of indole **3a** (1.684 g, 4.2 mmol) and $NaOAc \cdot 3H_2O$ (685 mg, 5.04 mmol, 1.2 eq.) in MeOH (42 mL) was added $NH_2OH \cdot HCl$ (350 mg, 5.04 mmol, 1.2 eq.), and the reaction mixture was refluxed until full conversion of the starting material by TLC was observed (12 h). The resulting oil was dissolved in 1,4-dioxane (80 mL), Ac_2O (595 µl, 1.5 eq.) was added in a single portion at room temperature, and the reaction mixture was stirred for 30 min (TLC control). The reaction mixture was poured into water (300 mL), and the product was extracted with ethyl acetate (3 × 100 mL). Combined organic fractions were washed with water (2 × 100 mL) and dried over anhydrous Na_2SO_4. After filtration and evaporation of ethyl acetate. The residue was dissolved in CH_3CN (21 mL) and $FeCl_2$ (106.5 mg, 0.84 mmol) was added, and reaction mixture was stirred at room temperature for 30 min (TLC control). Isolation by column chromatography on silica gel (petroleum ether/ethyl acetate = 3:1, v/v) gave 922 mg (55%) of 2,3',5'-Triphenyl-3',4'-dihydrospiro[indole-3,2'-pyrrole] (**4a**).

3. Antimicrobial activity

General procedure for antibacterial and antifungal activity

The synthesized compounds **4** were tested for their in vitro growth inhibitory and bactericidal (fungicidal) activity against *Candida albicans* ATCC 10231, *C. albicans* C1 (clinical strain), *Staphylococcus aureus* ATCC 25923, *S. aureus* ATCC 43300 (MRSA), *Mycobacterium smegmatis* ATCC 70084, *Escherichia coli* ATCC 25922, *E. coli* ATCC 8739, and *Klebsiella pneumoniae* ATCC 700603 in a manner similar to the described procedure.[3] Standard antibiotics served as positive controls: Fluconazole for *C. albicans*, amikacin for *E. coli, K. pneumoniae* and *S. aureus*, isoniazid for *M. smegmatis*. The cells were grown overnight at 37 °C in a glass tube with 5 mL of LB broth containing 1% of glucose for *C. albicans* and 0.05% Tween 80 for *M. smegmatis*. The grown cultures were then diluted 1:100 with fresh medium and cultivated for 5 h. The *M. smegmatis* culture was grown for 20 h with agitation on a shaker GFL1092 (GFL, Germany) (200 rpm, 37 °C). Then, the cultures were adjusted to OD 0.1 (A625) and diluted to 1:10 for *M. smegmatis* and 1:100 for the other microorganisms. The resulting suspension was used to determine the MIC using the serial dilutions method in 96-well plates with modifications. The synthesized compounds were dissolved in DMSO at a concentration of 20 mg/mL and a series of two-fold dilutions was prepared in the same solvent. Then, 10 µL of the solutions was added to the wells of the plate containing 190 µL of the cell suspension. To the control wells, 10 µL of DMSO was added. The plates were incubated at 37 °C under static conditions for 24 h (72 h for *M. smegmatis*) for the minimum inhibitory concentration determination. The minimum bactericidal concentration and minimum fungicidal concentration were determined as the lowest concentration of an antimicrobial agent required to achieve a 99.9% reduction of colony forming units (CFU) number in the initial inoculum. In total, 10 µL from each well of a plate for the MIC determination was inoculated on Petri dishes with LB agar (containing 1% of glucose for *C. albicans*), and colonies were checked after incubation (24 h, 37 °C) to determine the MBC or MFC.

Materials

The reagents for the analysis of the antimicrobial activity: Amikacin sulfate—Sigma-Aldrich (St. Louis, MO, USA), Fluconazole—Sigma-Aldrich (St. Louis, MO, USA), Isoniazid—Sigma (St. Louis, MO, USA), LB-broth—VWR (Radnor, PA, USA), and LB-agar—Sigma (St. Louis, MO, USA).

Table S1: Antimicrobial data (MIC and MBC/MFC, µg/mL) for the spiro[indoline-3,2'-pyrrolidine] derivatives **4**[a]

	MIC and MBC/MFC, µg/mL												Positive control, µg/mL
	**4a**	**4b**	**4c**	**4d**	**4e**	**4f**	**4g**	**4h**	**4i**	**4j**	**4k**	**4n**	
C.a. 10231 (MIC) [b]	1000	-	250	-	-	1000	-	-	-	-	-	-	1.94 [k]
C.a. 10231 (MFC)	1000	-	500	-	-	1000	-	-	-	-	-	-	7.8 [k]
C.a. C1 (MIC) [c]	1000	-	500	-	-	1000	-	-	-	-	-	-	15.52 [k]
C.a. C1 (MFC)	1000	-	1000	-	-	1000	-	-	-	-	-	-	31.04 [k]
E. c. 25922 (MIC) [d]	- [j]	-	-	-	-	-	-	-	-	-	-	-	19.53 [l]
E. c. 25922 (MBC)	-	-	-	-	-	-	-	-	-	-	-	-	19.53 [l]
E. c. 8739 (MIC) [e]	-	-	-	-	-	-	-	-	-	-	-	-	19.53 [l]
E. c. 8739 (MBC)	-	-	-	-	-	-	-	-	-	-	-	-	19.53 [l]
K. p. (MIC) [f]	-	-	-	-	-	-	-	-	-	-	-	-	19.53 [l]
K. p. (MBC)	-	-	-	-	-	-	-	-	-	-	-	-	78.12 [l]
S. a. 25923 (MIC) [g]	-	-	-	-	-	-	-	-	-	-	-	-	4.88 [l]
S. a. 25923 (MBC)	-	-	-	-	-	-	-	-	-	-	-	-	9.77 [l]
MRSA (MIC) [h]	-	-	-	-	-	-	-	-	-	-	-	-	9.77 [l]
MRSA (MBC)	-	-	-	-	-	-	-	-	-	-	-	-	9.77 [l]
M. s. 70084 (MIC) [i]	-	-	500	-	-	-	-	-	-	-	250	-	4.58 [m]
M. s. 70084 (MBC)	-	-	-	-	-	-	-	-	-	-	1000	-	9.16 [m]

[a]In the table, the Mode values from 3–5 independent experiments are presented. MIC–minimum inhibitory concentration; MBC–minimum bactericidal concentration; MFC–minimum fungicidal concentration; [b]*Candida albicans* ATCC 10231; [c]*Candida albicans* C1 (clinical strain); [d]*Escherichia coli* ATCC 25922; [e]*Escherichia coli* ATCC 8739; [f]*Klebsiella pneumoniae* ATCC 700603; [g]*Staphylococcus aureus* ATCC 25923; [h]*Staphylococcus aureus* ATCC 43300 (MRSA); [i]*Mycobacterium smegmatis* ATCC 70084; [j](-) >1000 µg/mL; [k]fluconazole; [l]amikacin; [m]isoniazid.

4. References

1. Merkushev A.A.; Makarov A.S.; Shpuntov P.M.; Abaev V.T.; Trushkov I.V.; Uchuskin M.G., Oxidative Rearrangement of 2-(2-Aminobenzyl)furans: Synthesis of Functionalized Indoles and Carbazoles. *Eur. J. Org. Chem.* **2021**, *2021*, 1274.

2. Patel, T.; Gaikwad, R.; Jain, K.; Ganesh, R.; Bobde, Y.; Ghosh, B.; Das, K.; Gayen, S., First Report on 3-(3-oxoaryl) Indole Derivatives as Anticancer Agents: Microwave Assisted Synthesis, In Vitro Screening and Molecular Docking Studies. *ChemistrySelect* **2019,** *4* (15), 4478-4482.

3. Mendogralo, E.Y.; Nesterova, L.Y.; Nasibullina, E.R.; Shcherbakov, R.O.; Tkachenko, A.G.; Sidorov, R.Y.; Sukonnikov, M.A.; Skvortsov, D.A.; Uchuskin, M.G. The Synthesis and Biological Evaluation of 2-(1*H*-Indol-3-yl)quinazolin-4(3*H*)-One Derivatives. *Molecules* **2023**, *28*, 5348.

5. Copies of NMR spectra

1,3-Diphenyl-3-(2-phenyl-1*H*-indol-3-yl)propan-1-one (3a).

^{1}H, $CDCl_3$, 400 MHz

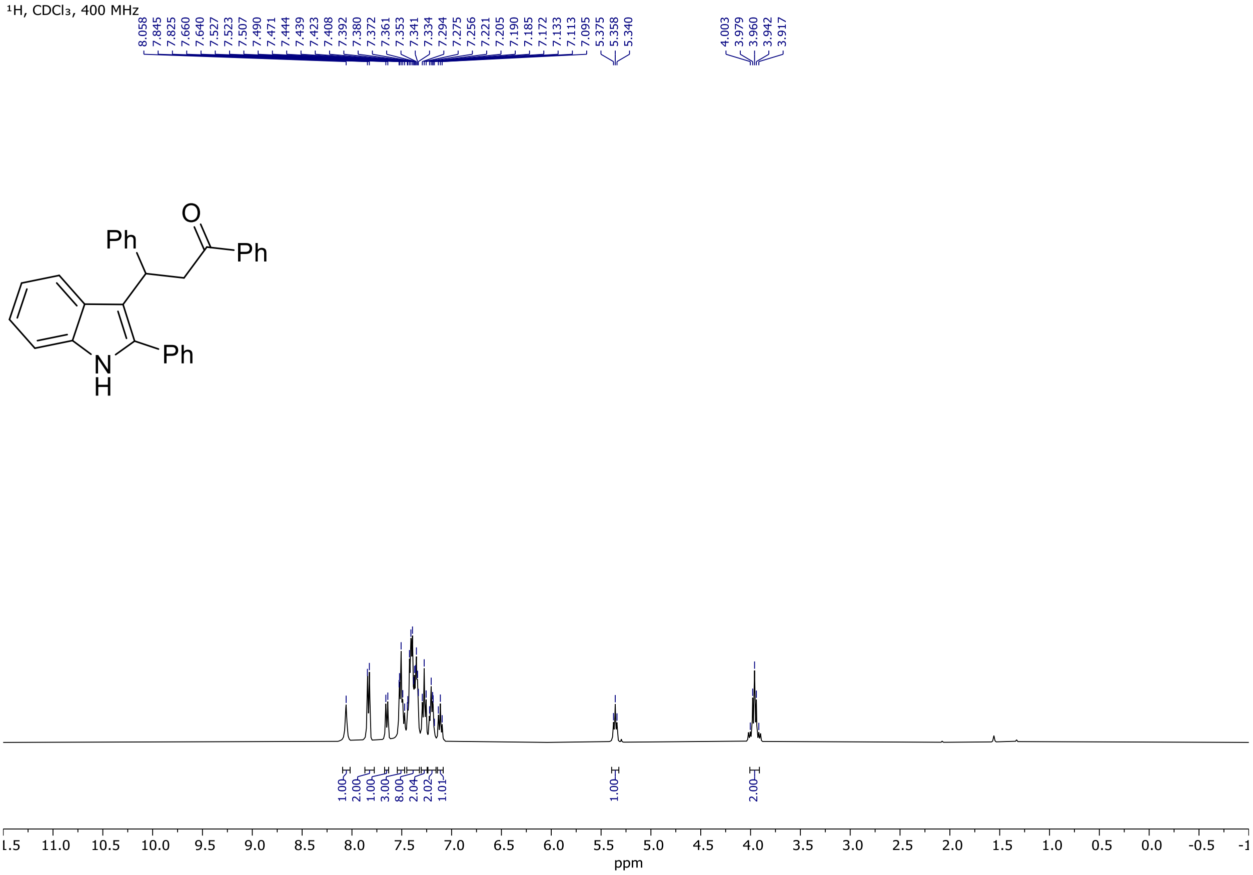

$^{13}C\{^{1}H\}$, $CDCl_3$, 100 MHz

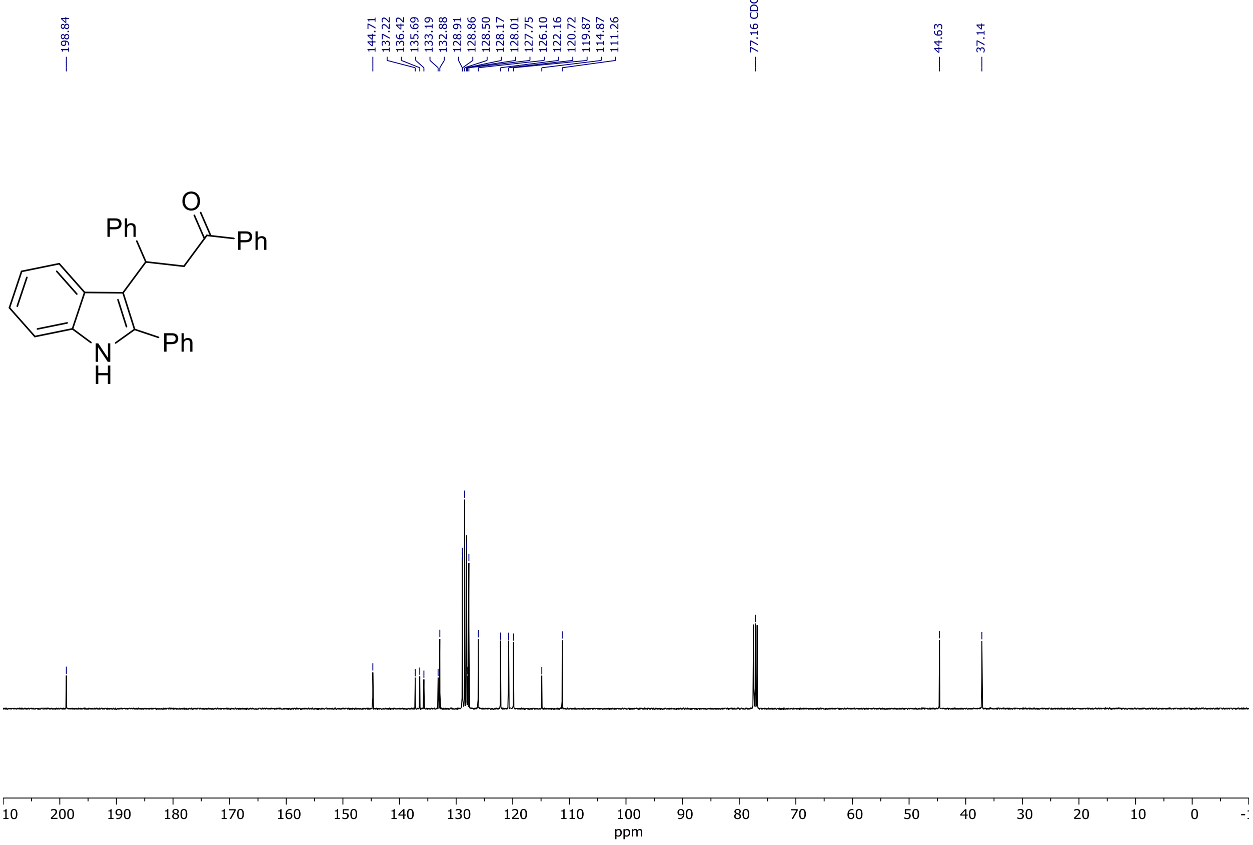

1-Phenyl-3-(2-phenyl-1*H*-indol-3-yl)-3-(*p*-tolyl)propan-1-one (3b).

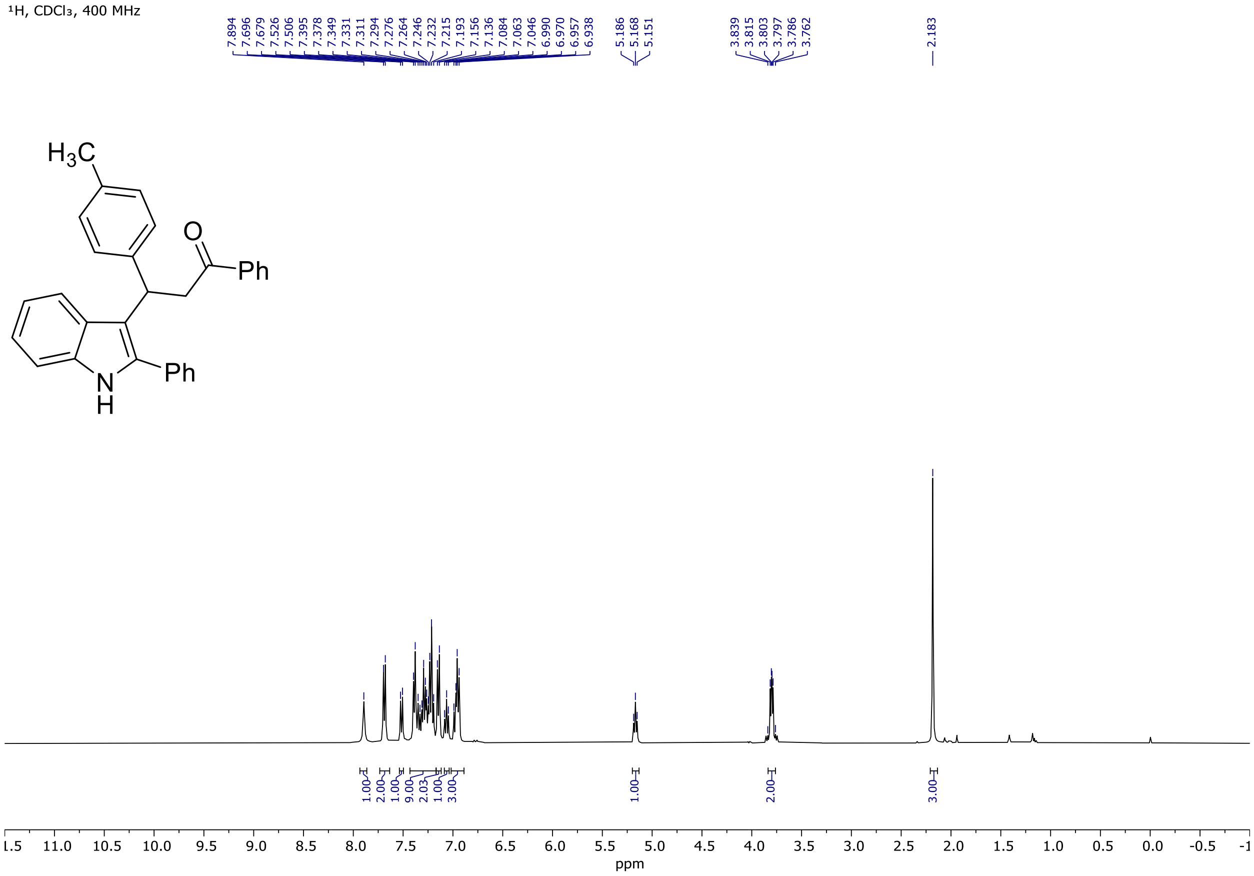

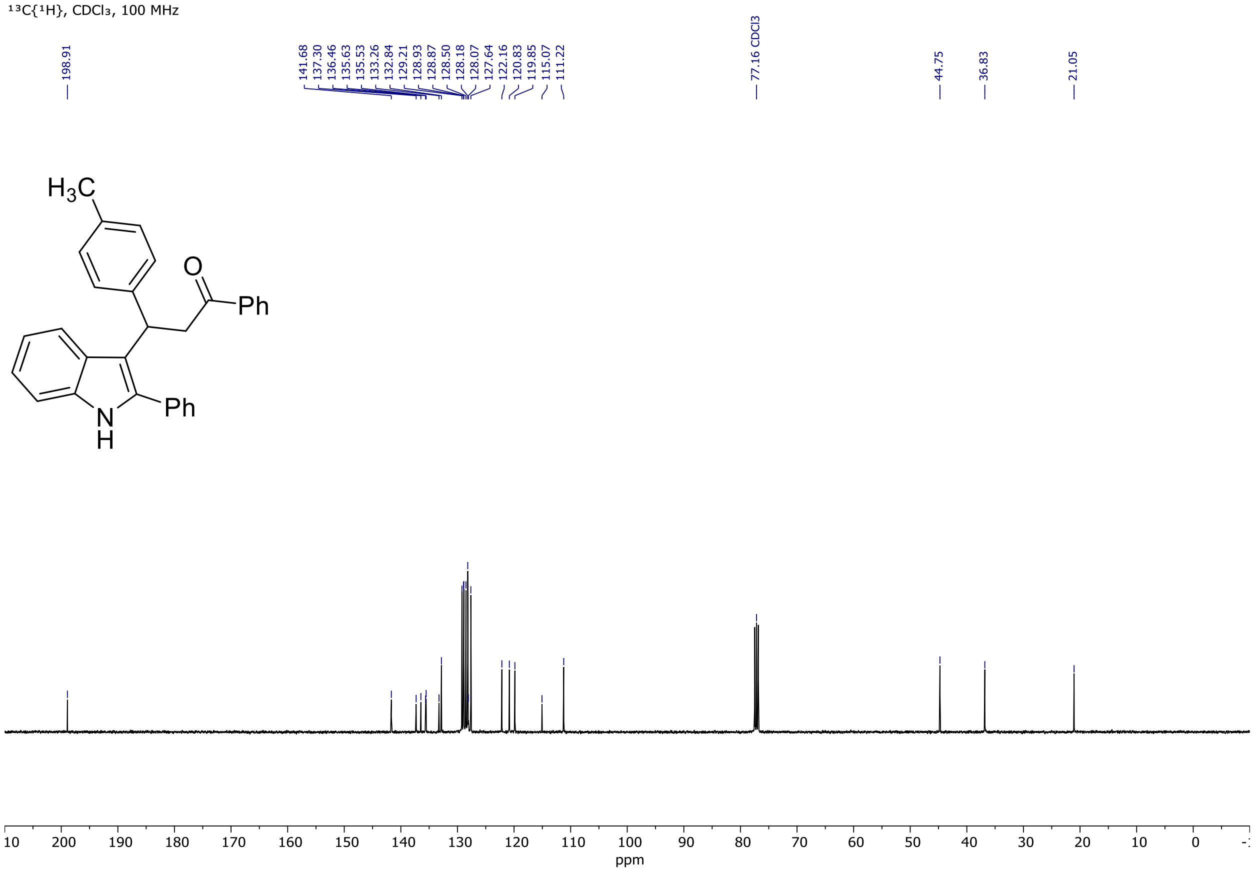

3-(4-Methoxyphenyl)-1-phenyl-3-(2-phenyl-1*H*-indol-3-yl)propan-1-one (3c).

^{1}H, $CDCl_3$, 400 MHz

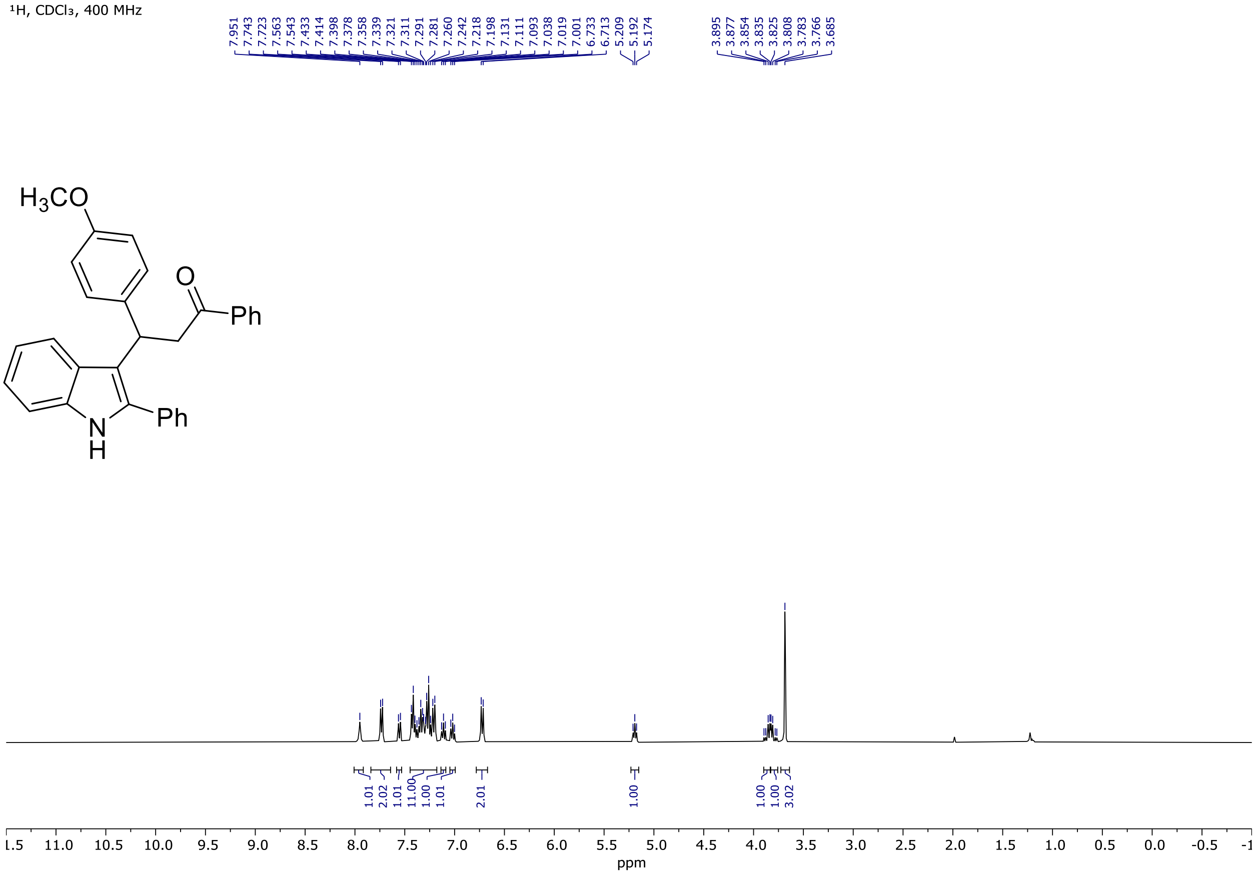

$^{13}C\{^{1}H\}$, $CDCl_3$, 100 MHz

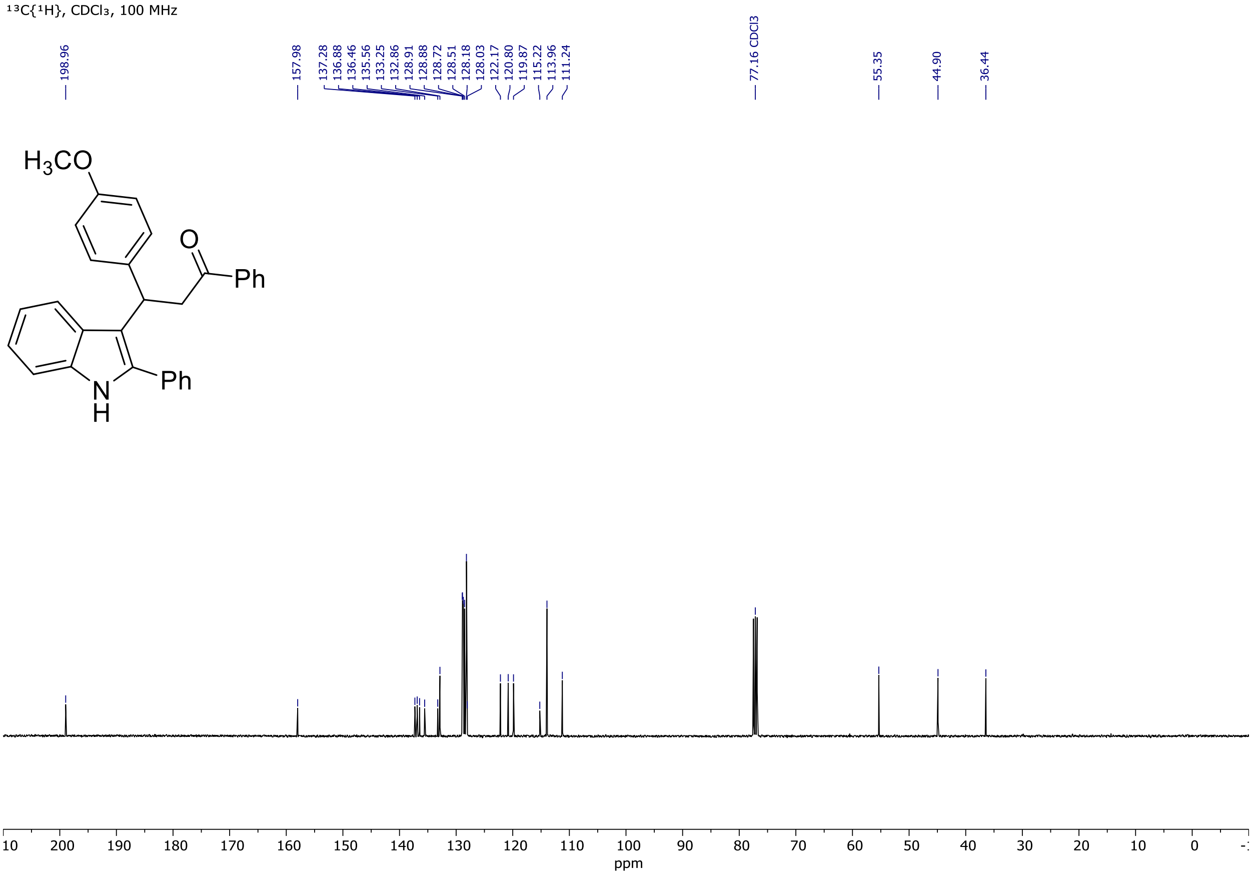

1-Phenyl-3-(2-phenyl-1*H*-indol-3-yl)-3-[4-(trifluoromethyl)phenyl]propan-1-one (3d).

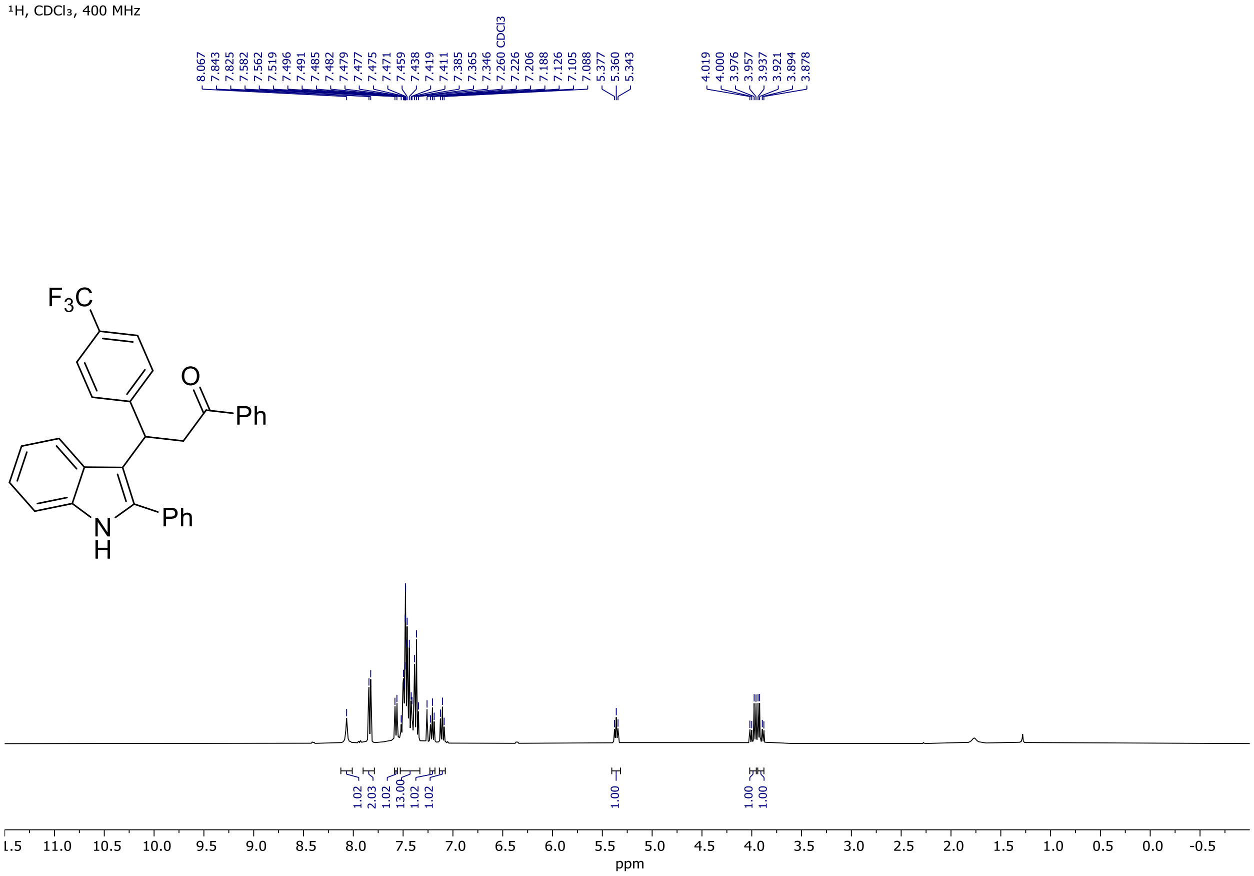

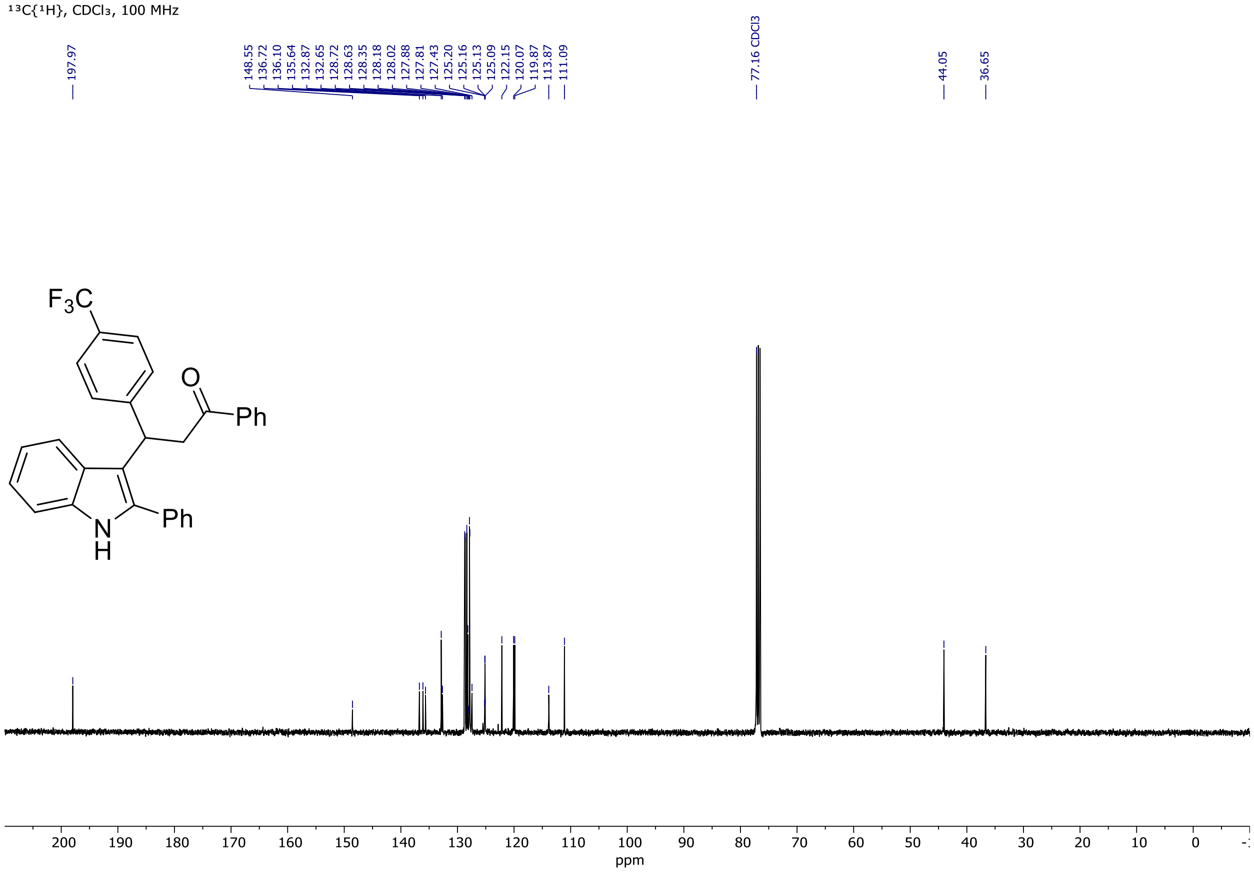

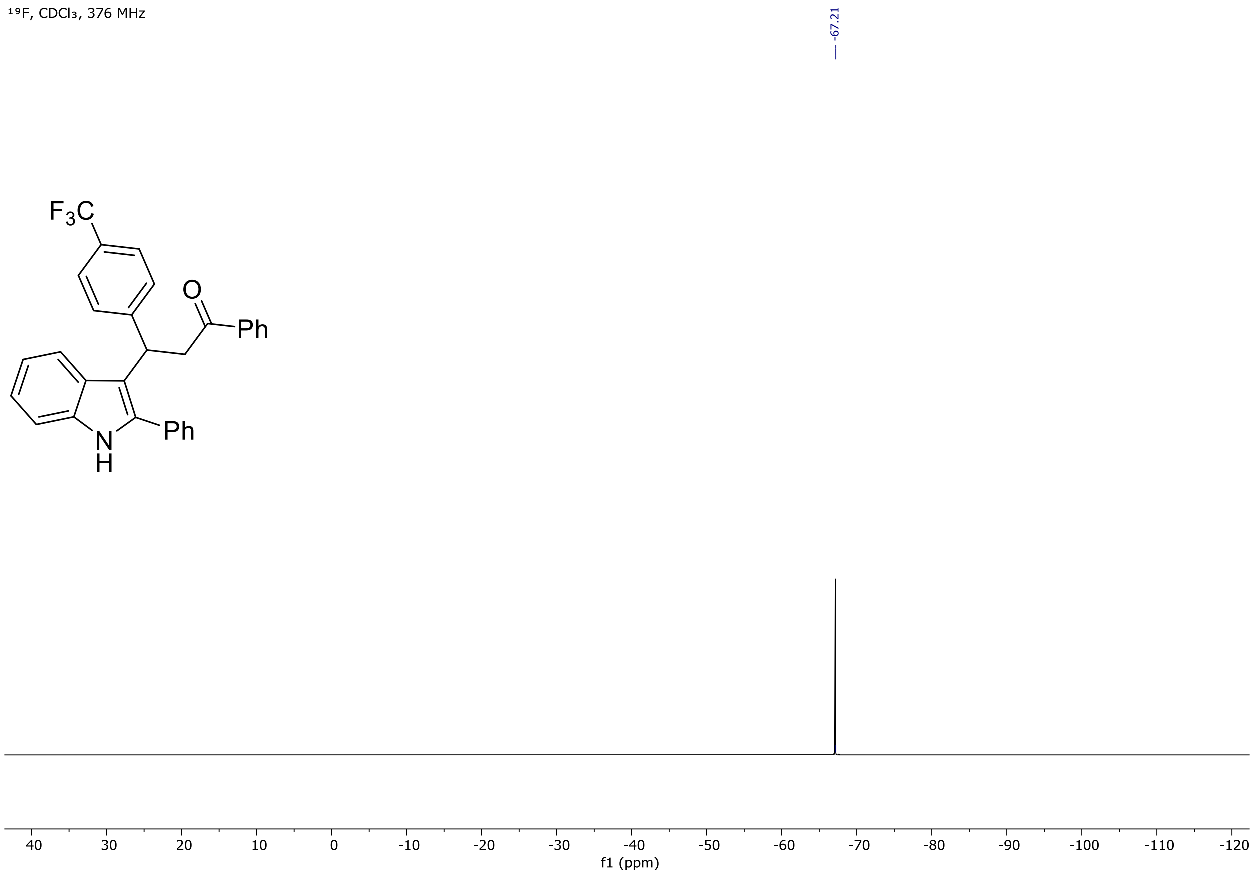
¹⁹F, CDCl₃, 376 MHz
-67.21
F3C
O
Ph
Ph
N
H
40 30 20 10 0 -10 -20 -30 -40 -50 -60 -70 -80 -90 -100 -110 -120
f1 (ppm)

3-Phenyl-3-(2-phenyl-1*H*-indol-3-yl)-1-(*p*-tolyl)propan-1-one (3e).

^{1}H, $CDCl_3$, 400 MHz

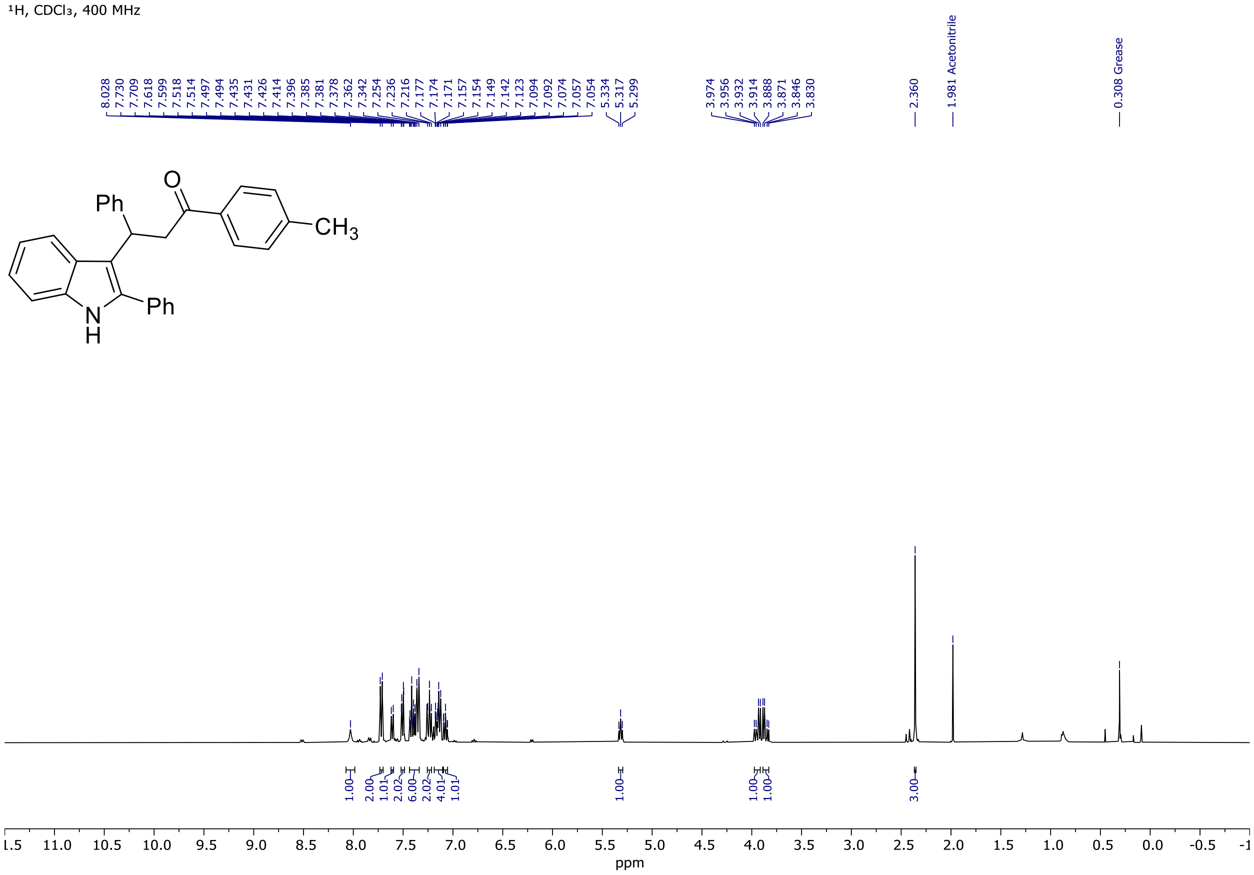

$^{13}C\{^{1}H\}$, $CDCl_3$, 100 MHz

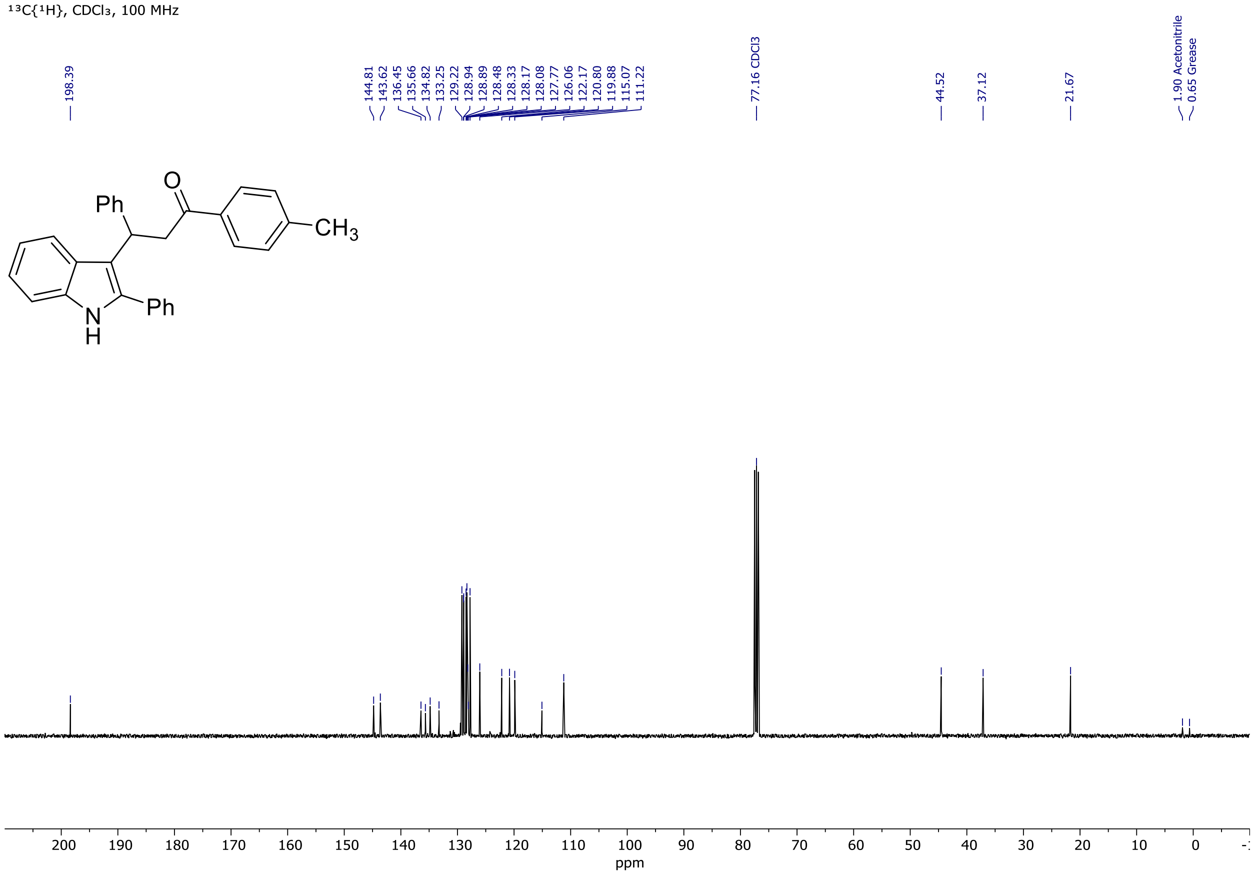

1-(4-Methoxyphenyl)-3-phenyl-3-(2-phenyl-1*H*-indol-3-yl)propan-1-one (3f).

^{1}H, $CDCl_3$, 400 MHz

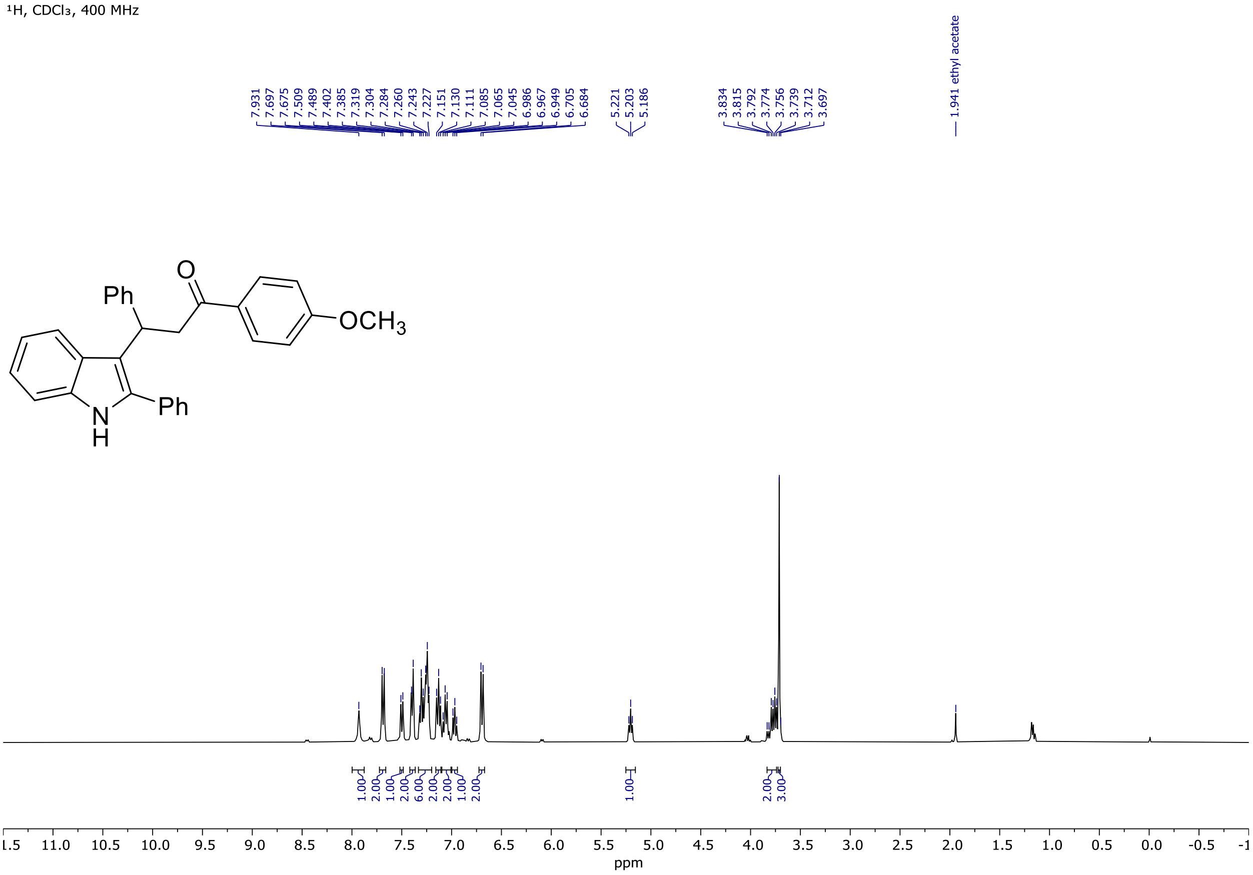

^{13}C{^{1}H}, $CDCl_3$, 100 MHz

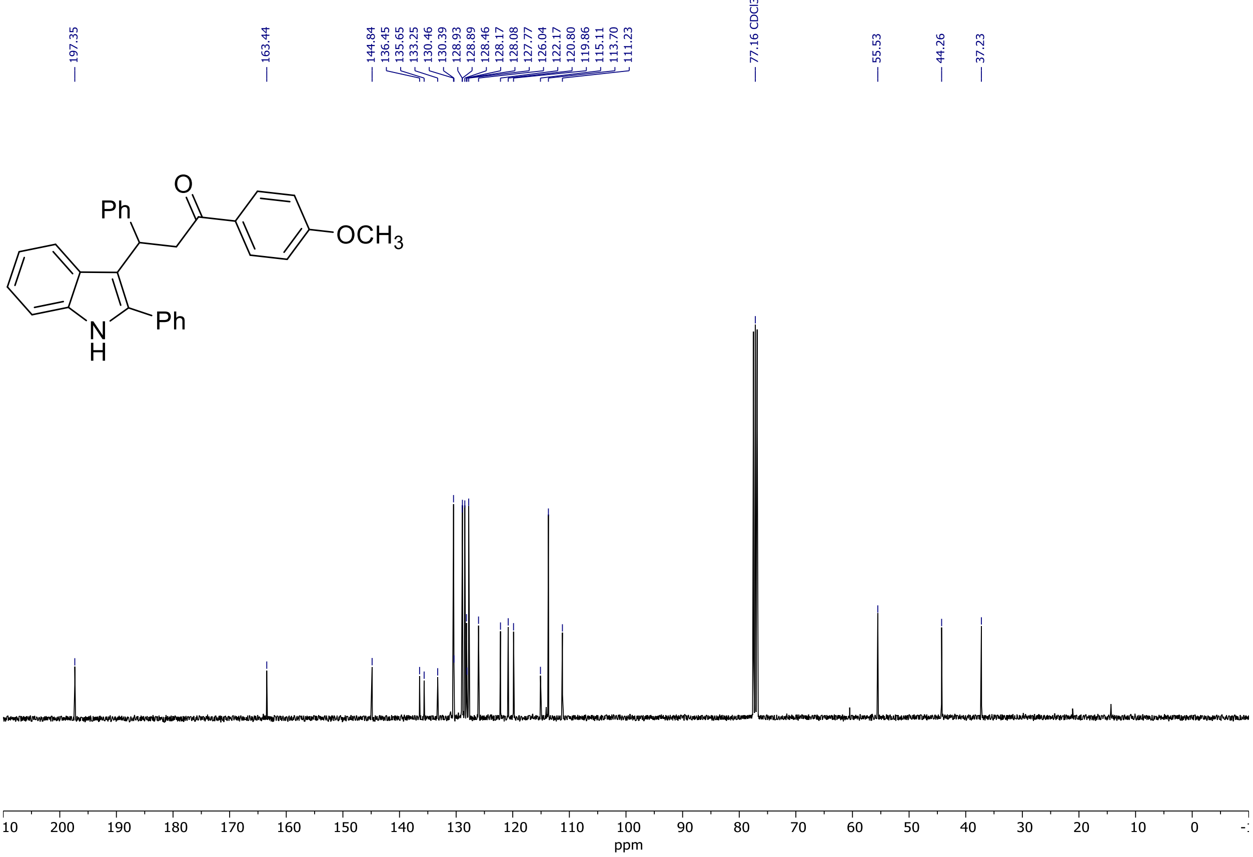

1-(3,4-Dichlorophenyl)-3-phenyl-3-(2-phenyl-1*H*-indol-3-yl)propan-1-one (3g).

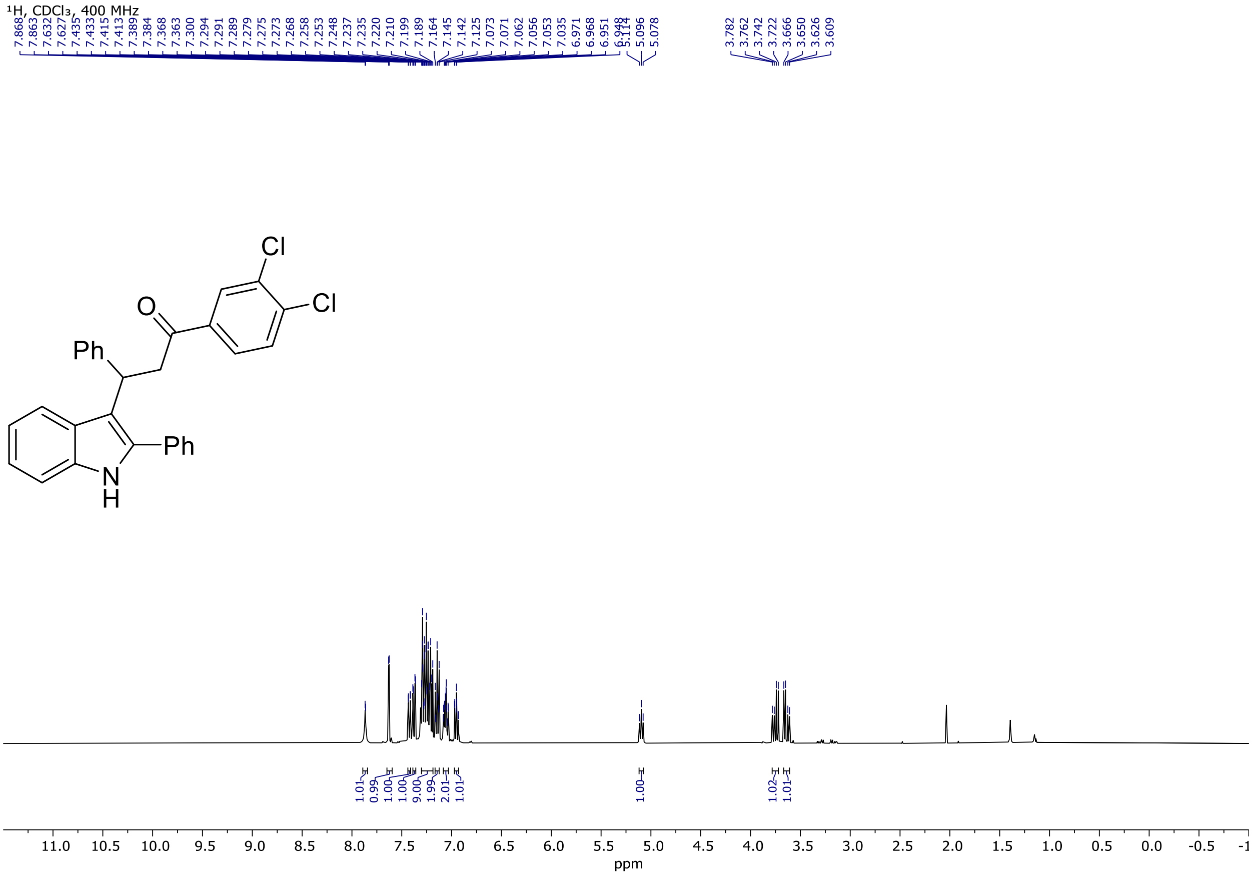

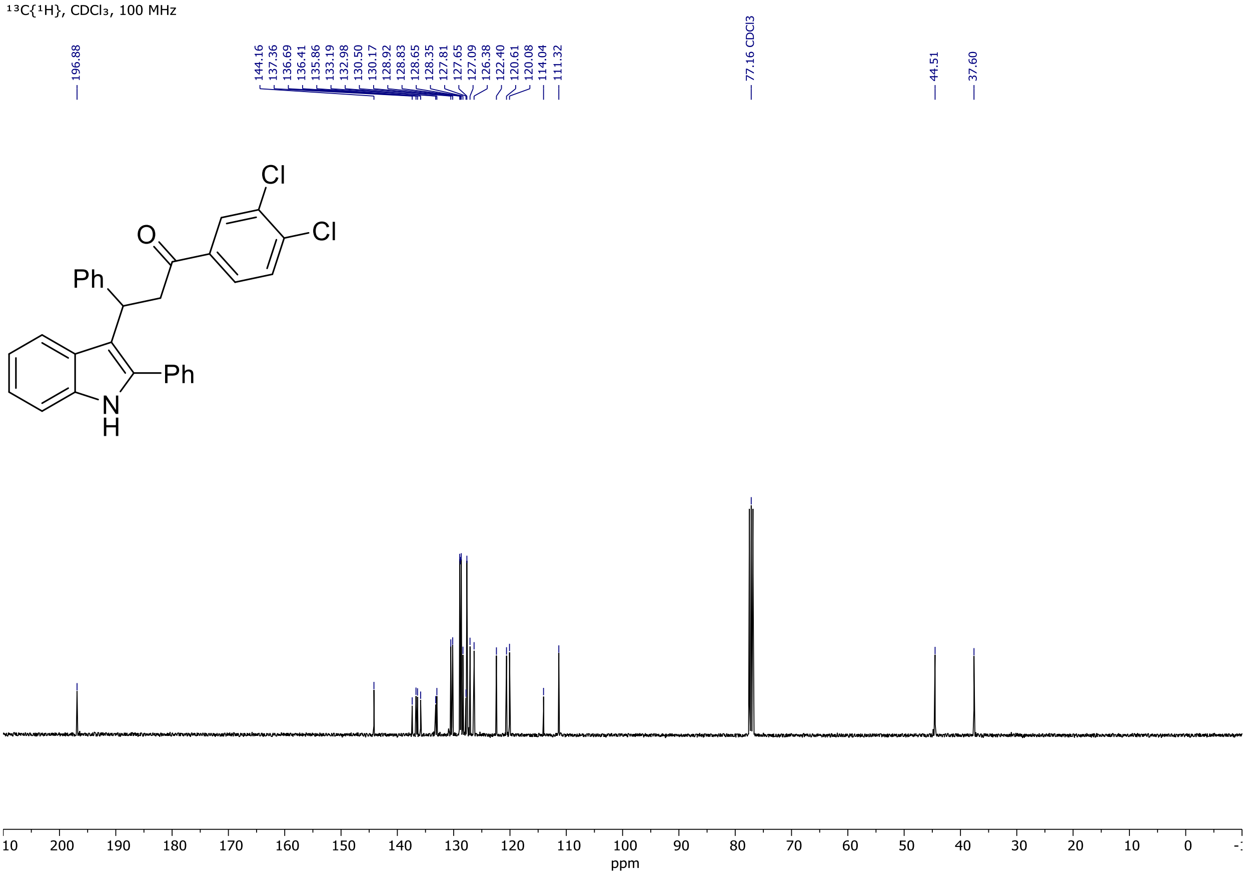

1,3-Diphenyl-3-[2-(*p*-tolyl)-1*H*-indol-3-yl]propan-1-one (3h).

^{1}H, CDCl$_3$, 400 MHz

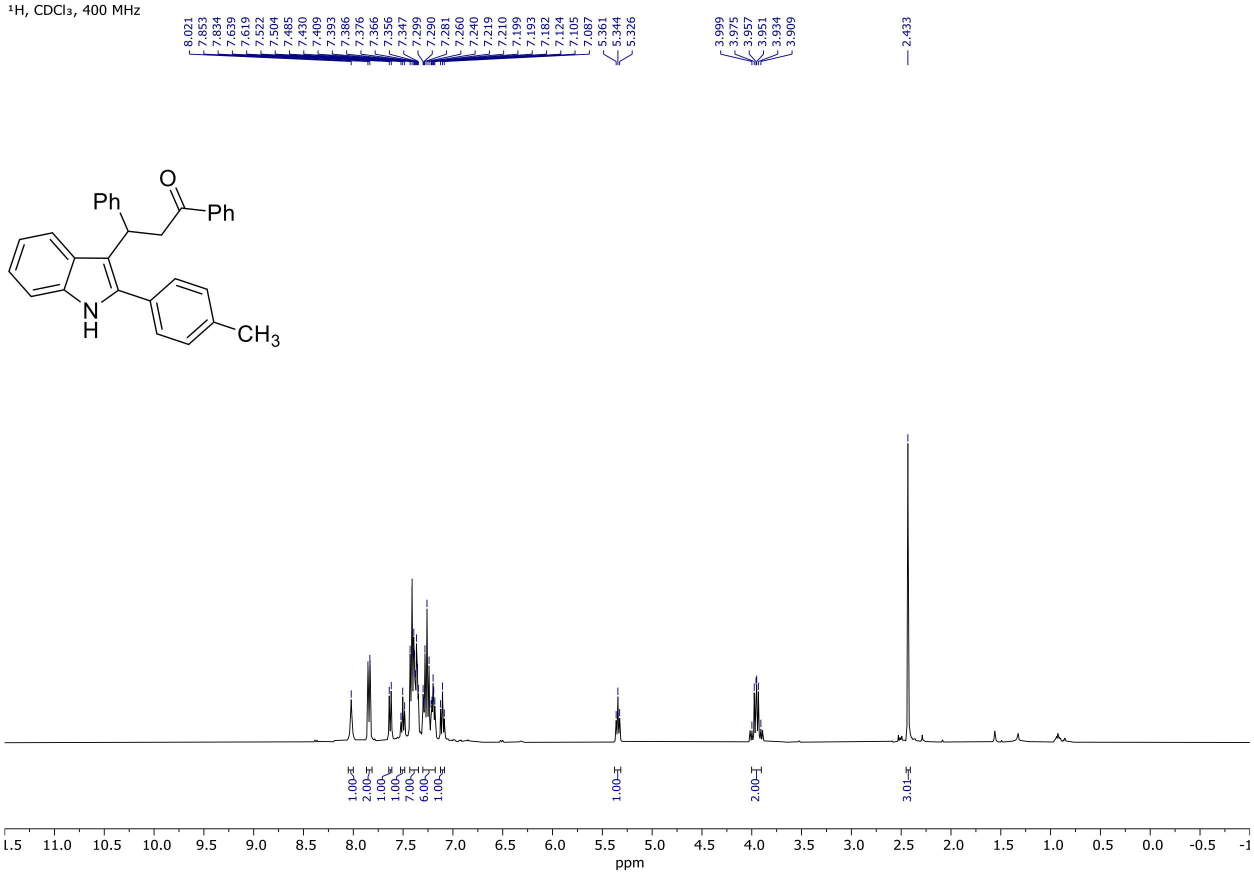

^{13}C{^{1}H}, CDCl$_3$, 100 MHz

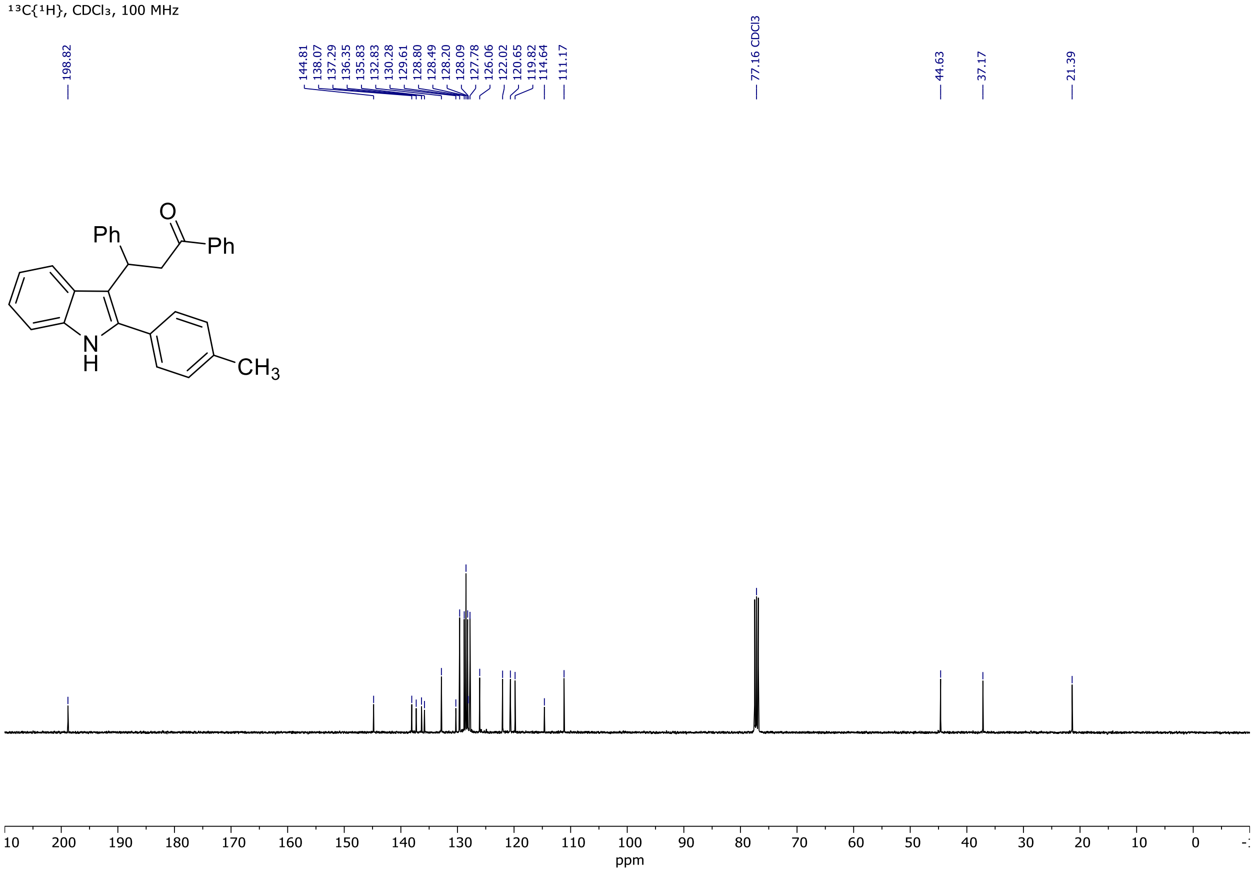

3-[2-(4-Chlorophenyl)-1*H*-indol-3-yl]-1,3-diphenylpropan-1-one (3i).

^{1}H, $CDCl_3$, 400 MHz

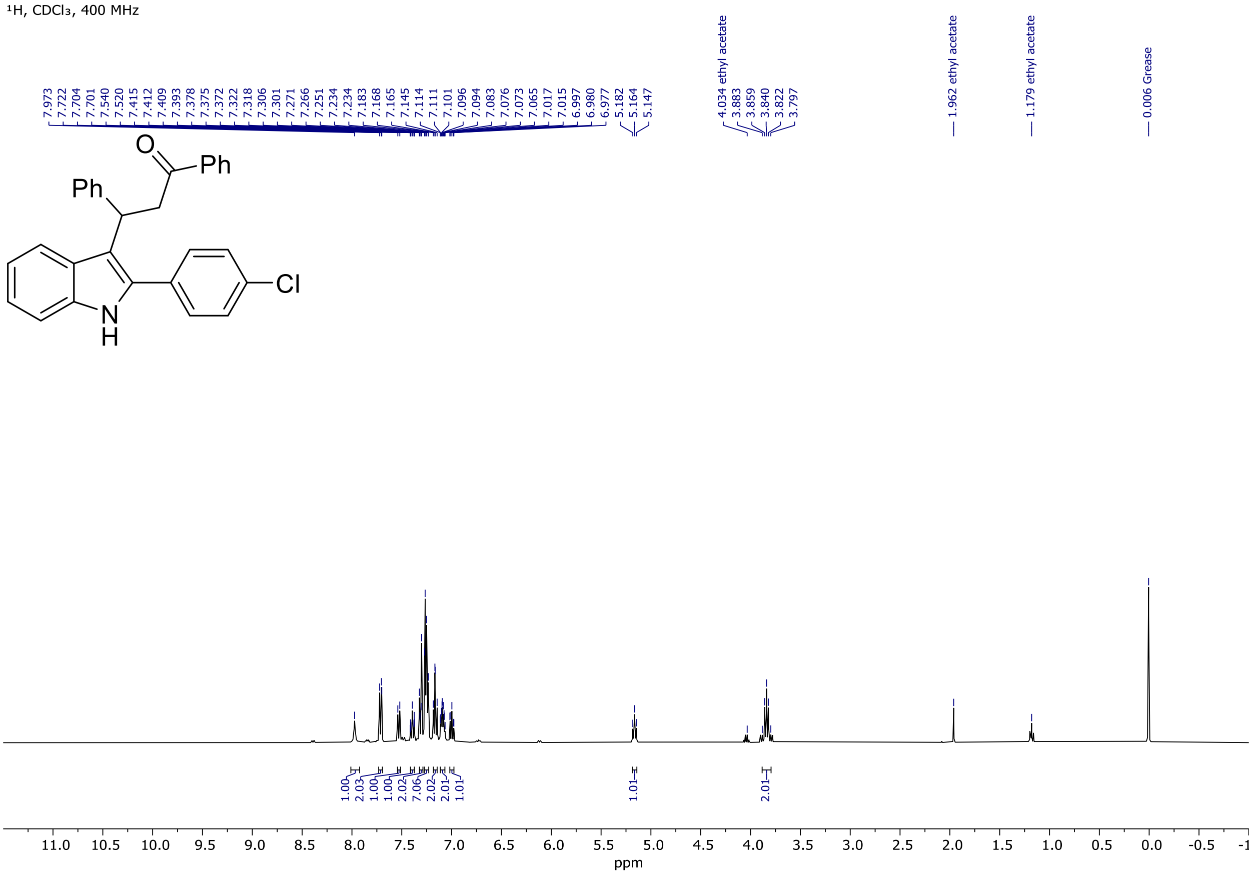

$^{13}C\{^{1}H\}$, $CDCl_3$, 100 MHz

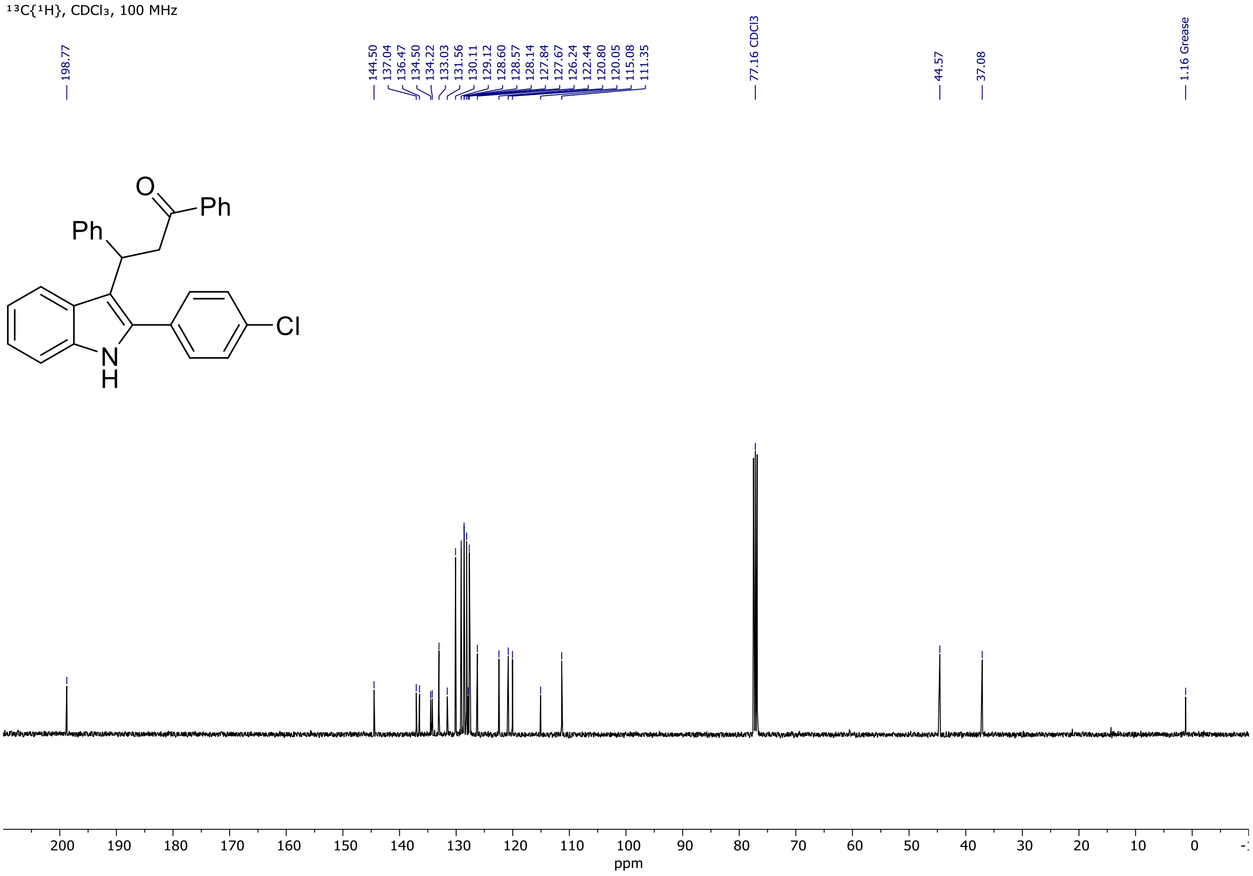

3-[2-(4-Methoxyphenyl)-1*H*-indol-3-yl]-1,3-diphenylpropan-1-one (3j).

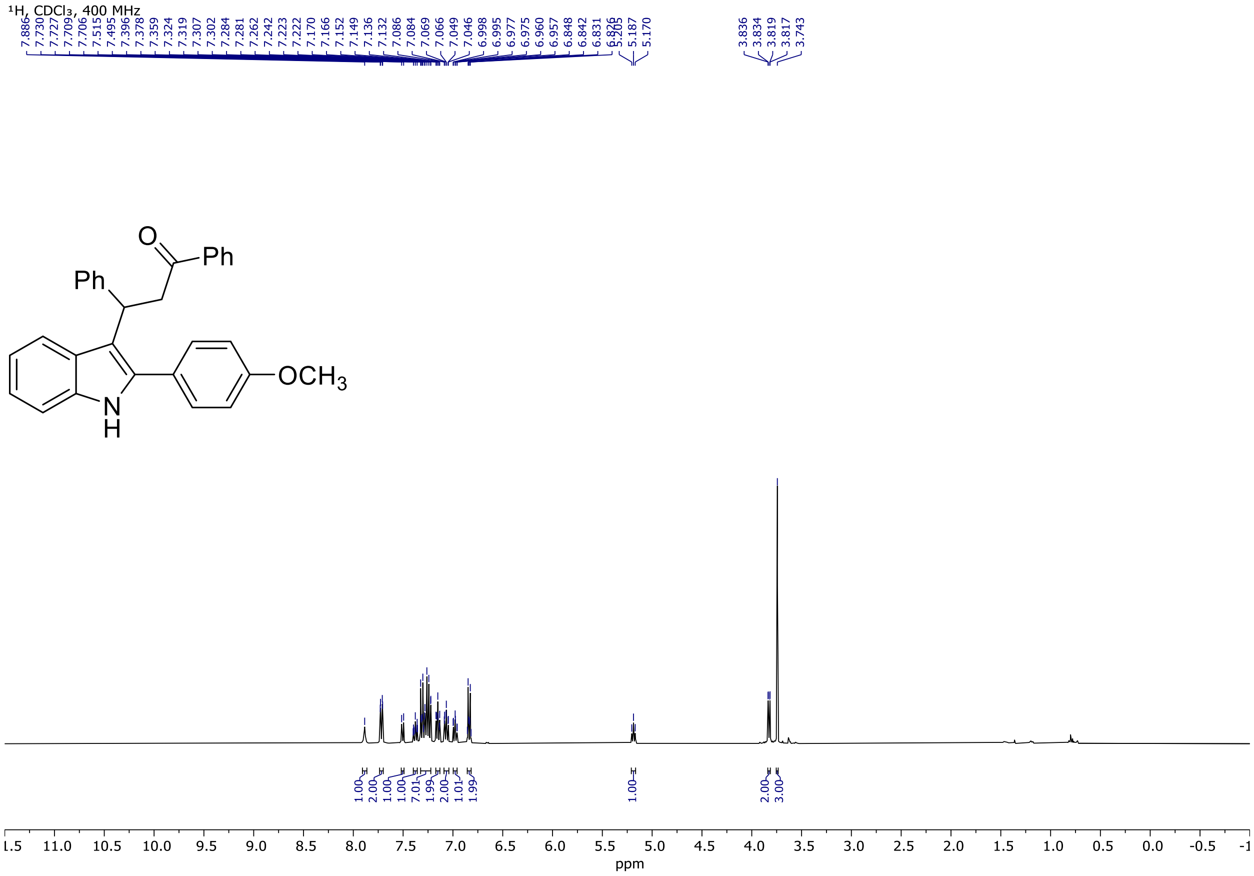

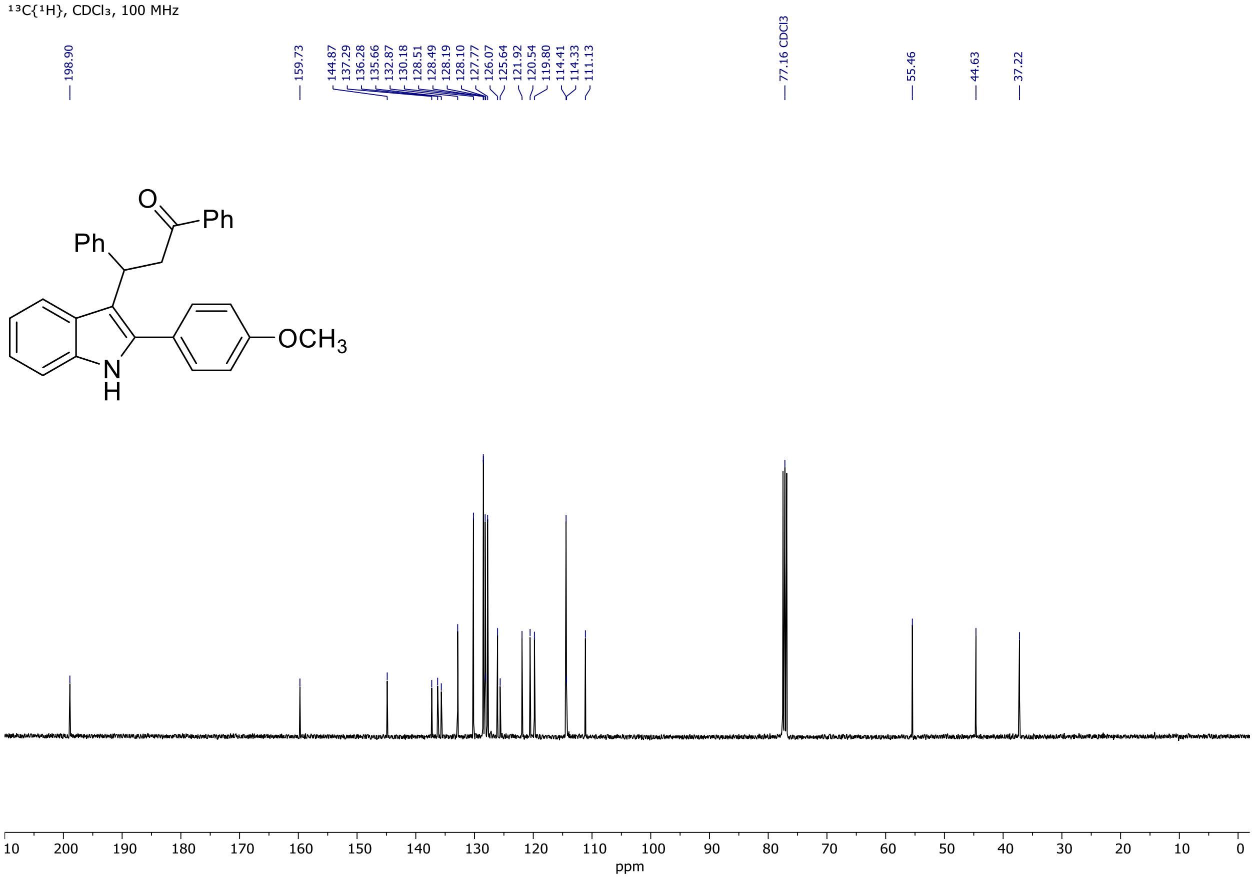

3-[2-(4-Hydroxyphenyl)-1*H*-indol-3-yl]-1,3-diphenylpropan-1-one (3k).

^{1}H, DMSO-d_6, 400 MHz

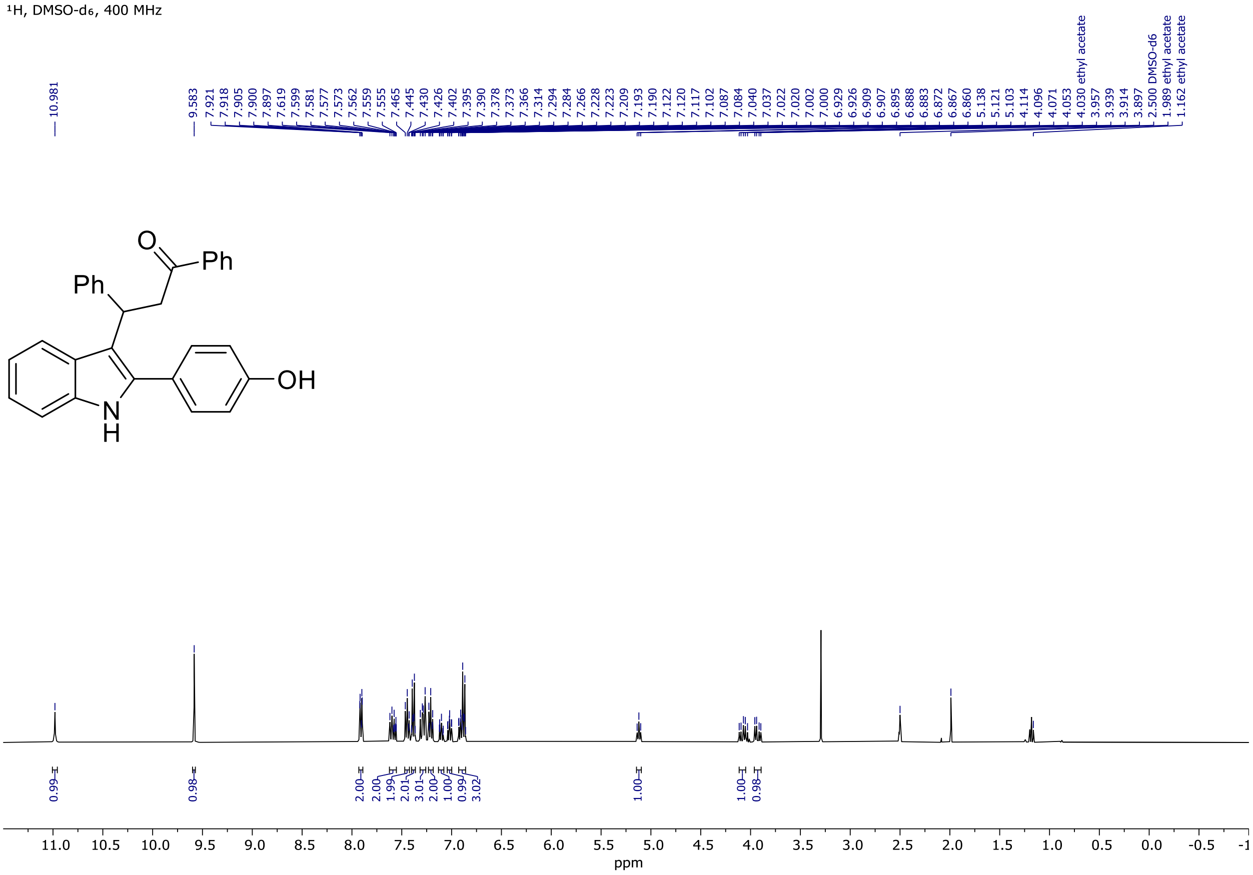

$^{13}C\{^{1}H\}$, DMSO-d_6, 100 MHz

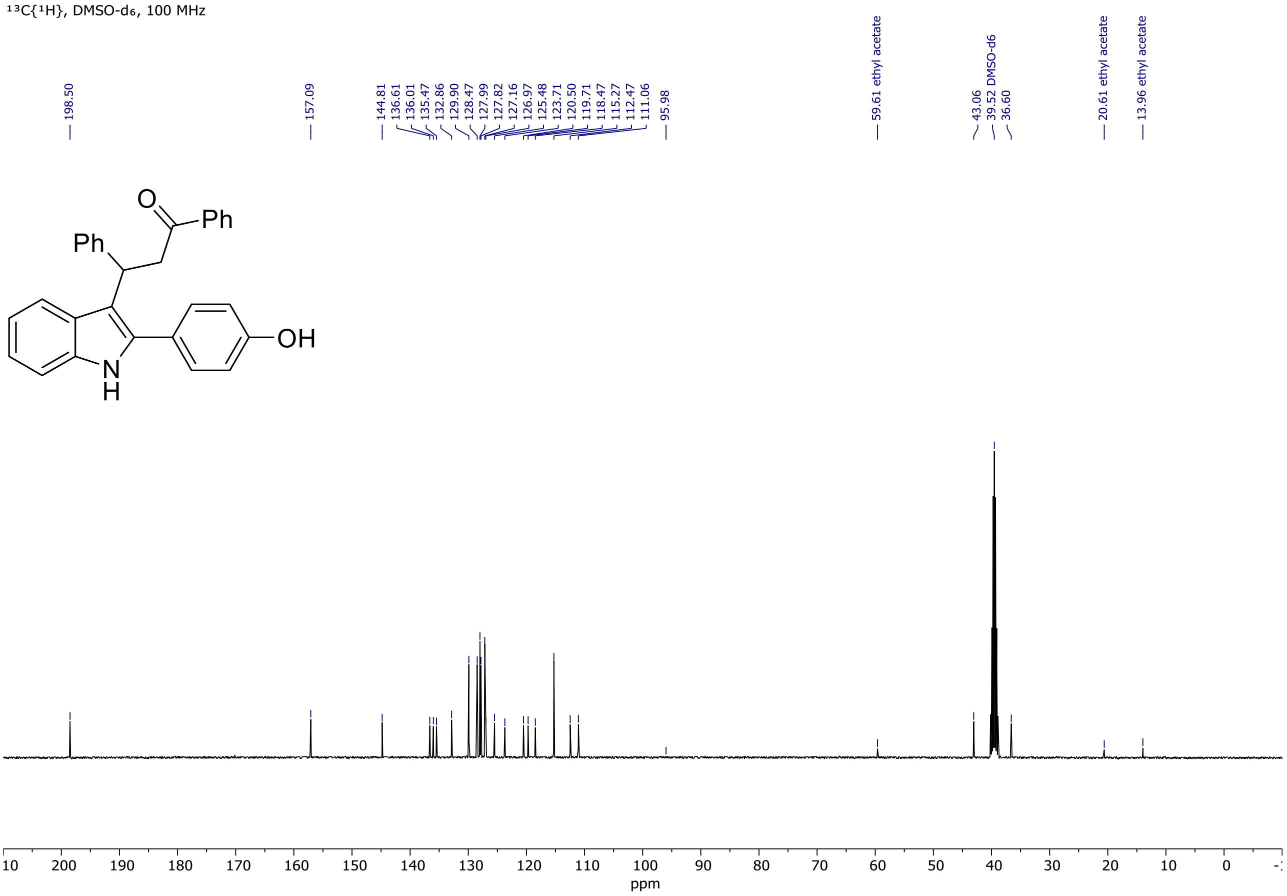

3-[2-(Naphthalen-2-yl)-1*H*-indol-3-yl]-1,3-diphenylpropan-1-one (3l).

^{1}H, $CDCl_3$, 400 MHz

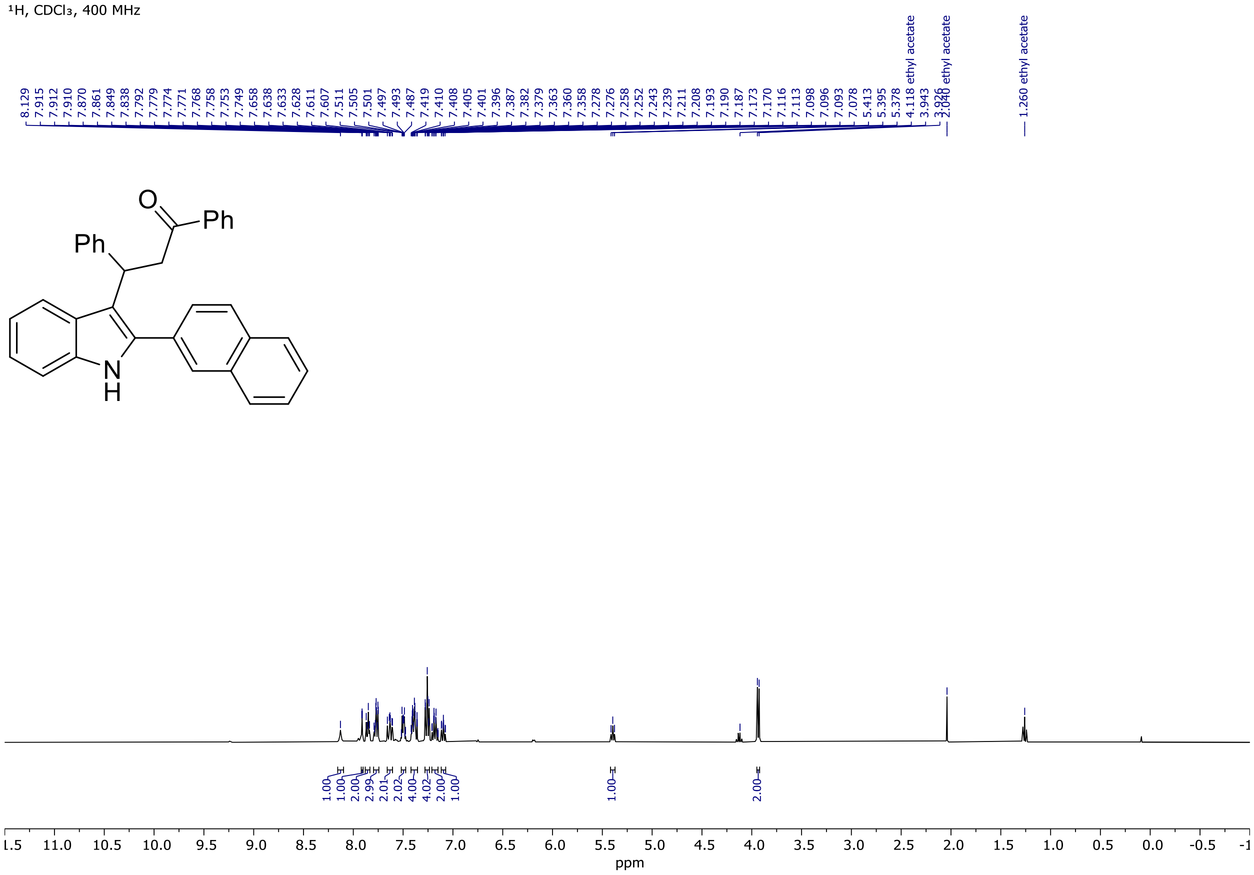

$^{13}C\{^1H\}$, $CDCl_3$, 100 MHz

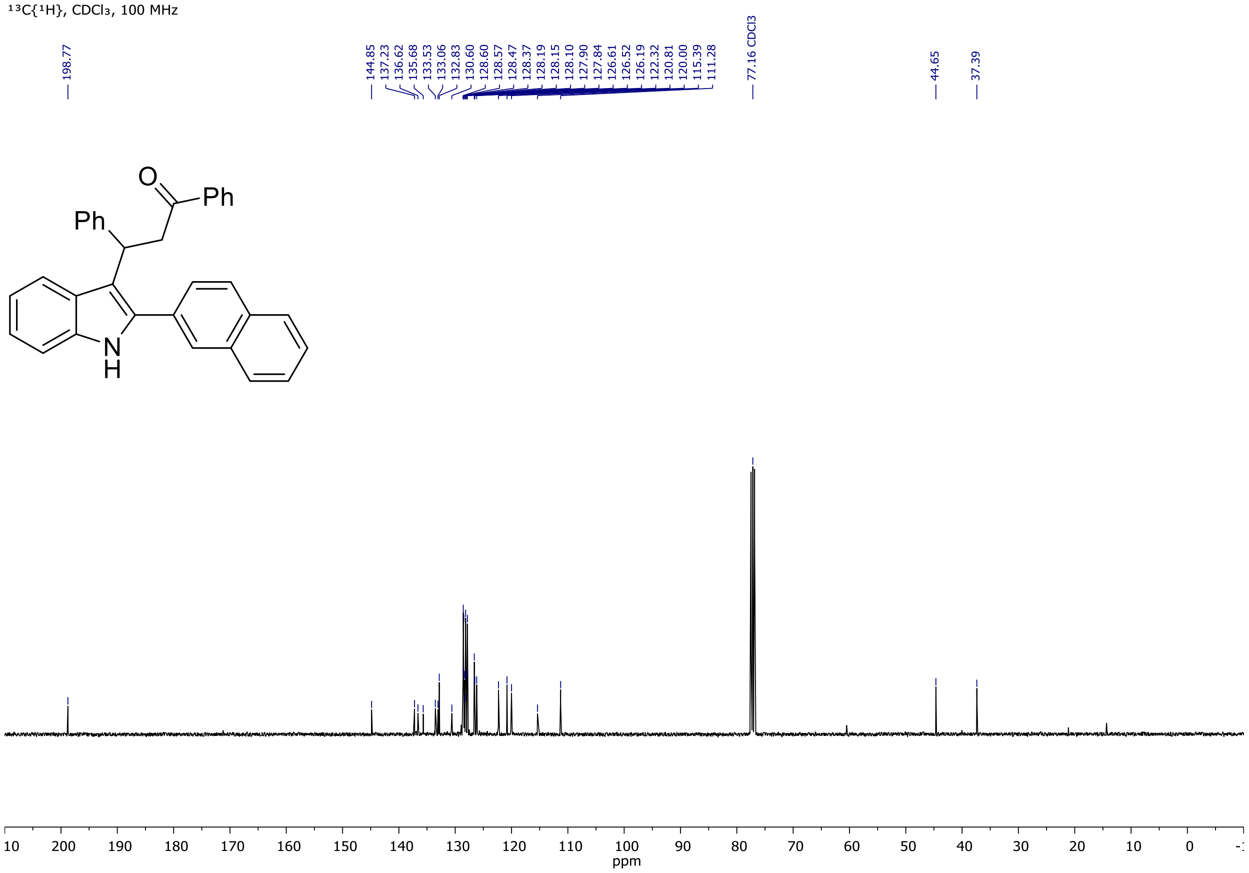

1-(2,4-Dimethylphenyl)-3-phenyl-3-[2-(*p*-tolyl)-1*H*-indol-3-yl]propan-1-one (3m).

^1H, $CDCl_3$, 400 MHz

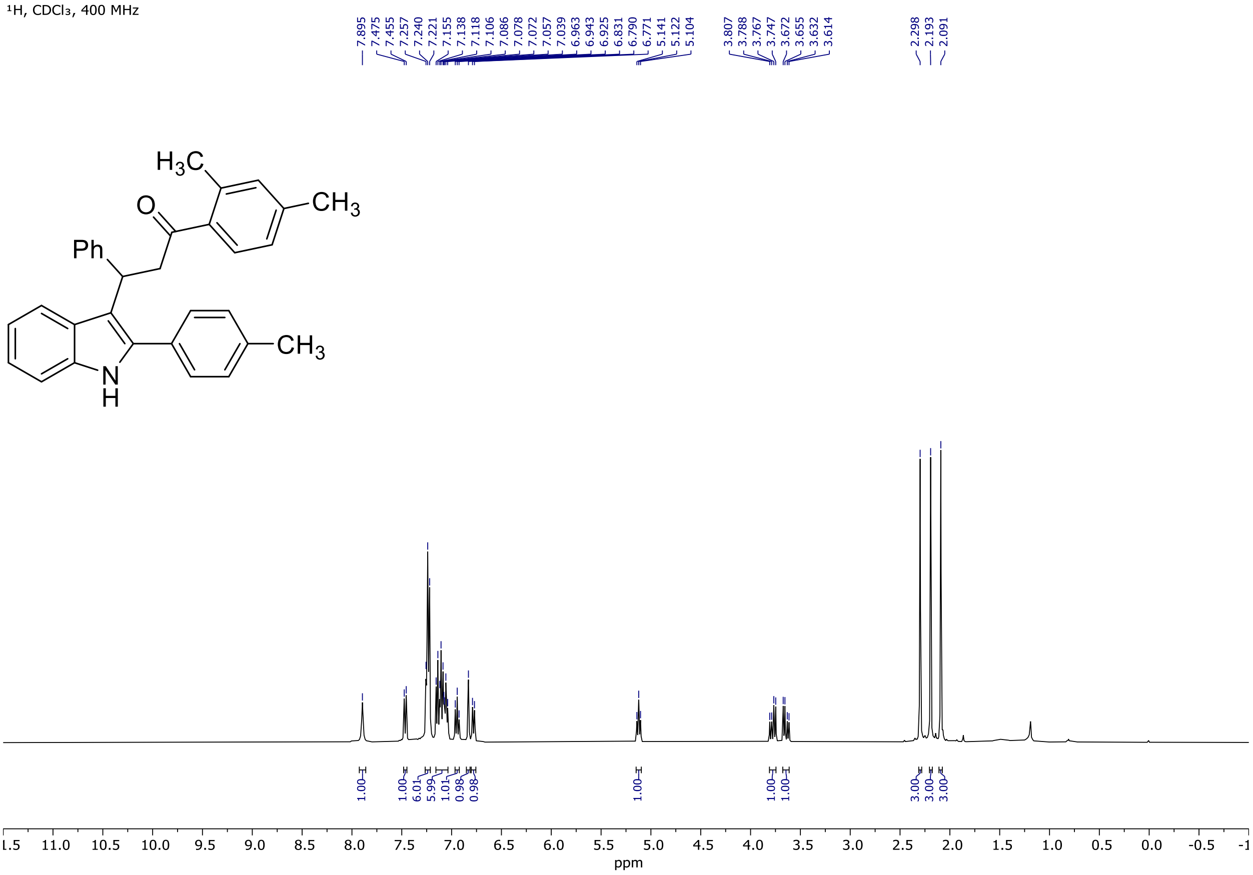

$^{13}C\{^1H\}$, $CDCl_3$, 100 MHz

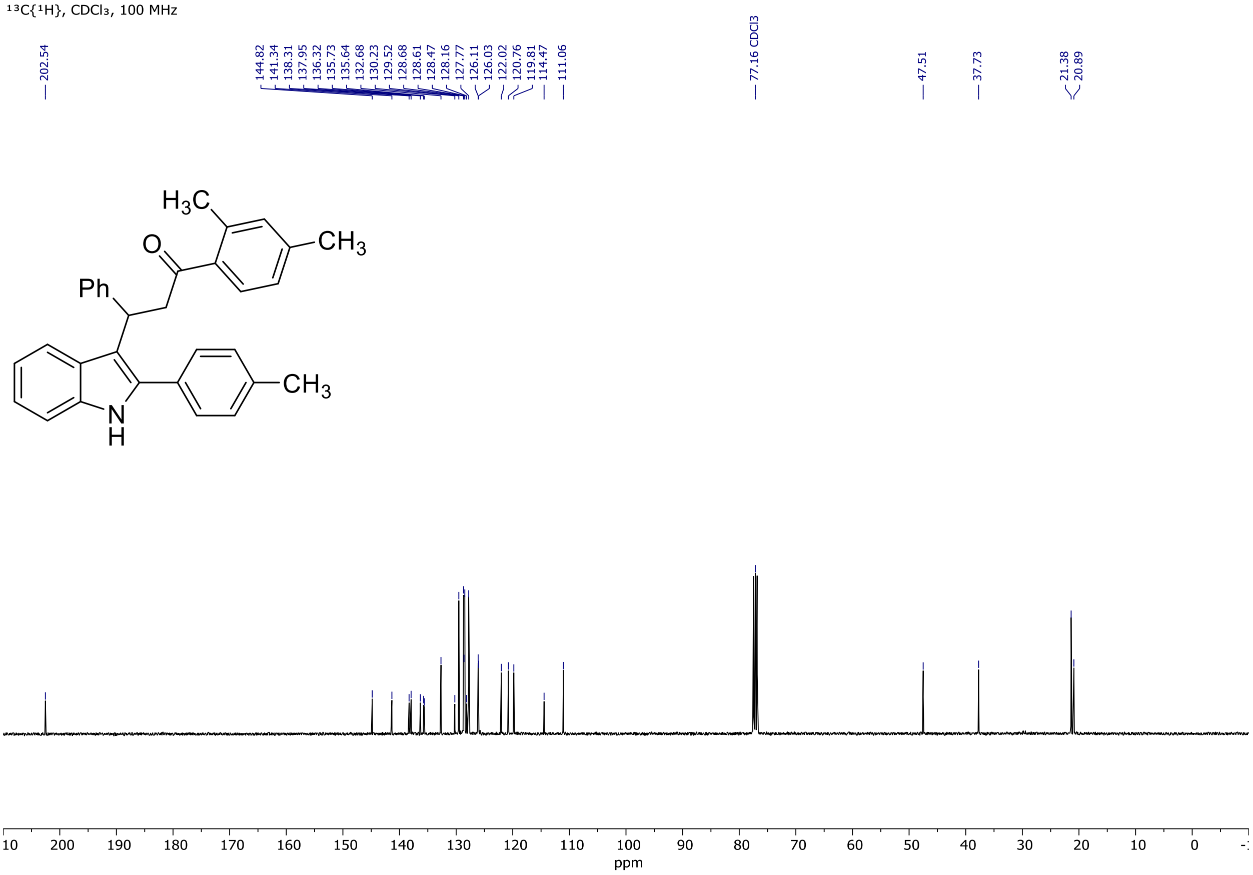

1-(Naphthalen-2-yl)-3-phenyl-3-[2-(*p*-tolyl)-1*H*-indol-3-yl]propan-1-one (3n).

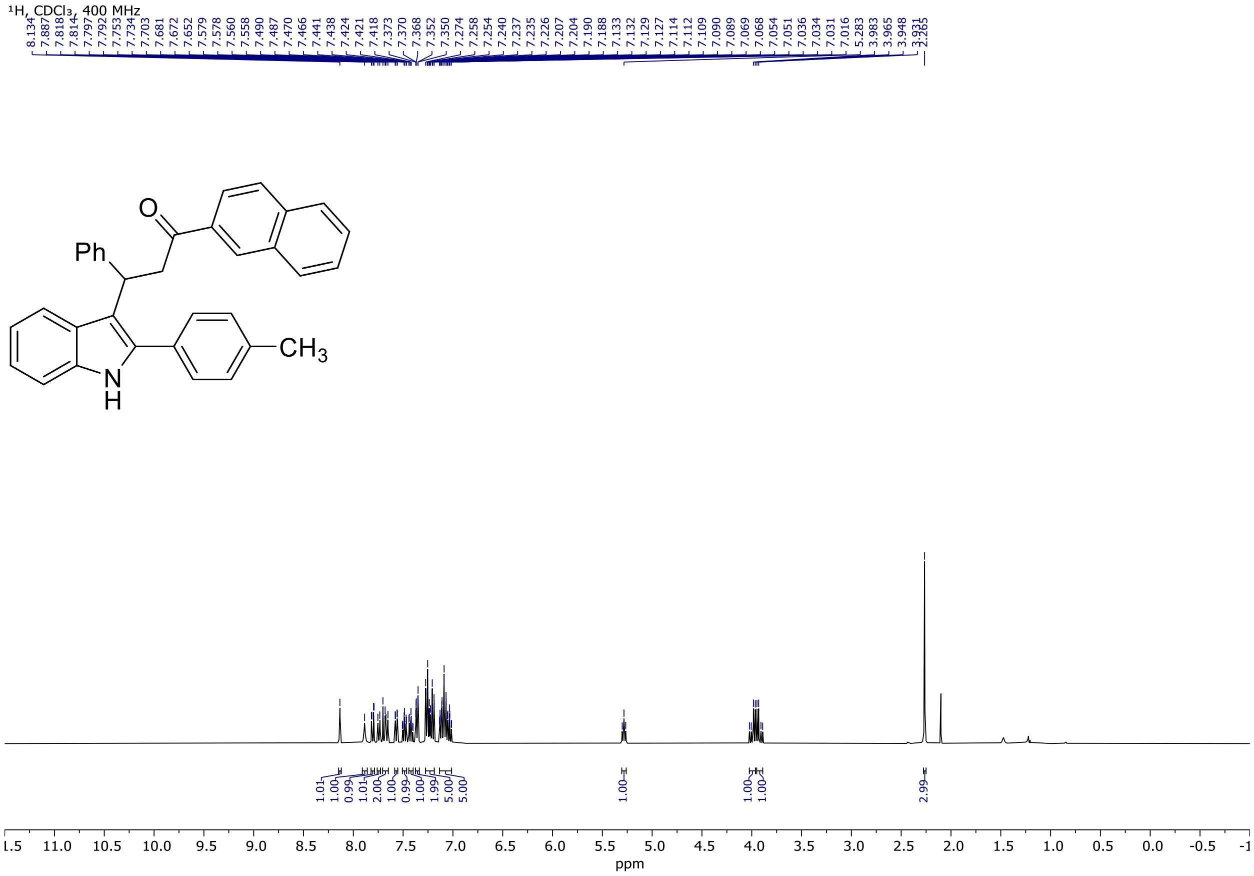

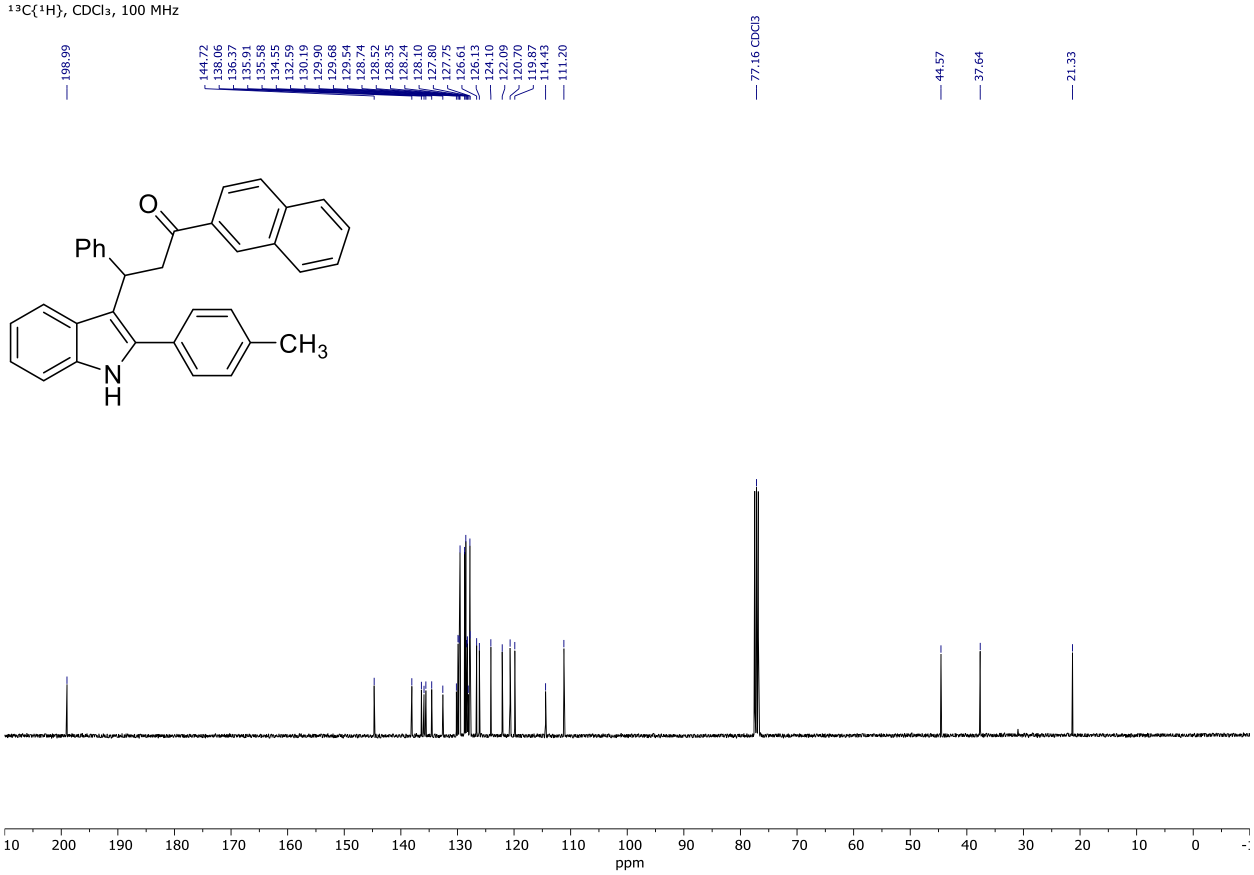

4-Phenyl-4-[2-(*p*-tolyl)-1*H*-indol-3-yl]butan-2-one (3o).

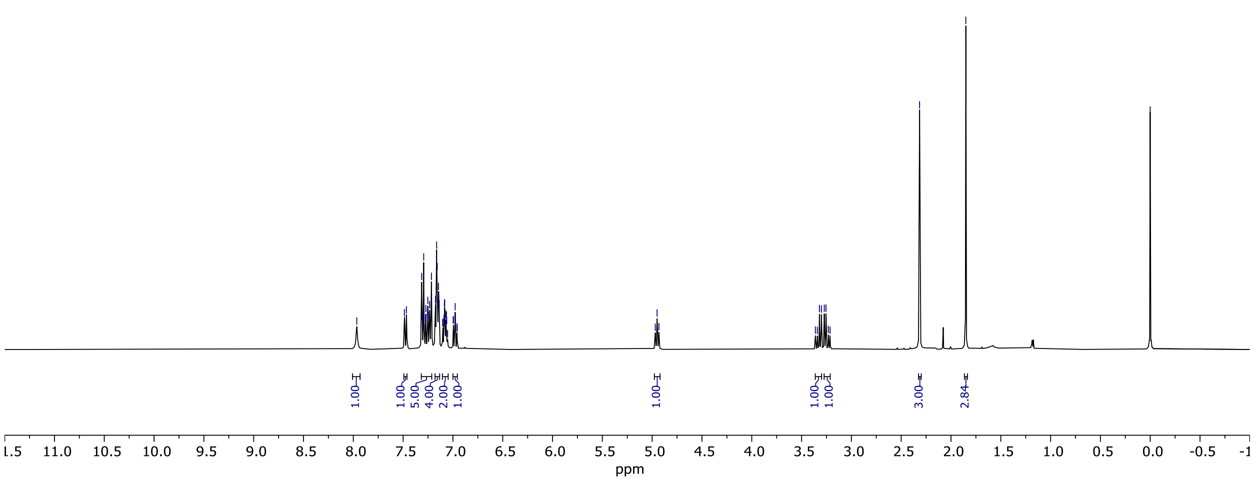

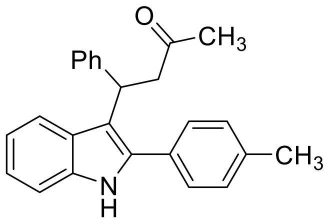

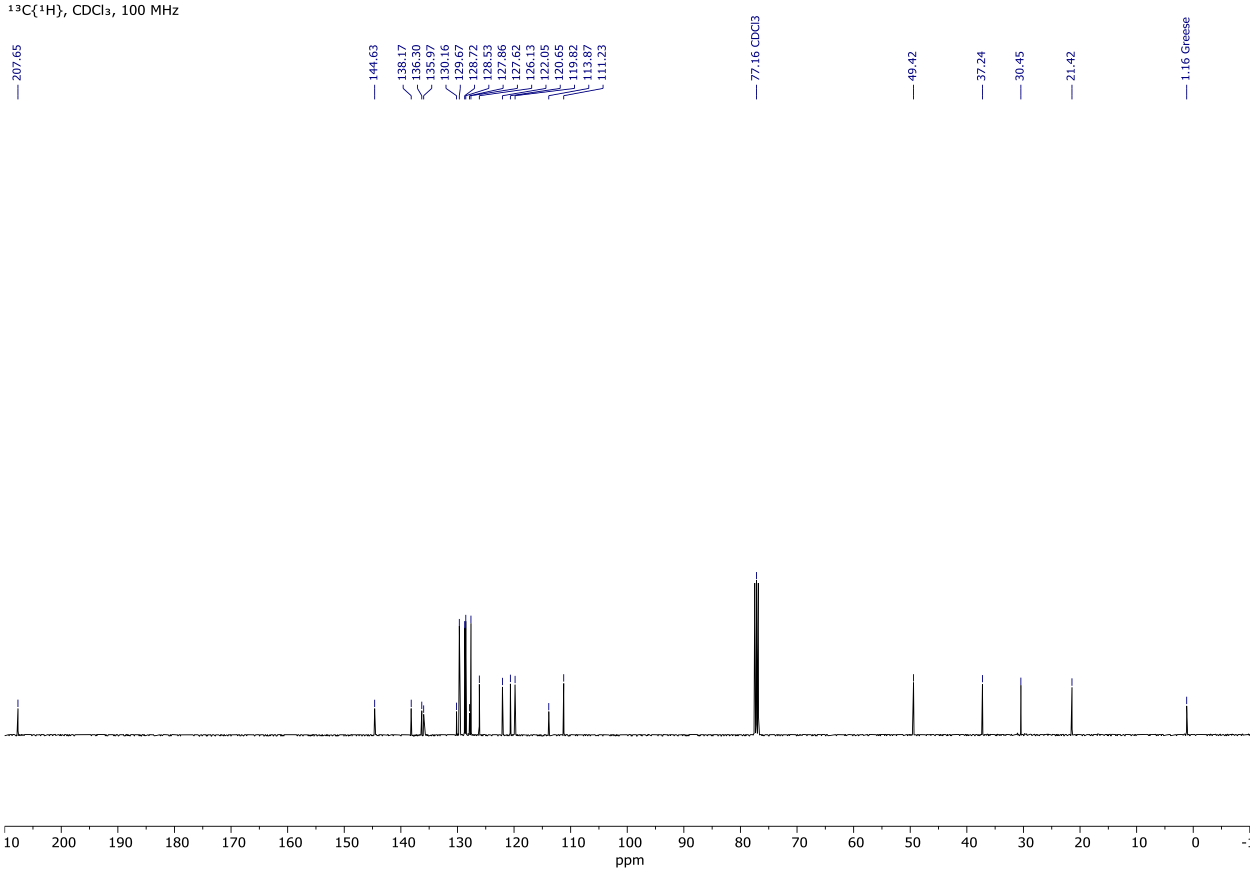

2,3',5'-Triphenyl-3',4'-dihydrospiro[indole-3,2'-pyrrole] (4a).

^{1}H, $CDCl_3$, 400 MHz

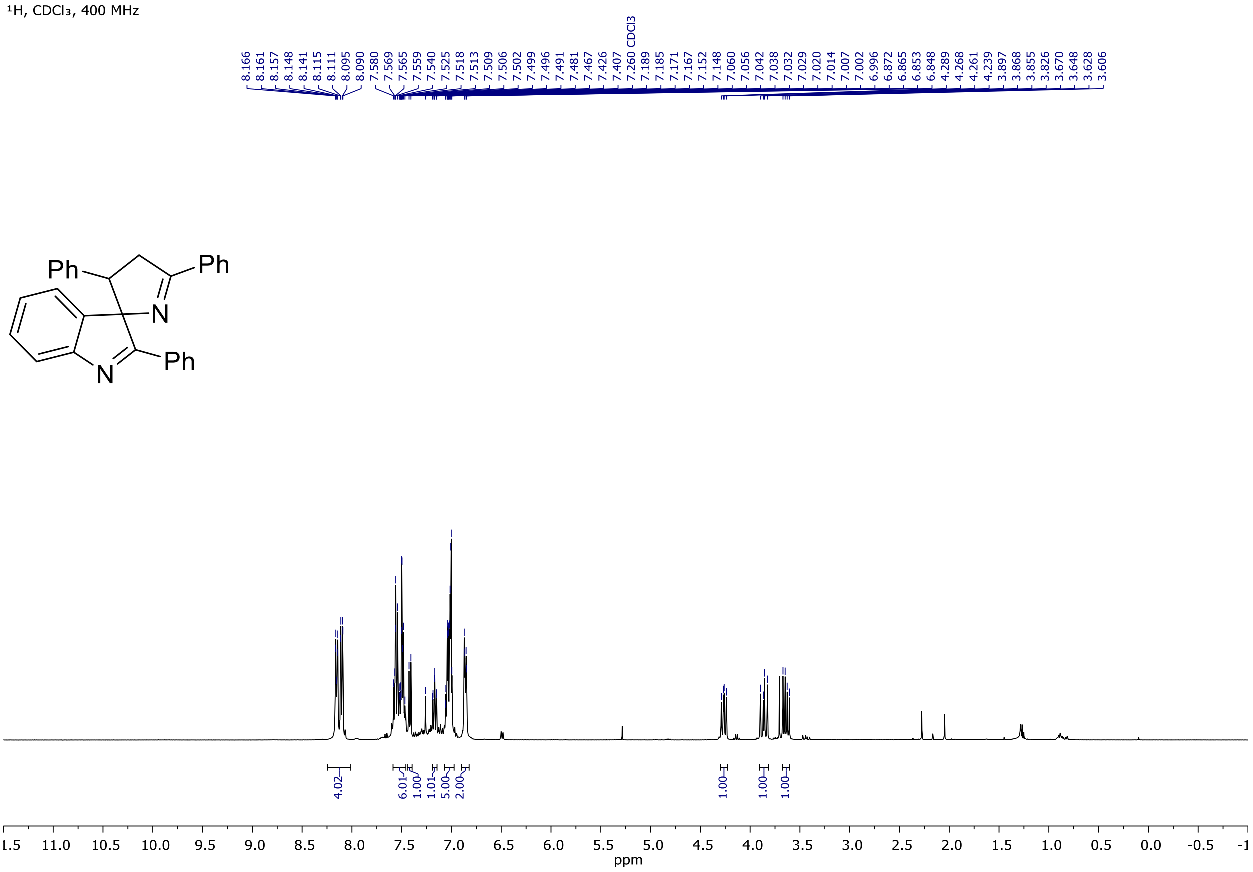

$^{13}C\{^{1}H\}$, $CDCl_3$, 100 MHz

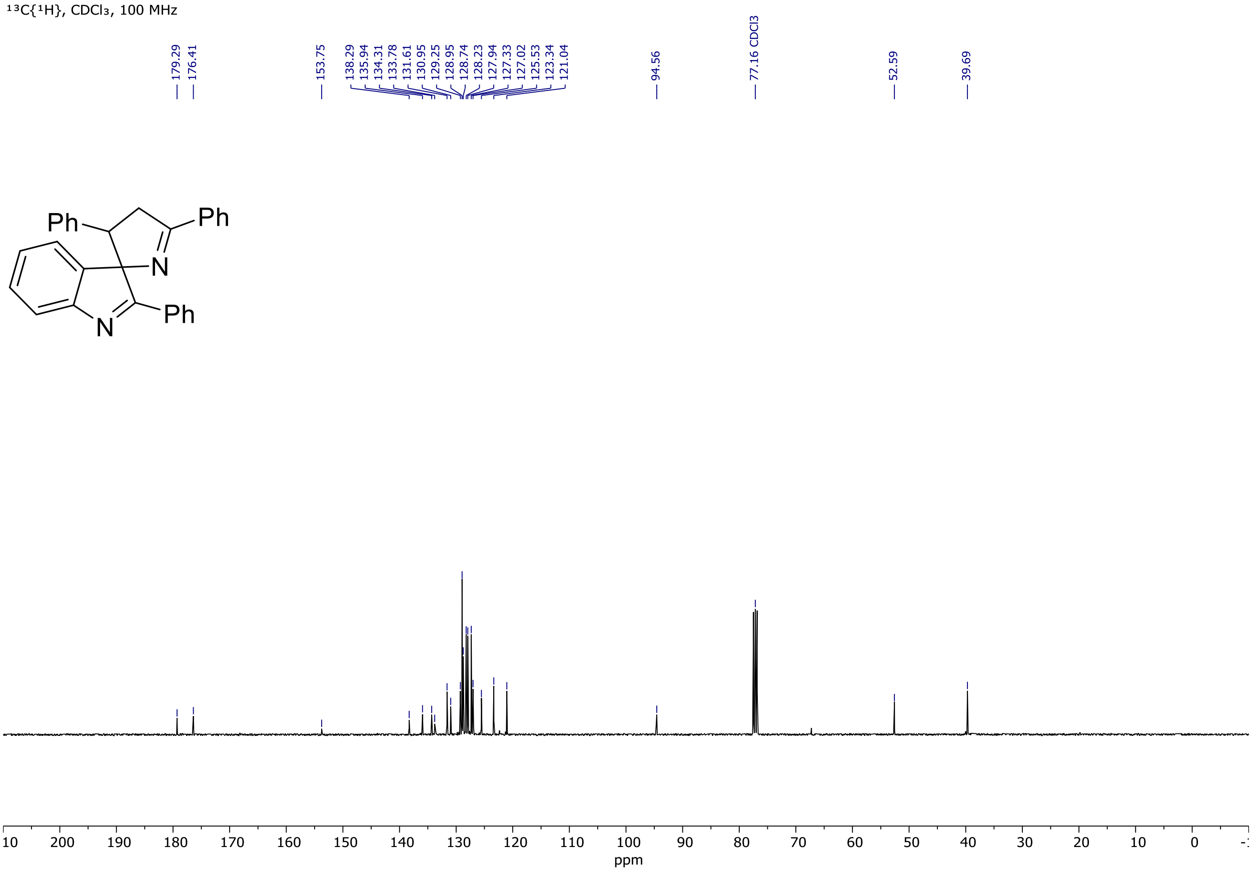

2,5'-Diphenyl-3'-(*p*-tolyl)-3',4'-dihydrospiro[indole-3,2'-pyrrole] (4b).

^1H, $CDCl_3$, 400 MHz

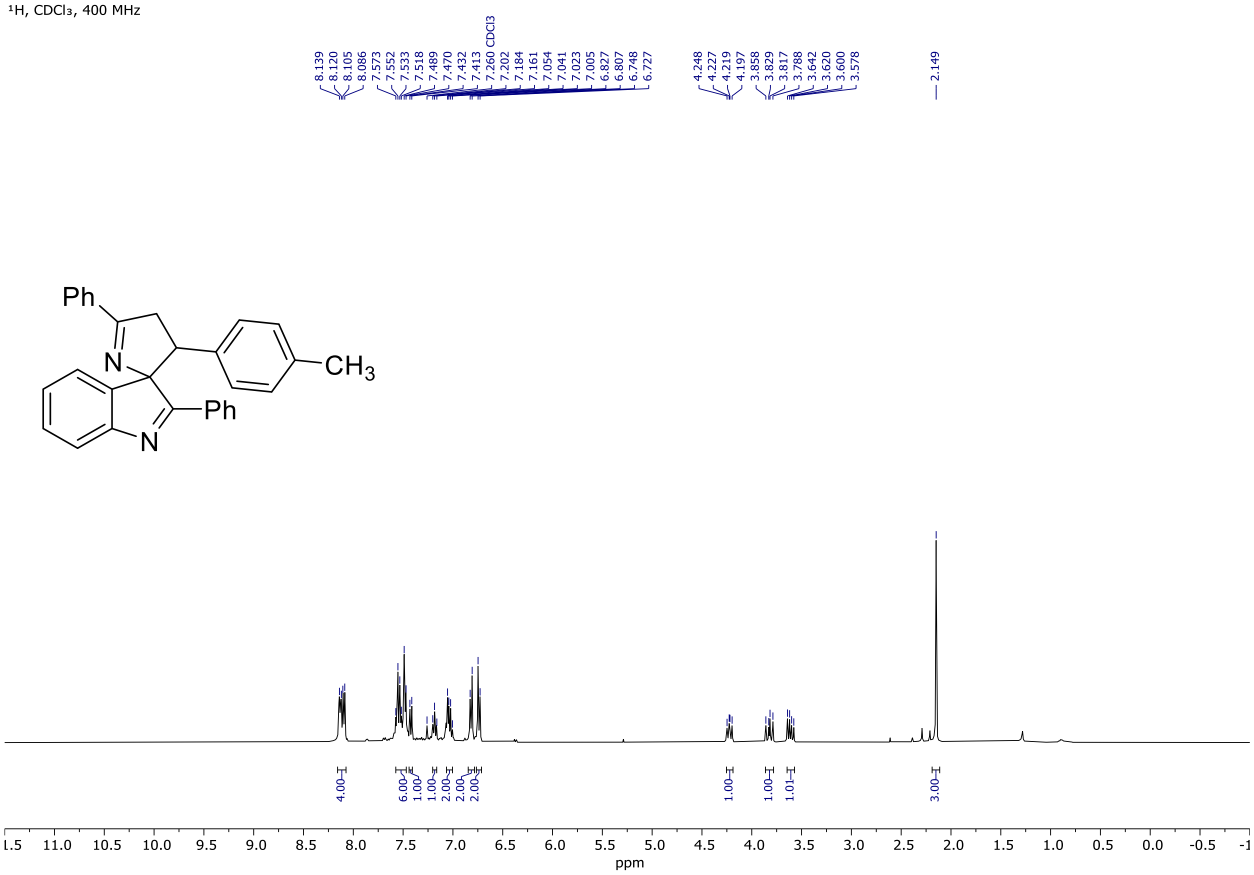

$^{13}C\{^1H\}$, $CDCl_3$, 100 MHz

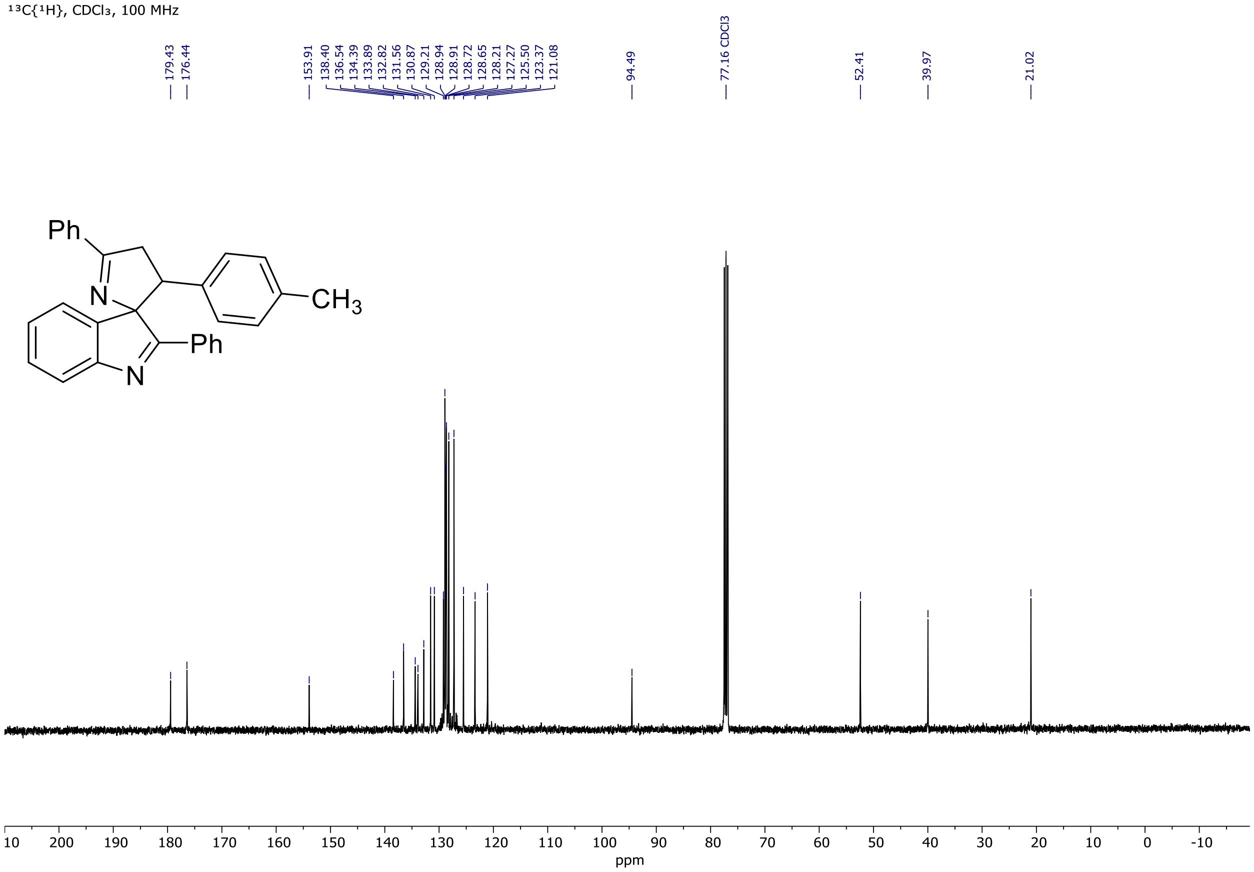

3'-(4-Methoxyphenyl)-2,5'-diphenyl-3',4'-dihydrospiro[indole-3,2'-pyrrole] (4c).

^1H, $CDCl_3$, 400 MHz

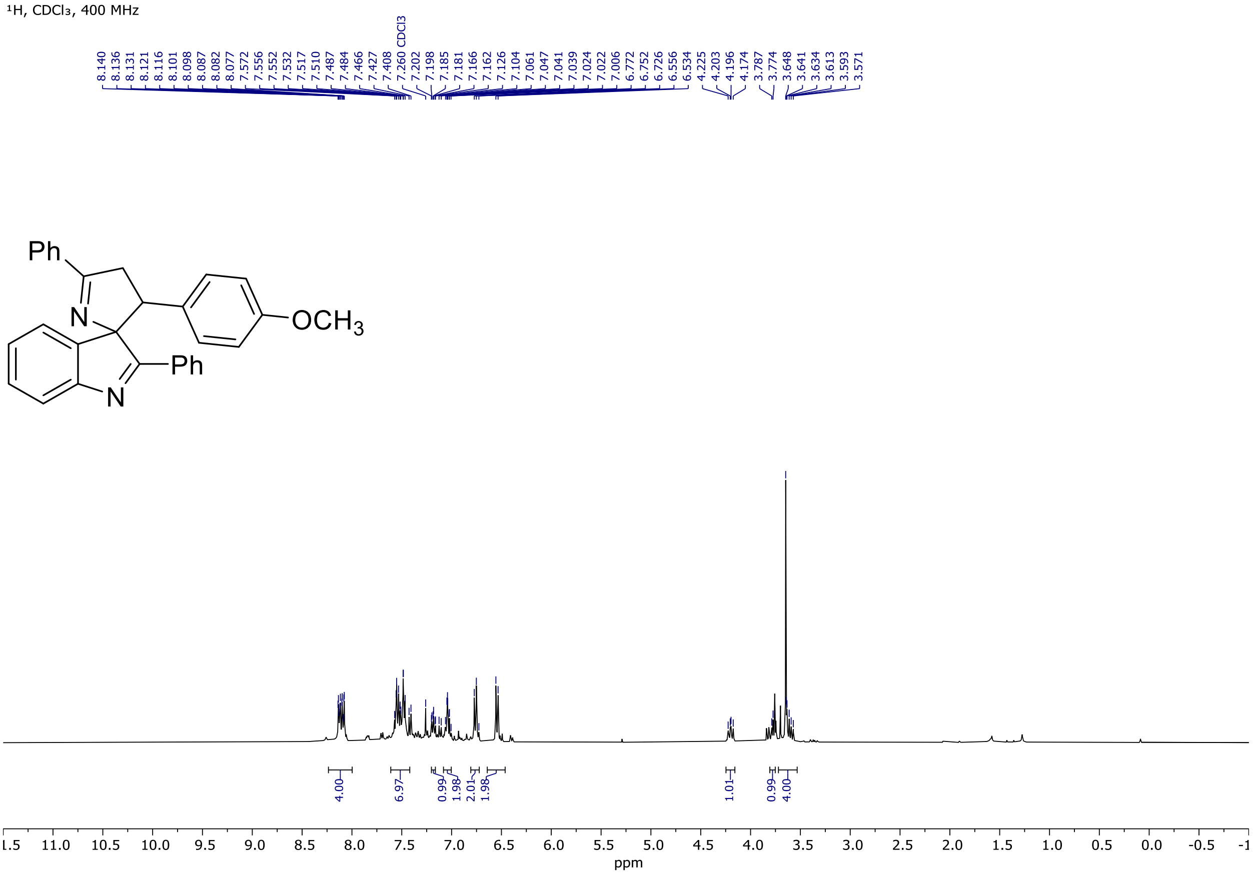

$^{13}C\{^1H\}$, $CDCl_3$, 100 MHz

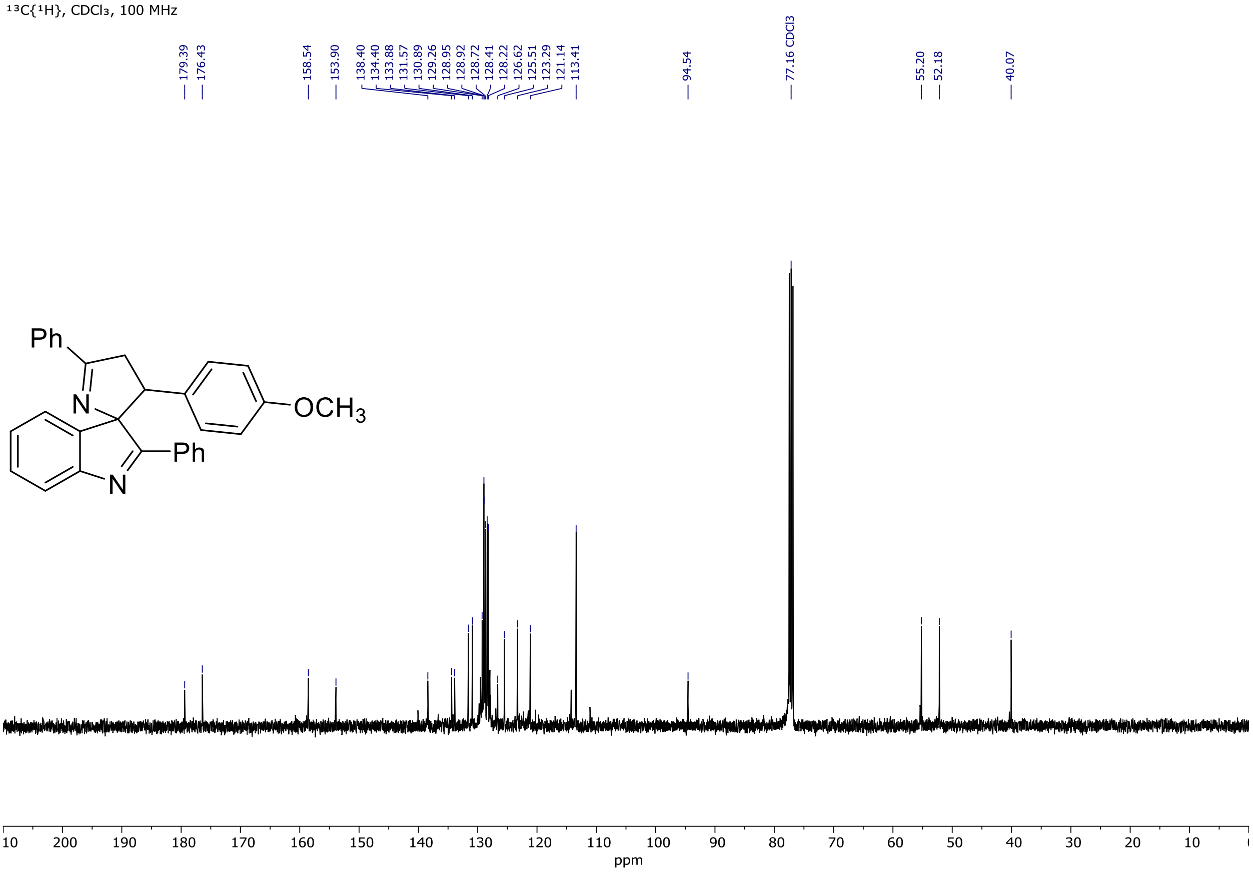

2,5'-Diphenyl-3'-[4-(trifluoromethyl)phenyl]-3',4'-dihydrospiro[indole-3,2'-pyrrole] (4d).

^1H, $CDCl_3$, 400 MHz

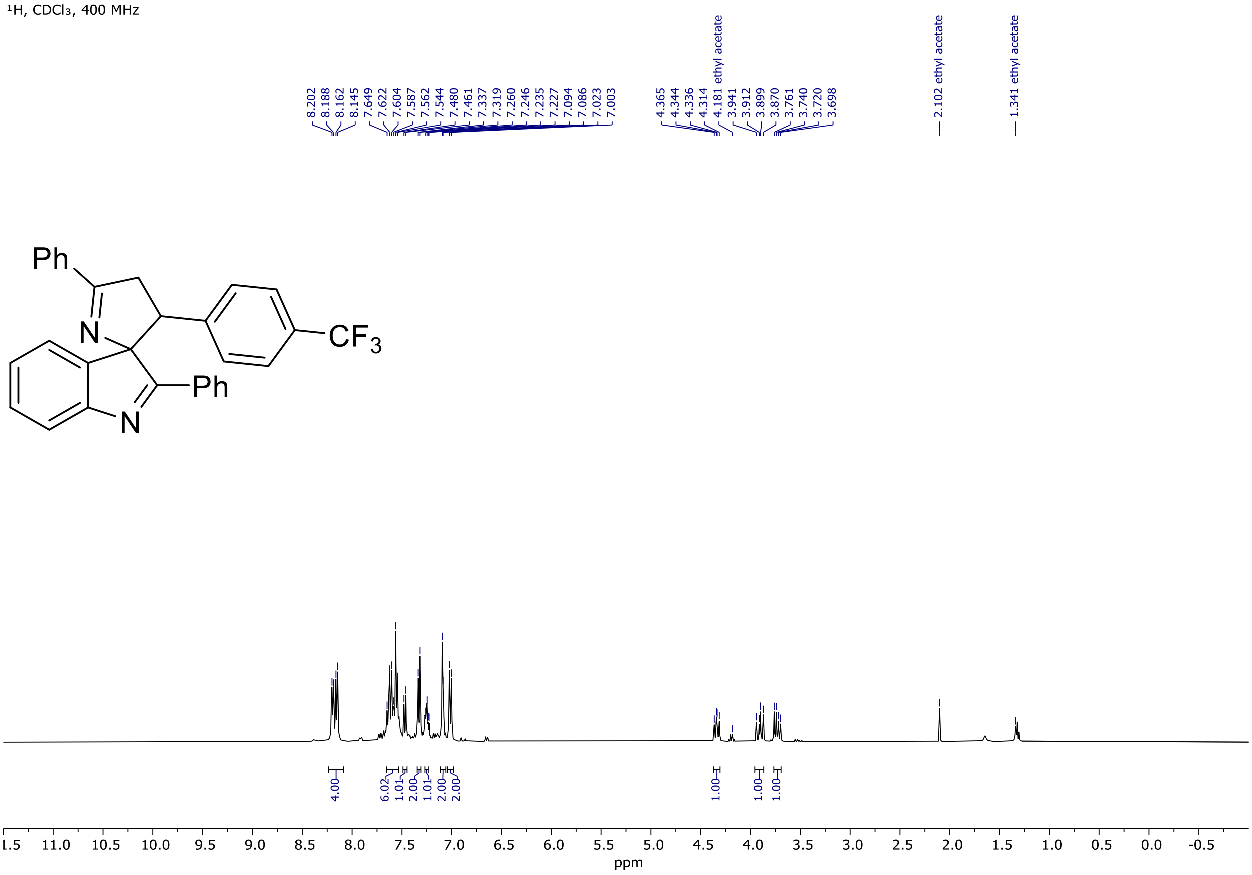

$^{13}C\{^1H\}$, $CDCl_3$, 100 MHz

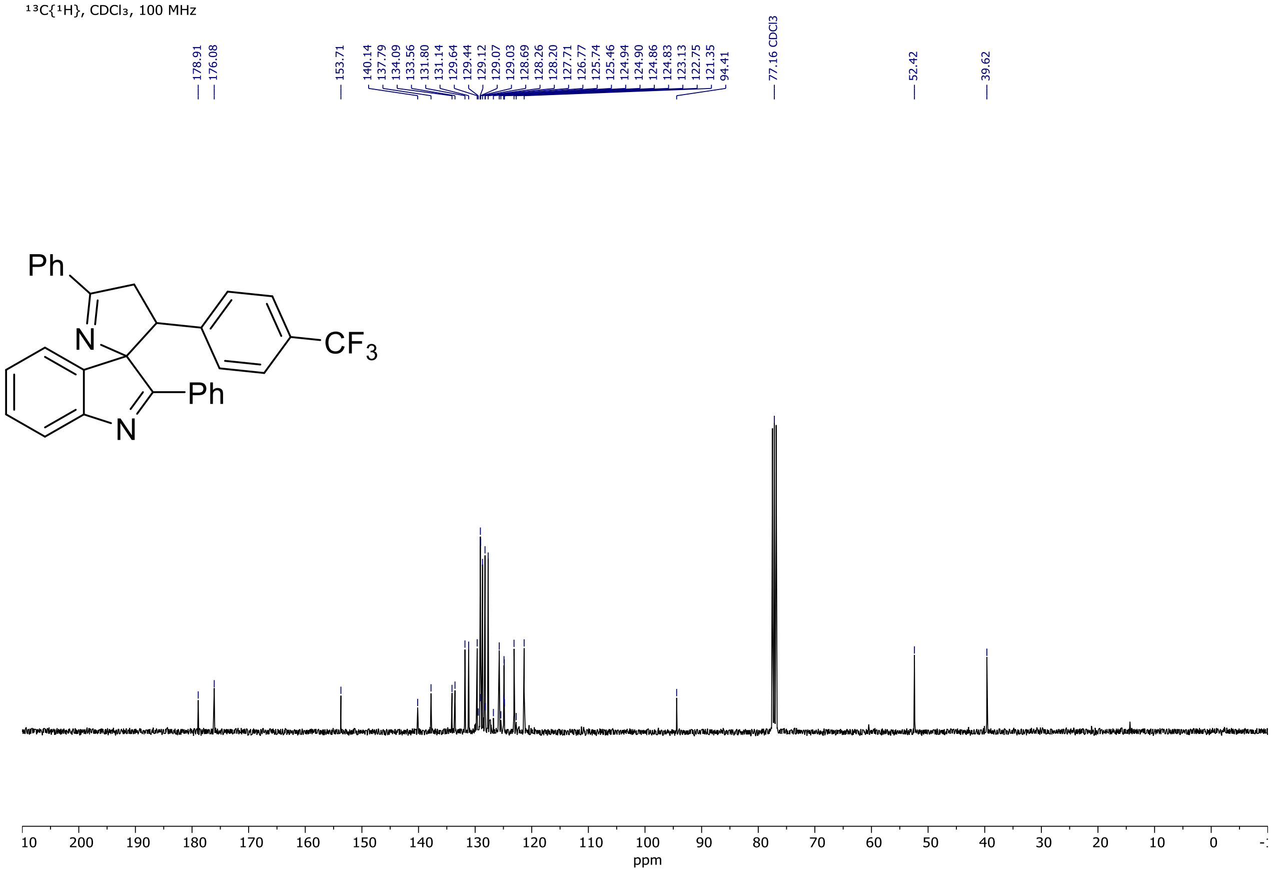

¹⁹F, $CDCl_3$, 376 MHz

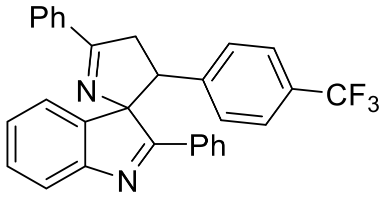

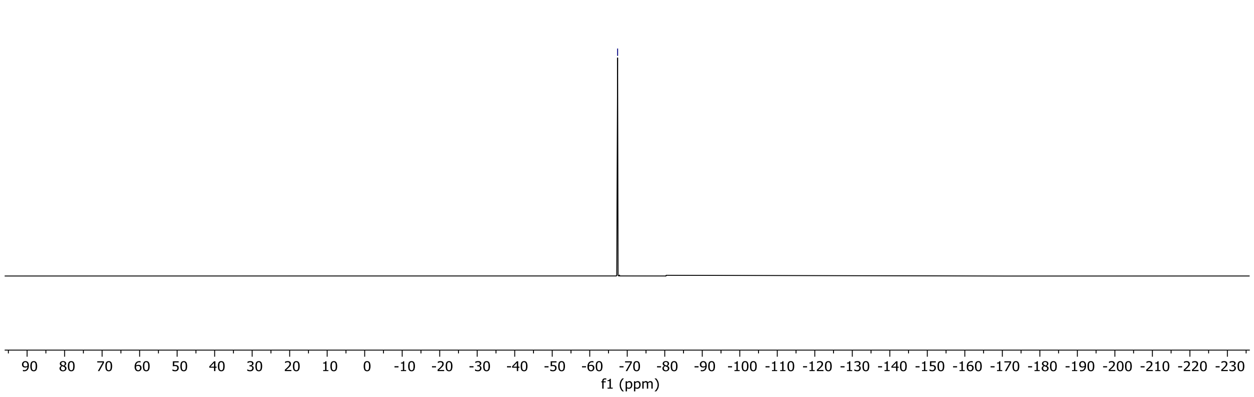

2,3'-Diphenyl-5'-(*p*-tolyl)-3',4'-dihydrospiro[indole-3,2'-pyrrole] (4e).

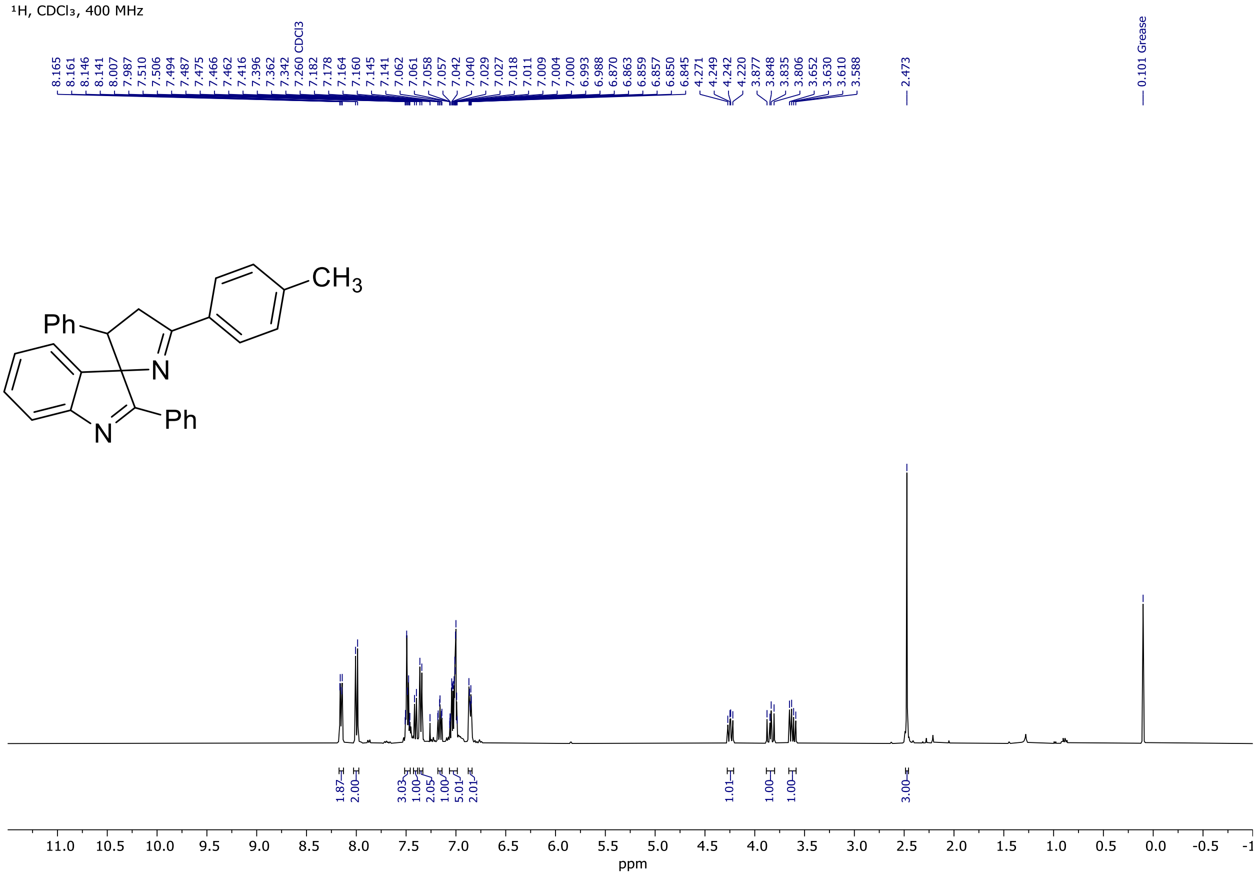

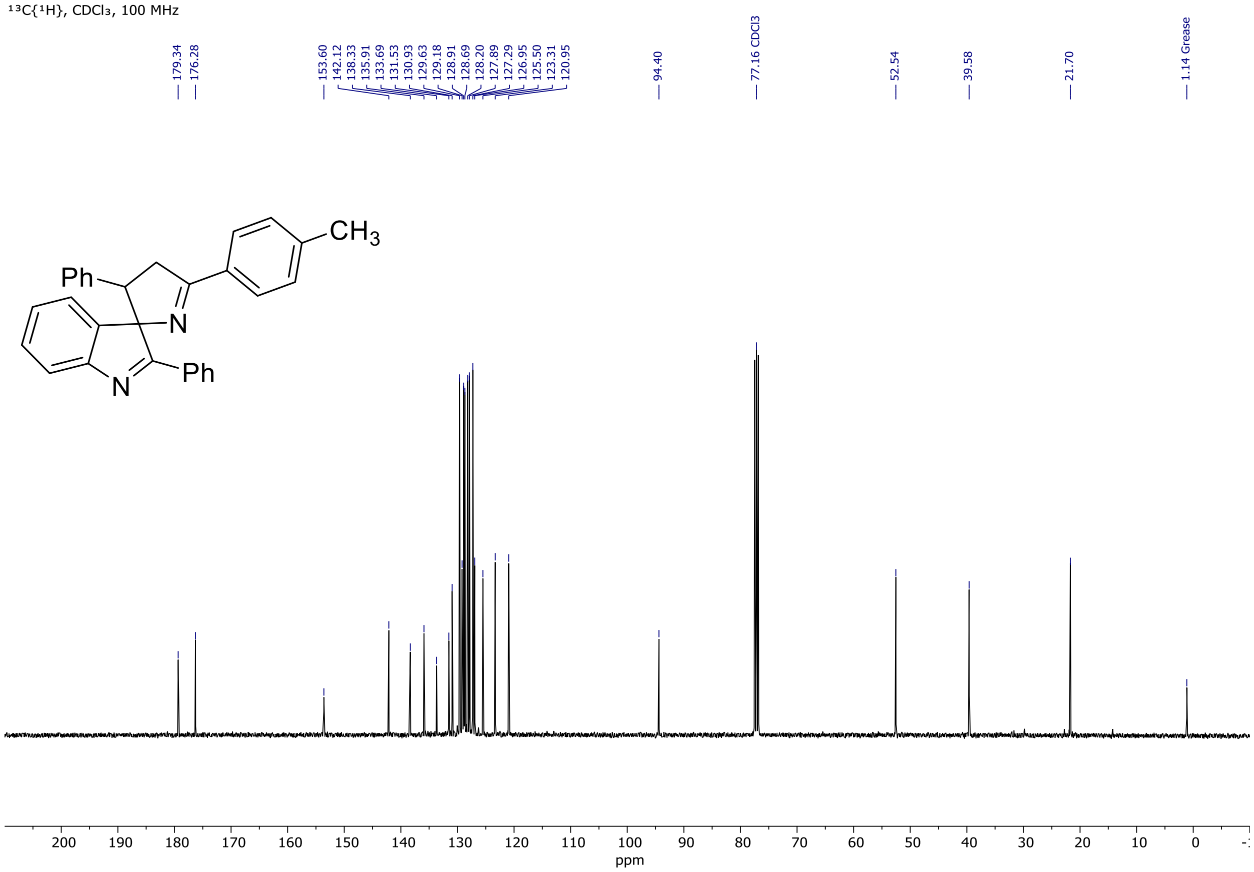

5'-(4-Methoxyphenyl)-2,3'-diphenyl-3',4'-dihydrospiro[indole-3,2'-pyrrole] (4f).

^{1}H, $CDCl_3$, 400 MHz

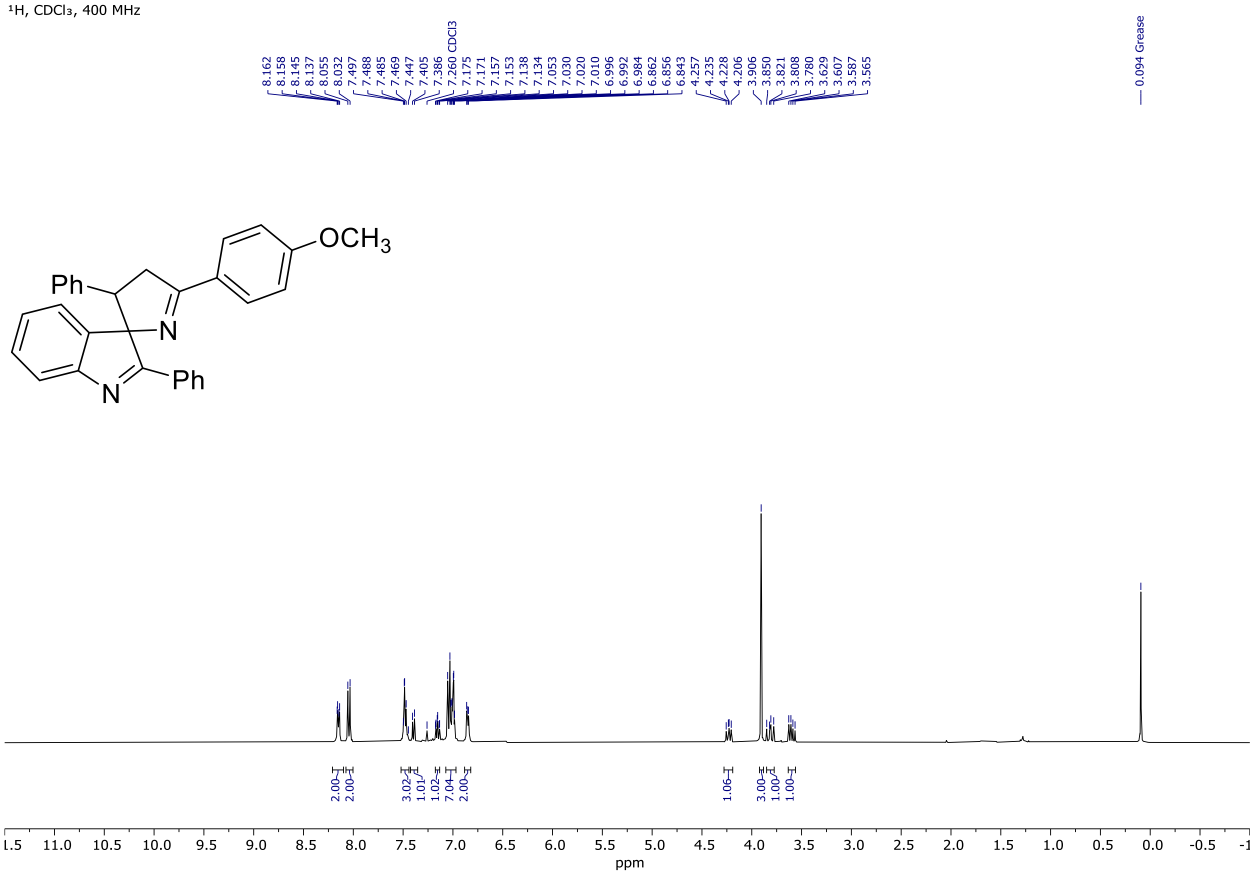

$^{13}C\{^{1}H\}$, $CDCl_3$, 100 MHz

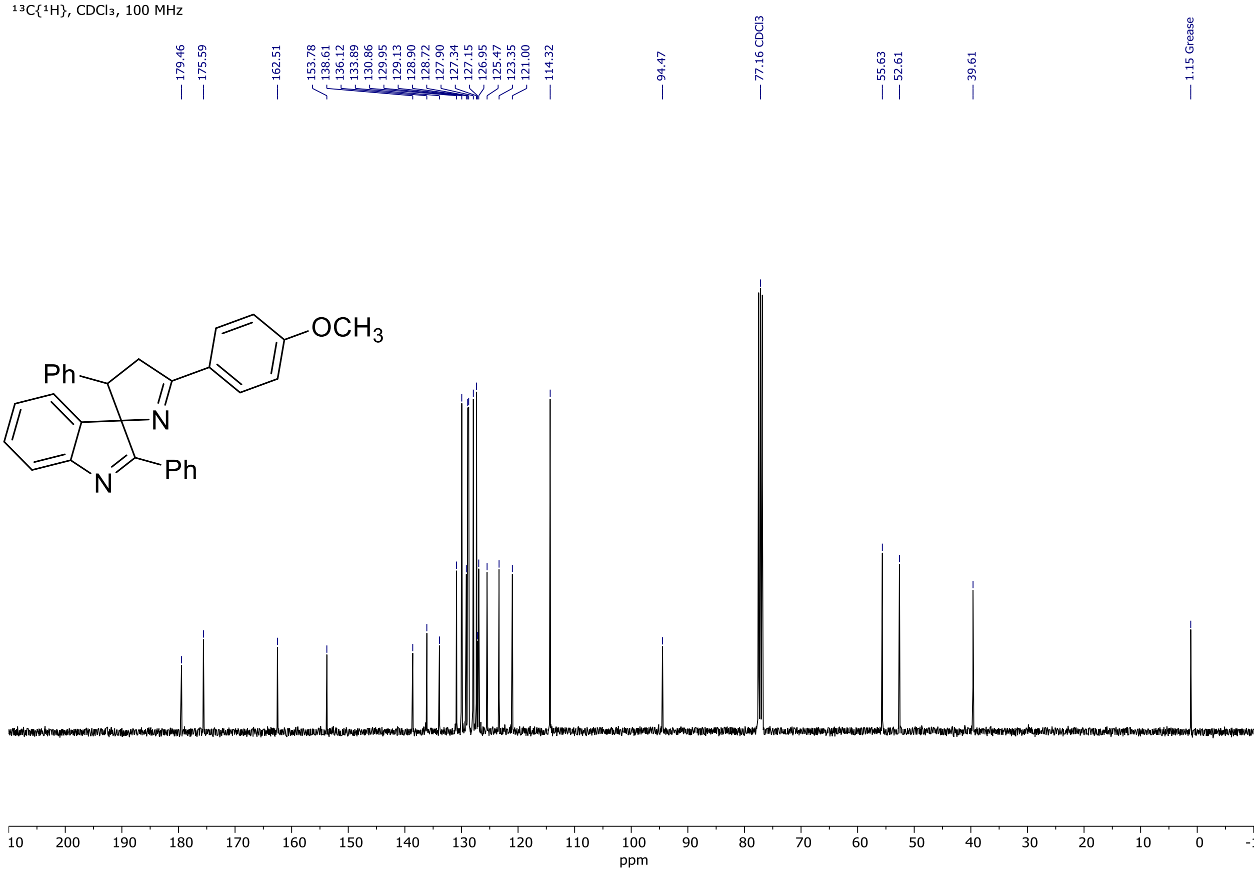

5'-(3,4-Dichlorophenyl)-2,3'-diphenyl-3',4'-dihydrospiro[indole-3,2'-pyrrole] (4g).

^{1}H, $CDCl_3$, 400 MHz

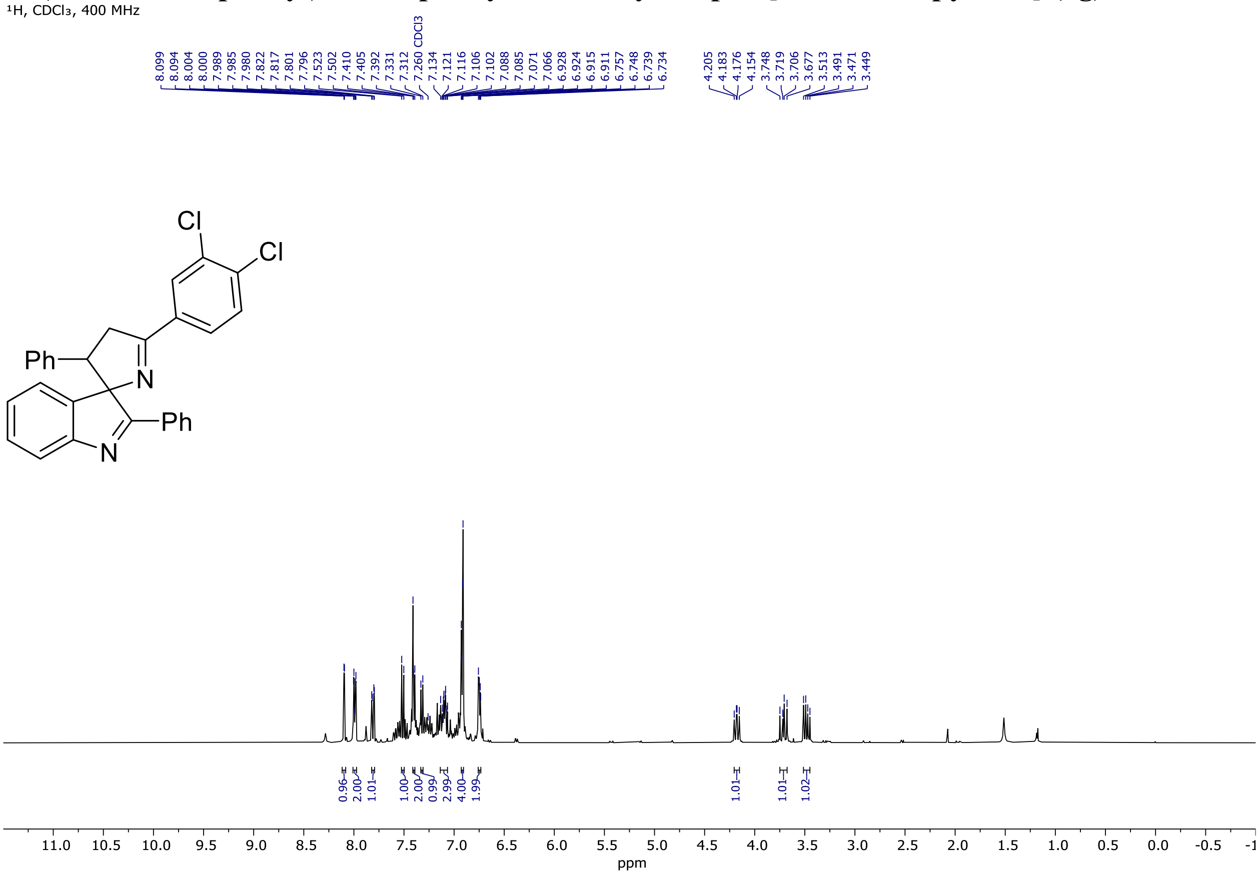

$^{13}C\{^{1}H\}$, $CDCl_3$, 100 MHz

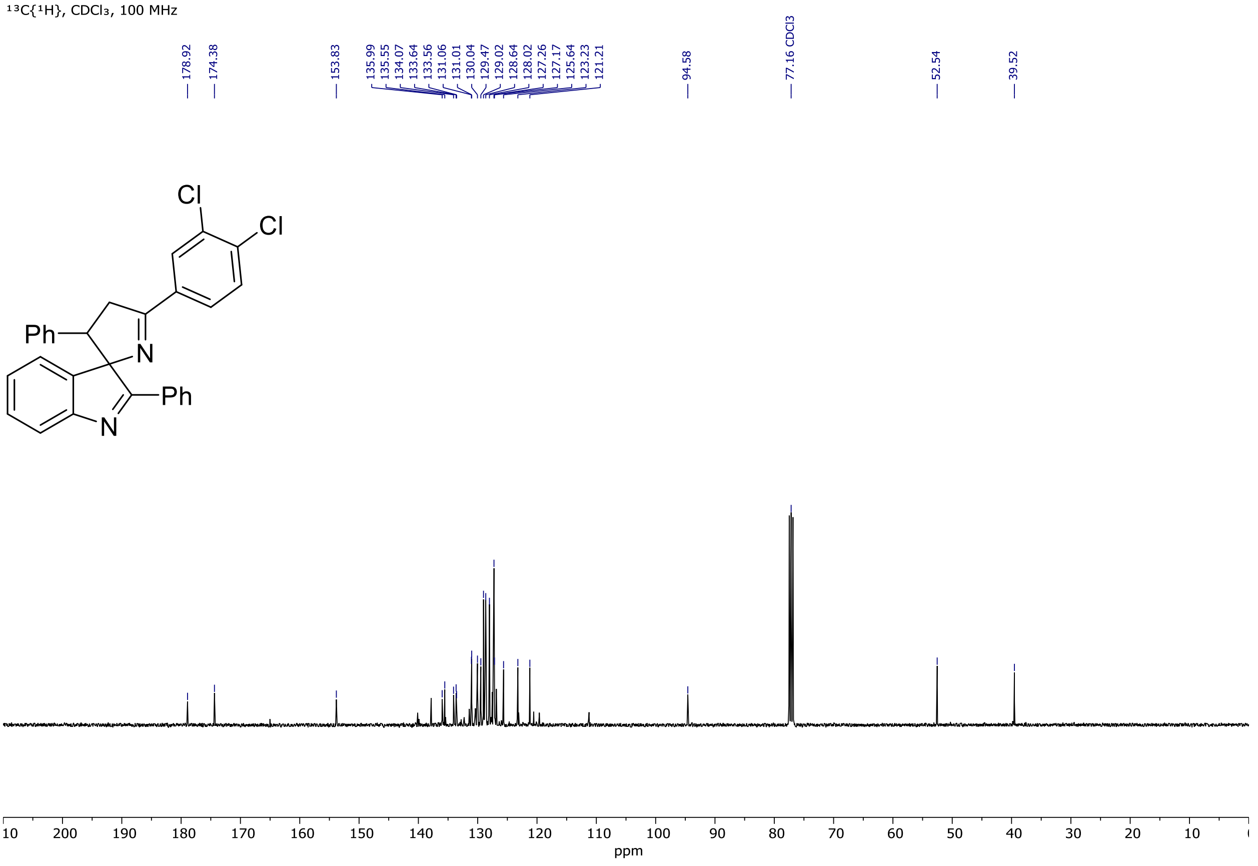

3',5'-Diphenyl-2-(*p*-tolyl)-3',4'-dihydrospiro[indole-3,2'-pyrrole] (4h).

^{1}H, $CDCl_3$, 400 MHz

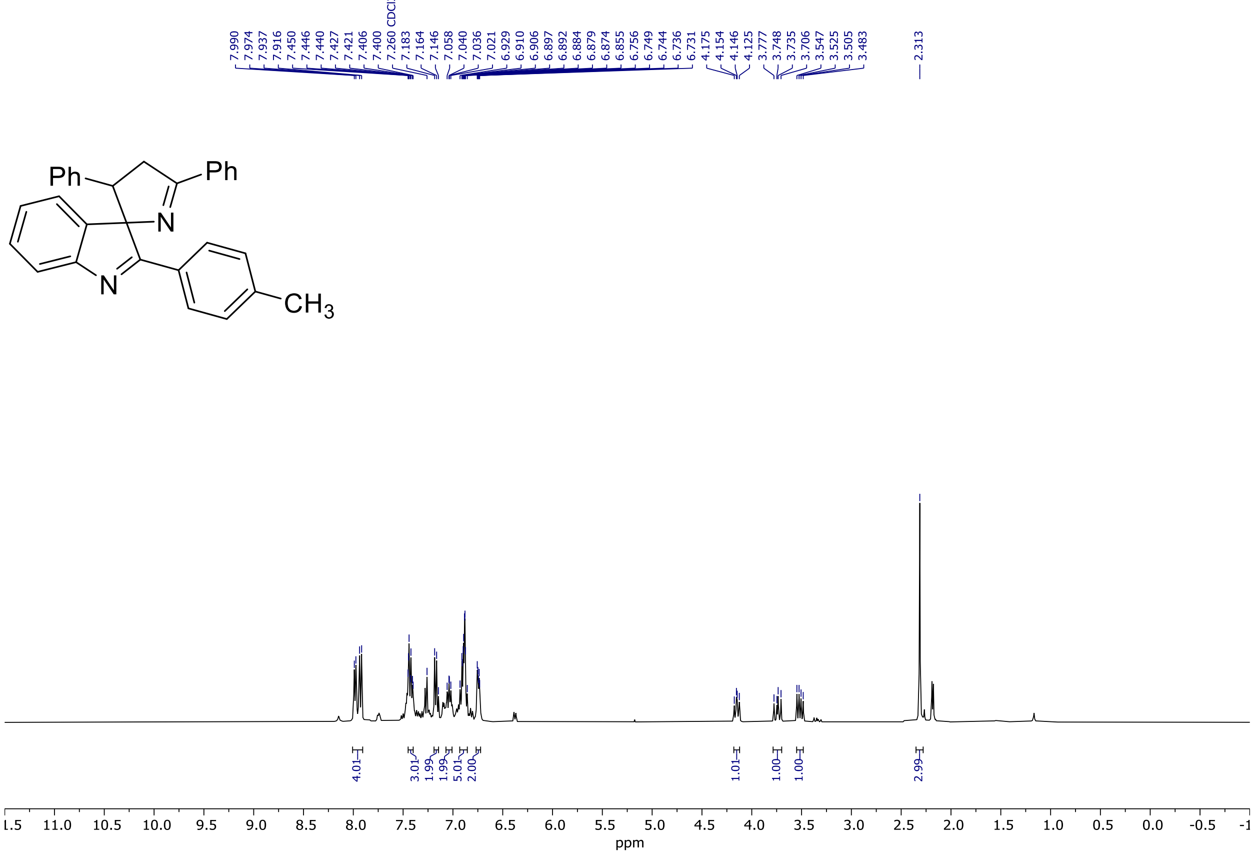

$^{13}C\{^{1}H\}$, $CDCl_3$, 100 MHz

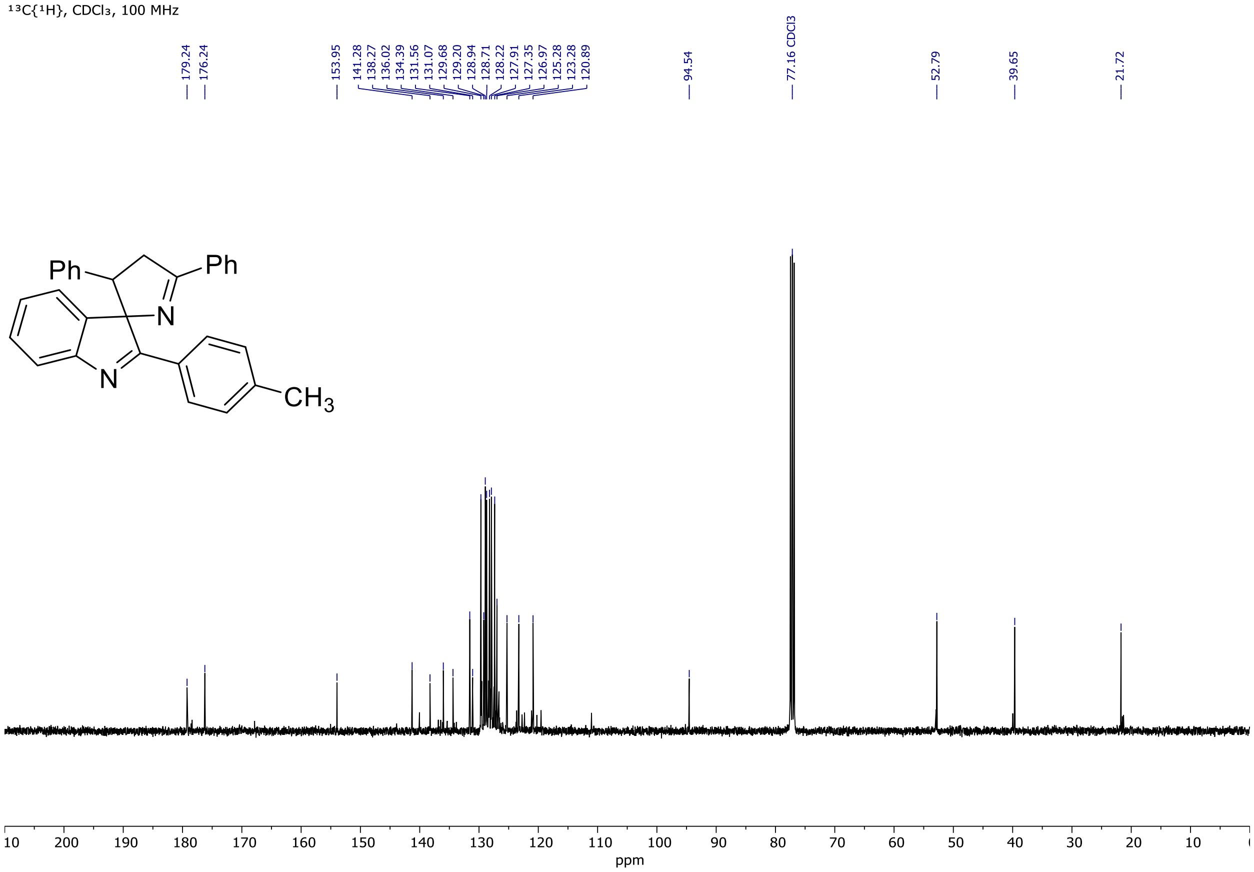

2-(4-Chlorophenyl)-3',5'-diphenyl-3',4'-dihydrospiro[indole-3,2'-pyrrole] (4i).

^{1}H, CDCl$_3$, 400 MHz

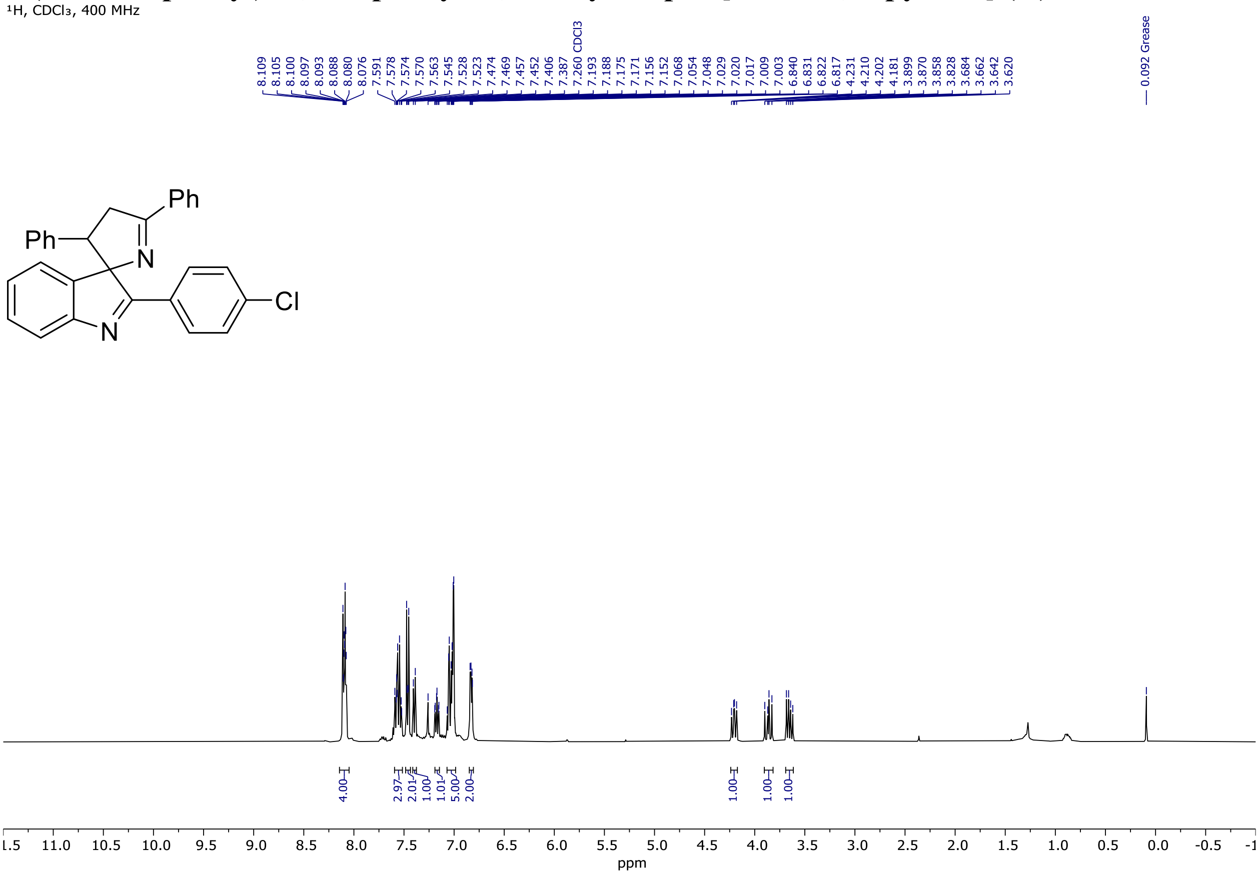

^{13}C{^{1}H}, CDCl$_3$, 100 MHz

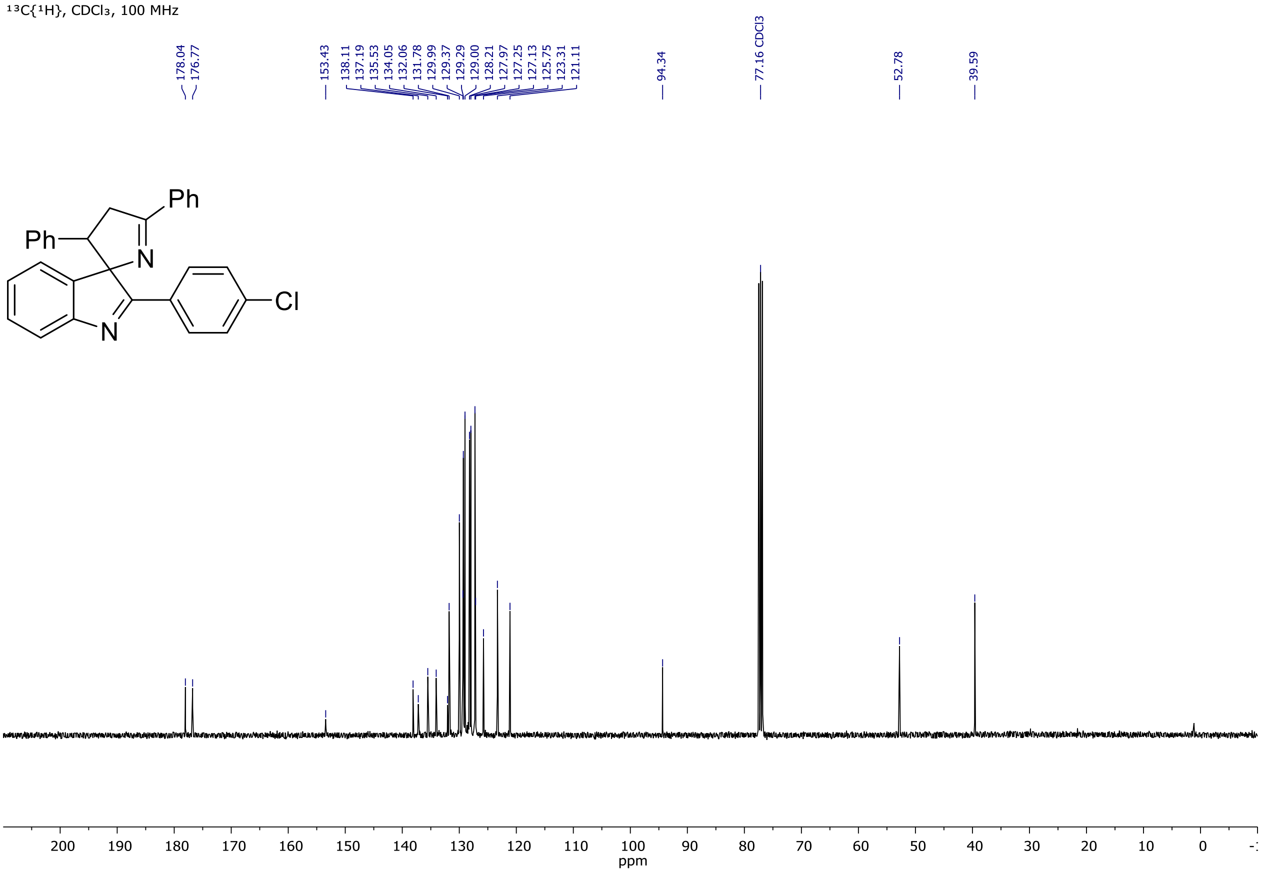

2-(4-Methoxyphenyl)-3',5'-diphenyl-3',4'-dihydrospiro[indole-3,2'-pyrrole] (4j).

^{1}H, $CDCl_3$, 400 MHz

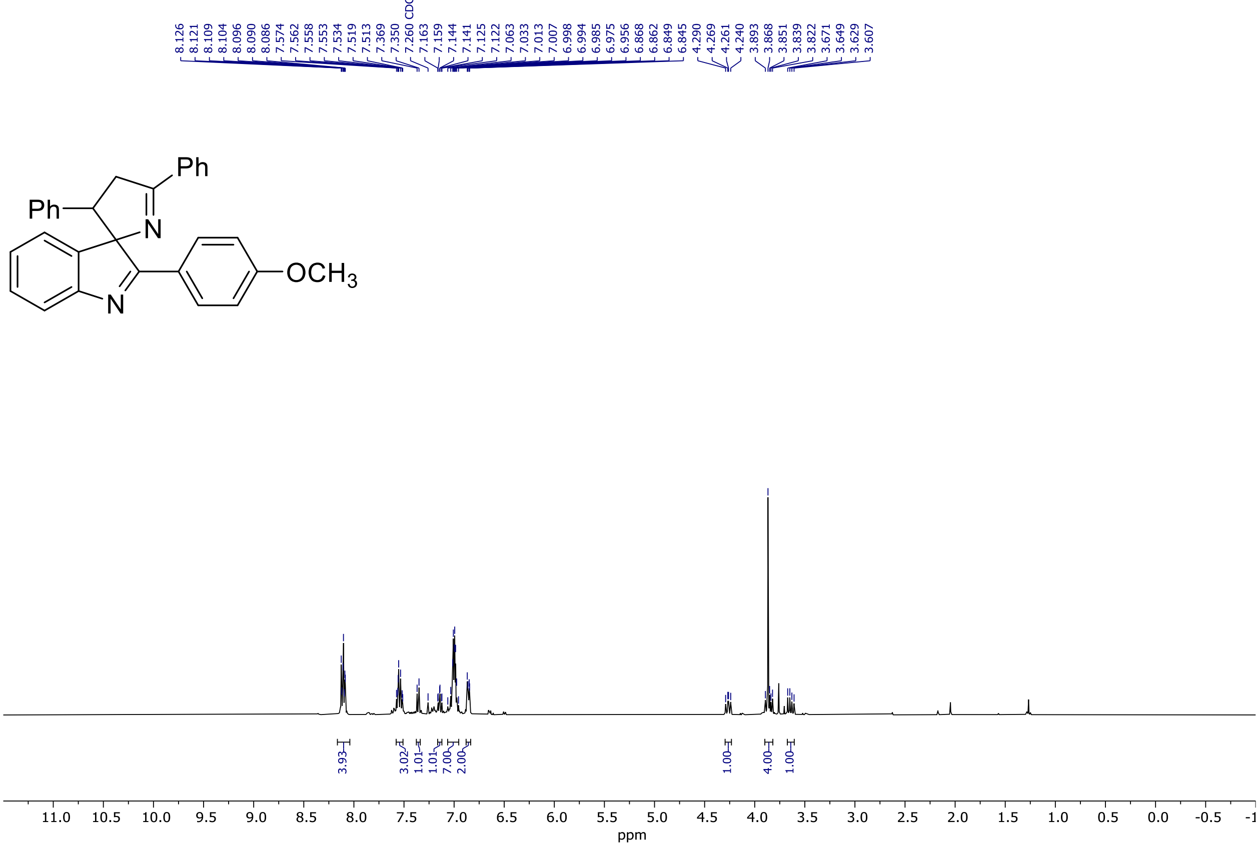

$^{13}C\{^{1}H\}$, $CDCl_3$, 100 MHz

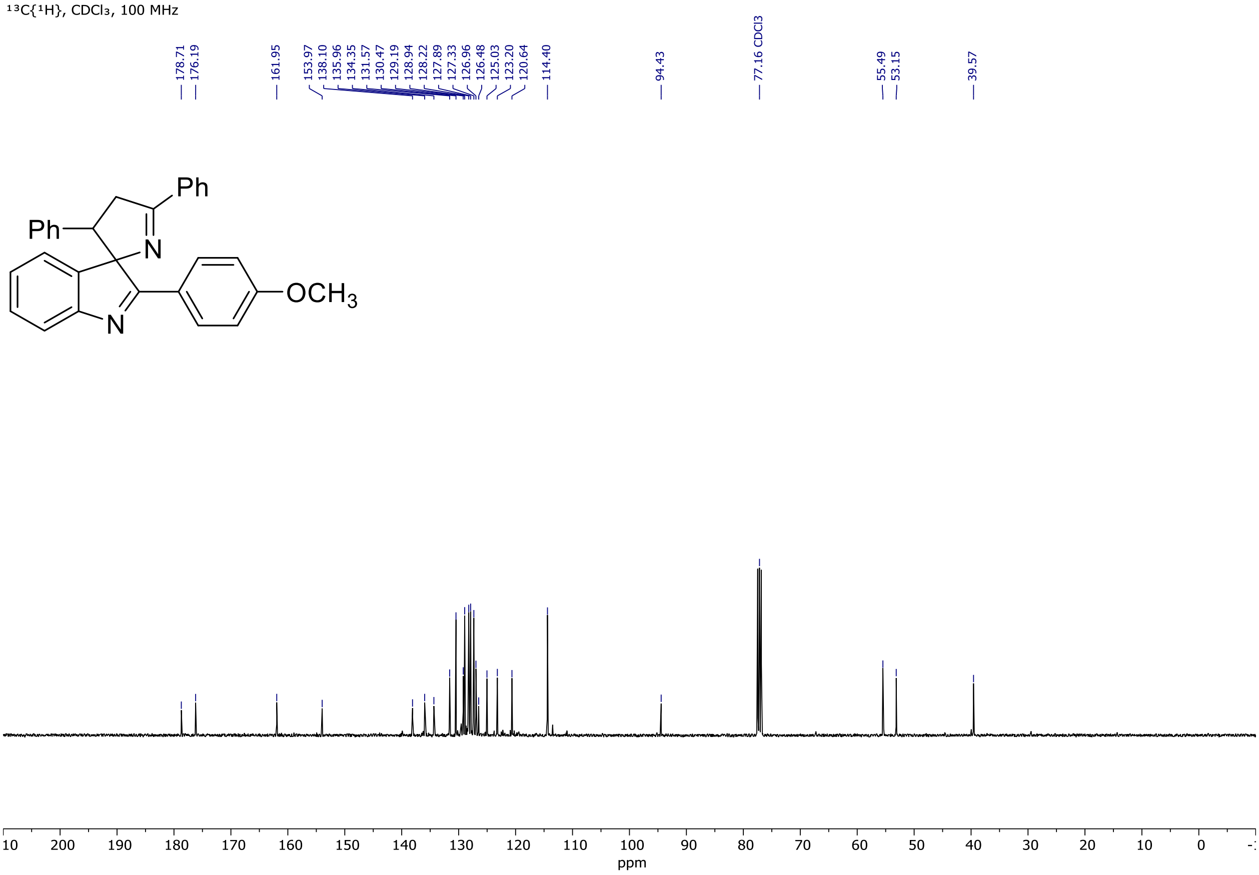

4-(3',5'-Diphenyl-3',4'-dihydrospiro[indole-3,2'-pyrrol]-2-yl)phenol (4k).

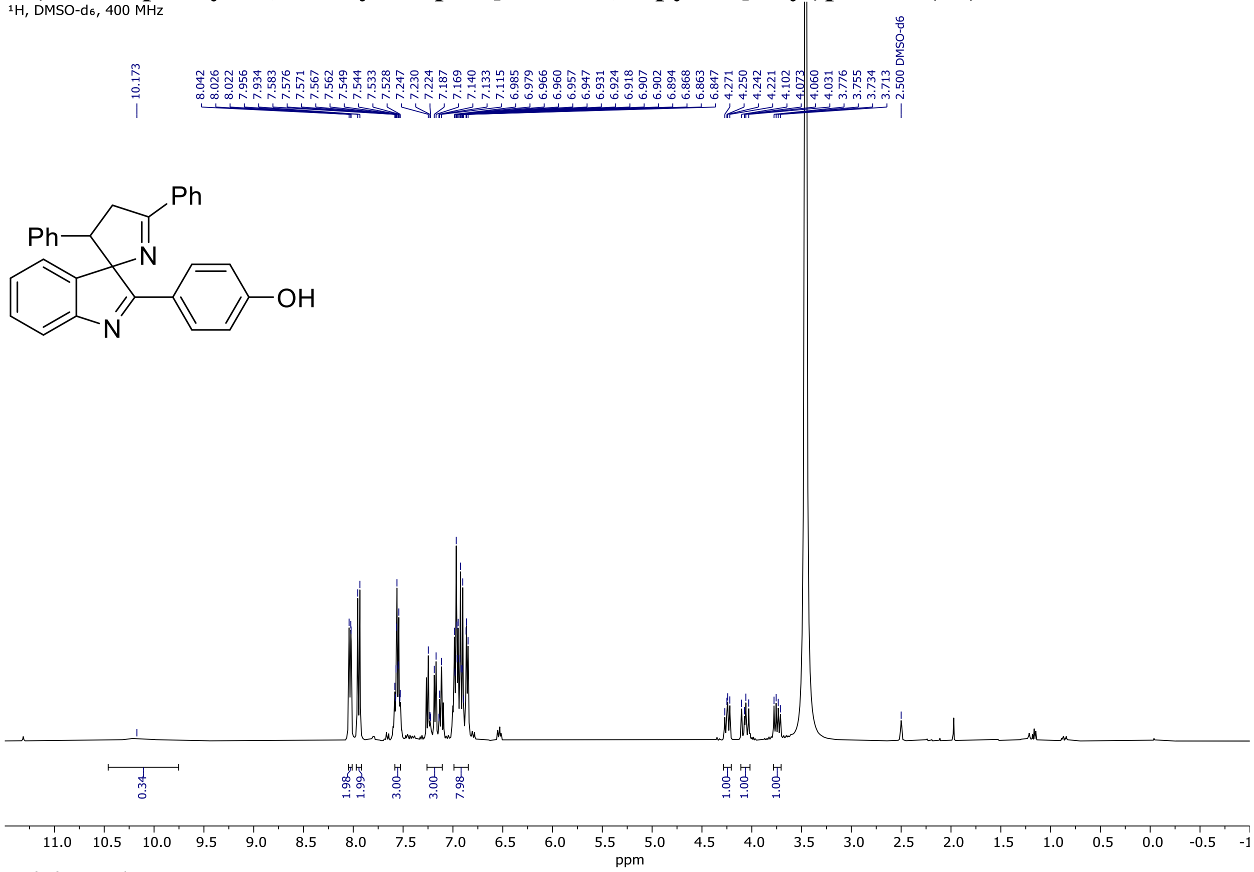

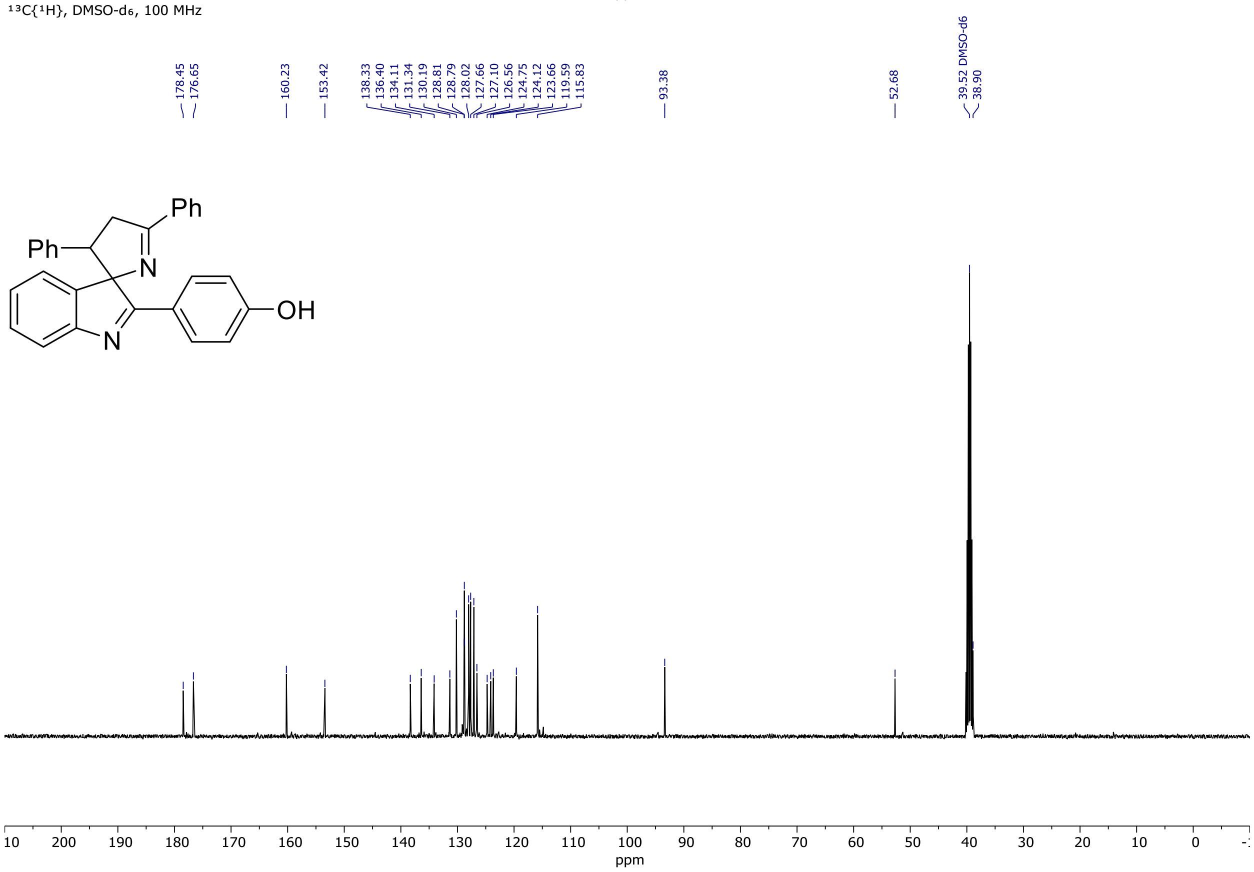

5'-(Naphthalen-2-yl)-3'-phenyl-2-(*p*-tolyl)-3',4'-dihydrospiro[indole-3,2'-pyrrole] (4n).

^{1}H, $CDCl_3$, 400 MHz

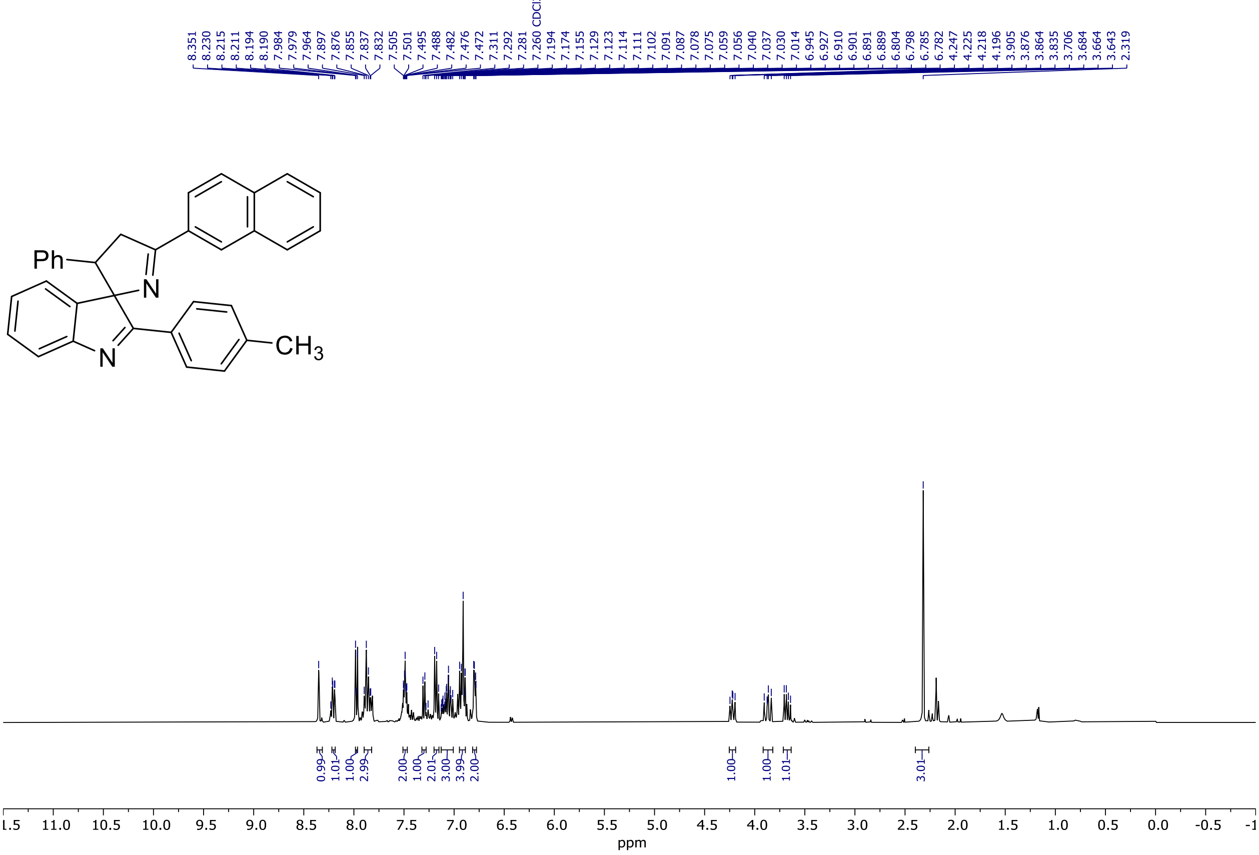

$^{13}C\{^{1}H\}$, $CDCl_3$, 100 MHz

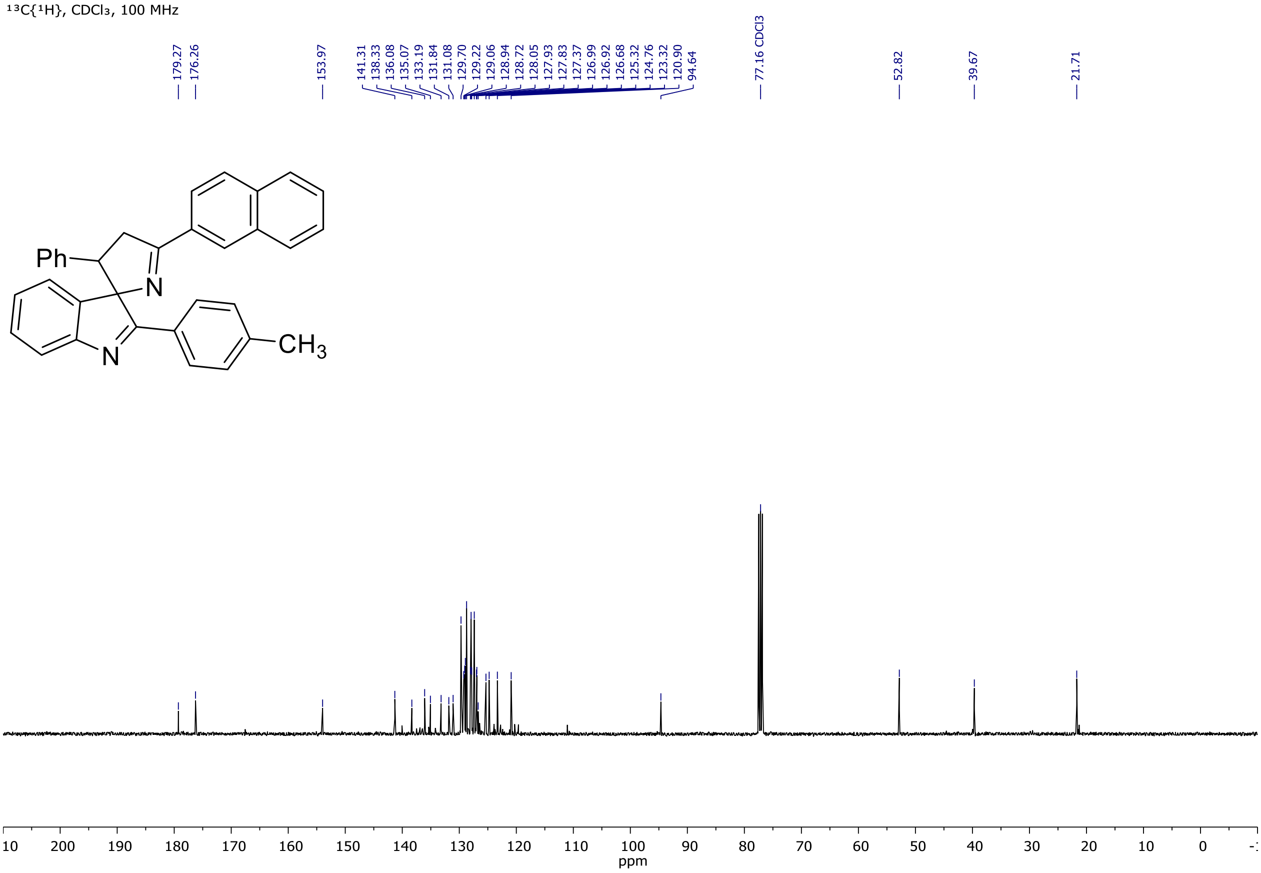

5'-Methyl-3'-phenyl-2-(*p*-tolyl)-3',4'-dihydrospiro[indole-3,2'-pyrrole] (4o).

^{1}H, DMSO-d$_6$, 400 MHz

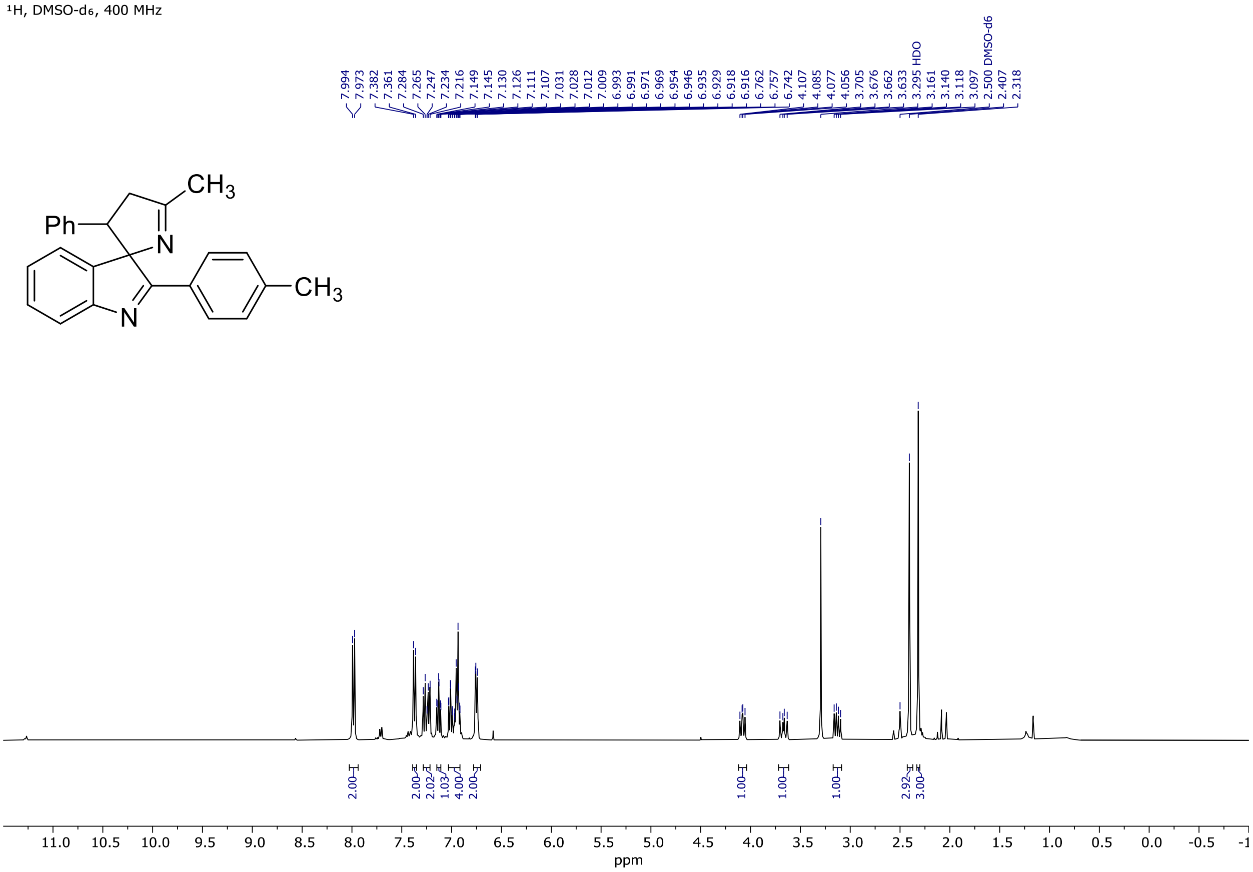

^{13}C{^{1}H}, DMSO-d$_6$, 100 MHz

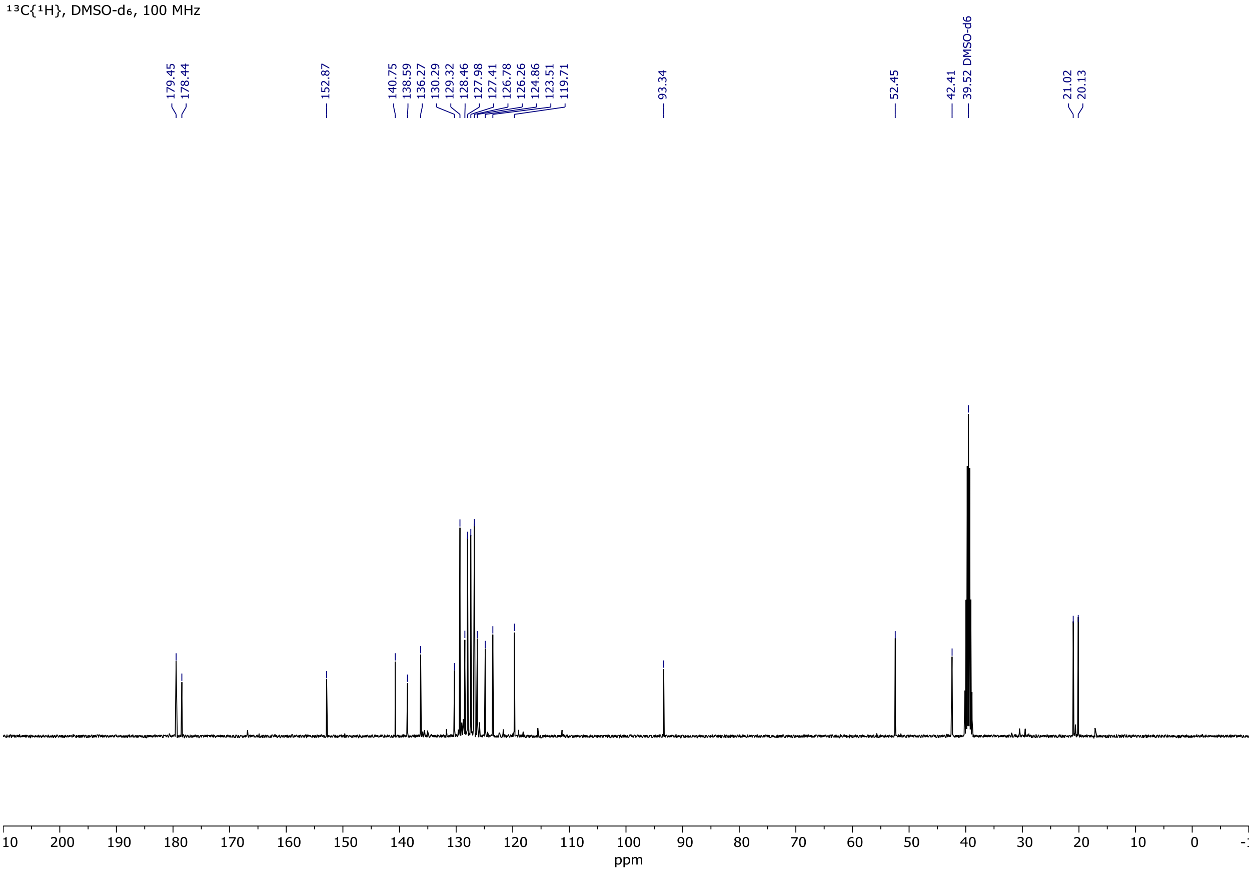

Supplement: File 1 — General reaction procedures, compound characterization data, and copies of NMR spectra. [file Beilstein_J_Org_Chem-21-2383-s001.pdf]
